# Ligand binding *Pro*‐miscuity of acylpeptide hydrolase, structural analysis of a detoxifying serine hydrolase

**DOI:** 10.1002/pro.70320

**Published:** 2025-10-11

**Authors:** Anna J. Kiss‐Szemán, Luca Takács, Imre Jákli, Zoltán Bánóczi, Naoki Hosogi, Daouda A. K. Traore, Veronika Harmat, András Perczel, Dóra K. Menyhárd

**Affiliations:** ^1^ Laboratory of Structural Chemistry and Biology, Institute of Chemistry, Eötvös Loránd University Budapest Hungary; ^2^ HUN‐REN—ELTE Protein Modelling Research Group Budapest Hungary; ^3^ Department of Organic Chemistry Institute of Chemistry, ELTE Eötvös Loránd University Budapest Hungary; ^4^ HUN‐REN—ELTE Research Group of Peptide Chemistry Budapest Hungary; ^5^ Electron Microscopy Application Department, EM Business Unit, JEOL Ltd Tokyo Japan; ^6^ Materials and Structural Analysis Division, Thermo Fisher Scientific Eindhoven The Netherlands; ^7^ Medicinal Chemistry Research Group, HUN‐REN Research Centre for Natural Sciences Budapest Hungary

**Keywords:** acylpeptide hydrolase, acyl‐pocket, cryo‐EM, detoxifier, organophosphate, serine hydrolase, serine‐protease inhibitor, substrate‐specificity

## Abstract

Acylpeptide hydrolase (APEH) or acylaminoacyl‐peptidase (AAP) is a serine hydrolase that regulates protein metabolism. It can also bind to and process unusual substrates, acting as a detoxifier. To better understand its promiscuous specificity, we determined the cryo‐EM structures of mammalian APEH complexed with classical serine protease partners: a chloromethyl‐ketone (CMK) inhibitor, an organophosphate (OP) pesticide (dichlorvos), and benzenesulfonyl‐fluoride. Since CMK derivatives of *N*‐acetylated peptides were suggested to induce apoptosis by inhibiting APEH, while OP complexes may serve as biomarkers of OP exposure and are linked to cognitive enhancement, these complexes carry physiological significance. We identified a unique strand‐breaker Pro residue in the hydrolase domain, which relaxes the active site into a partially inactivated but more spacious conformation, transforming the classical serine protease apparatus into a versatile yet potent hydrolysis center with broad specificity, distinguishing the mammalian enzyme not only from other APEHs but also from serine α/β hydrolases sharing essentially the same fold.

## 1 INTRODUCTION

The function of serine hydrolases has been under scrutiny for almost a century, and since the first structure determination of chymotrypsin (Matthews et al., 1967), their role in various biological processes, including blood clotting, immune response, and inflammation (Heutinck et al., 2010) has been extensively studied—making them one of the best‐understood enzyme classes from both biochemical and structural perspectives. The MEROPS database (MEROPS, n.d.; Rawlings et al., 2018) lists 55 serine hydrolase families with over 200 entries for human enzymes.

A distinguishing feature of these enzymes is the presence of a specific serine in the active site, which is responsible for substrate hydrolysis via cleavage of an amide/peptide bond (amidases, proteases, peptidases) or an ester bond (esterases, thioesterases, lipases). Catalytic efficiency (coupled to the activation of the serine into a nucleophile) requires two other residues, one with a basic and another with an acidic sidechain (catalytic triad) (Simon & Cravatt, 2010). In the case of the majority of serine hydrolases, the catalytic triad is composed of the serine responsible for the nucleophilic attack, a histidine that accepts a proton from the serine hydroxyl group as a base, and an aspartate that stabilizes the positive charge on the histidine, enhancing its ability to deprotonate the serine. It is widely accepted that their substrate specificity is—overwhelmingly—determined by the shape, size, and electrostatic properties of the substrate binding pockets, a series of well‐defined cavities that accommodate the sidechain of the residues closest to the proteolytic site, allowing different serine proteases to target specific locations in a protein or peptide sequence.

The S9 serine protease subfamily, the so‐called oligopeptidases, employs a further, size‐based selection process (Kobayashi & Smith, 1987). Their active site is sequestered in an inner pocket that is formed between the catalytic hydrolase domain and a propeller domain that caps it. Access to the active triad is facilitated by large domain movements and/or the self‐assembly of monomers into intricate oligomeric structures creating complex channels and shutters substrate screening systems (Kiss‐Szemán, Stráner, et al., 2022; Kiss‐Szemán, Takács, et al., 2022).

Here we focus on acylpeptide hydrolase (APEH), or acylaminoacyl‐peptidase (AAP), an oligopeptidase. The most prominent physiological function of APEH is the removal of *N*‐acetylated amino acids from the *N*‐terminus of oligopeptides and proteins (Perrier et al., 2005) thereby exerting crucial control over the proteasomal degradation of damaged and misfolded proteins (Adibekian et al., 2011; Hoernstein et al., 2023; Hwang et al., 2010; Palmieri et al., 2011; Sandomenico et al., 2021; Shimizu et al., 2003; Shimizu et al., 2004; Tangri et al., 2021). This places APEH among the detoxification enzymes of our physiology—known for their substrate promiscuity. These enzymes are usually not part of well‐defined metabolic pathways, but rather act in response to various stress events and process structurally diverse compounds; their multi‐purpose hydrolytic activity also often leads to drug metabolism that is just “collateral damage” (Atkins, 2020). The plastic, adaptable active sites of detoxification systems allow for induced‐fit and for conformational selection mechanisms during substrate binding and catalysis (Honaker et al., 2011). This flexibility is essential for fulfilling their role, justifying why evolution—in their case—has favored maintaining, rather than eliminating enzymatic promiscuity.

Perhaps the most surprising example of APEH's wide and unexpected substrate range is presented by a drug–drug interaction it is responsible for. It has been shown that mammalian APEH is inhibited by the covalent binding of carbapenem‐type antibiotics (Kiss‐Szemán, Takács, et al., 2022)– an interaction that has not been observed for any other mammalian serine hydrolase. This inhibition, concurrently, also results in the loss of the therapeutic effect of valproic acid (VPA), a widely used drug in the treatment of various neurological conditions (Mishra et al., 2022). APEH plays a unique role in maintaining therapeutic serum levels of VPA by hydrolyzing its primary metabolite, valproic acid‐β‐(D)‐glucuronide (VPA‐G), leading to the reabsorption of active VPA into the circulation. That APEH is again the only serine hydrolase able to perform this function is demonstrated by the fact that when carbapenem antibiotics inhibit APEH, a significant decrease in the serum VPA concentration and the abolition of its beneficial effects has been observed (Deshayes et al., 2017; Mori et al., 2007; Nagai et al., 1997). In fact, the clearance of VPA in the absence of APEH is so rapid that carbapenem antibiotics are now used in the emergency treatment of VPA‐overdose (Cunningham et al., 2022; Dreucean et al., 2019; Thomas et al., 2020).

The most recent discovery concerning APEH describes its central role in the development of a new class of resistance evading pro‐drugs for the treatment of malaria. The malaria parasite *Plasmodium falciparum*, which invades the red blood cells of the host, incorporates human APEH, which remains active in the cytosol of *P. falciparum* and can be targeted by lipophilic esters of phosphonic acid antibiotics because the pro‐drug lipophilic moiety is cleaved by APEH, resulting in the in situ release of the active antibiotic (Sundararaman et al., 2025).

We have previously determined the first structure of a mammalian APEH, that of porcine liver APEH (pAPEH), in its ligand‐free and carbapenem‐inhibited forms (Kiss‐Szemán, Stráner, et al., 2022; Kiss‐Szemán, Takács, et al., 2022), but to be able to form a more grounded picture of its substrate‐binding modes and capabilities, here we set out to study its complexes with classical serine protease inhibitors: a chloromethyl‐ketone derivative, a benzenesulfonyl fluoride (AEBSF), and an organophosphorous (OP) pesticide molecule. This latter holds special significance since APEH was shown to be a more sensitive receptor of OPs both in blood (Marsillach et al., 2011; Marsillach et al., 2013; Quistad et al., 2005) and the brain (Kim et al., 2010; Richards et al., 1999; Richards et al., 2000) than acetylcholine‐esterase, the primary target responsible for their toxicity. Inhibition of APEH by OPs is so effective that exposure to certain pesticides can be detected weeks after the event, through monitoring the APEH activity of red blood cells (Schopfer et al., 2010). Comparison of the structures of ligand‐free and covalently inhibited forms of pAPEH with similar complexes of other serine hydrolases allowed for the identification of the structural features that distinguish the catalysis of the promiscuous APEH from that of the canonical proteases. We found that a proline residue present in the extended core β‐sheet of the hydrolase domain of mammalian APEH (see Figures S10 and S12) causes relaxation of the active site into partially inactivated (Kiss‐Szemán, Takács, et al., 2022) but more accessible conformations, but also a coupled increase in flexibility that allows for its rearrangement into the catalytically competent form. Thus, this single sequence modification transforms the classical serine protease apparatus into a versatile but still potent functional hydrolysis center with quite ambiguous specificity (see Data S1, chapter I: “Promiscuity of mammalian APEH”).

## 2 RESULTS

### 2.1 APEH in complex with chloromethylketone inhibitor—Binding of a canonical inhibitor via a non‐canonical rearrangement

**FIGURE 1:**
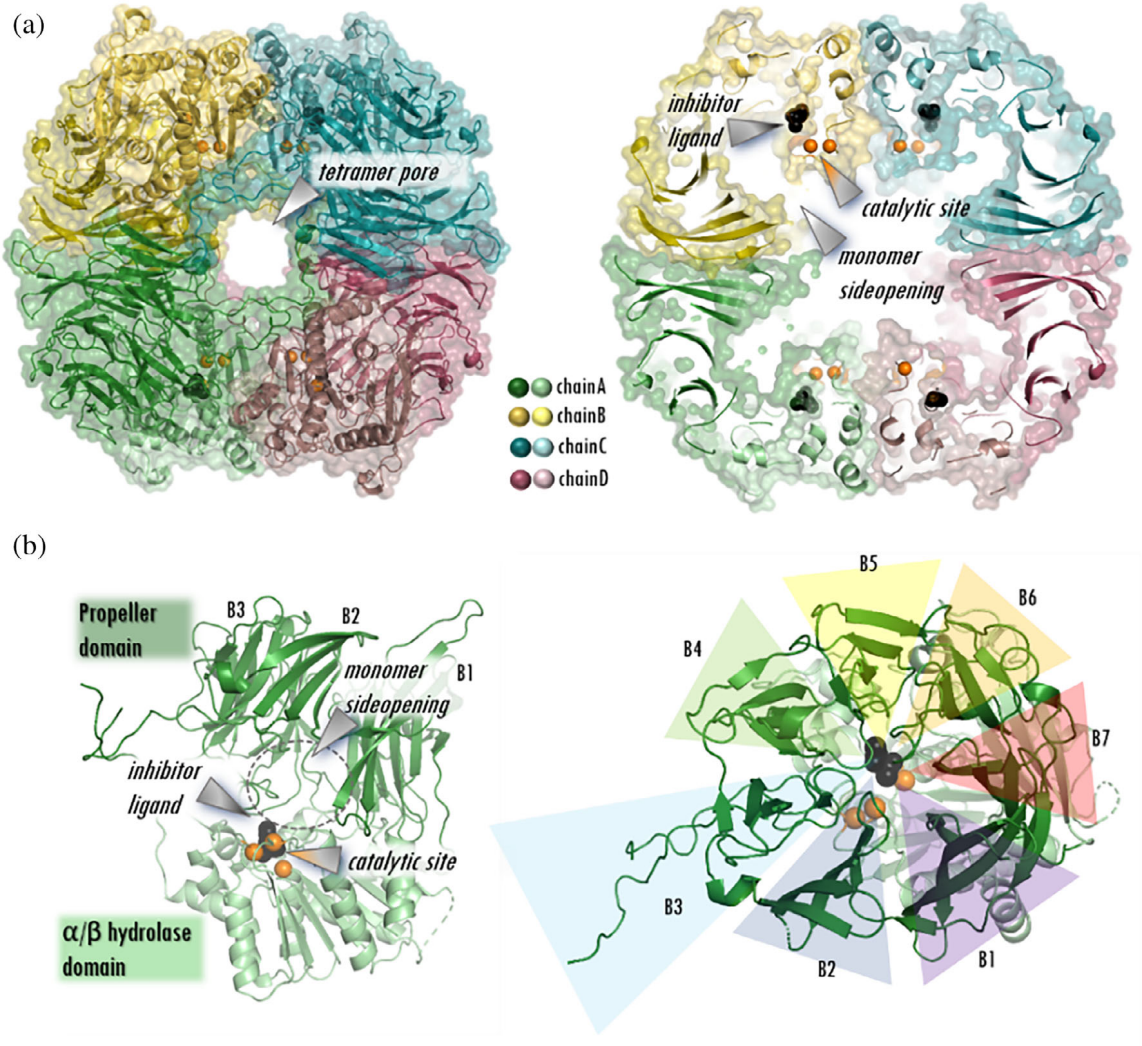
The tetrameric structure of pAPEH. (a) The tetramer of pAPEH with the large entrance pore that allows access to the central antechamber of the complex (Kiss‐Szemán, Stráner, et al., 2022). Its size is regulated by flexible loops carrying charged residues. A cross‐section of the complex showing the location of the catalytic apparatus (Ser, His, Asp catalytic triad shown in orange), the four ligand binding sites (black) and the side opening of the monomers. (b) Sideview of pAPEH showing the two domain build‐up of the monomers (propeller domain and α/β hydrolase domain) and a top view of the 7‐bladed propeller domain (blades numbered from B1 to B7).

**FIGURE 2:**
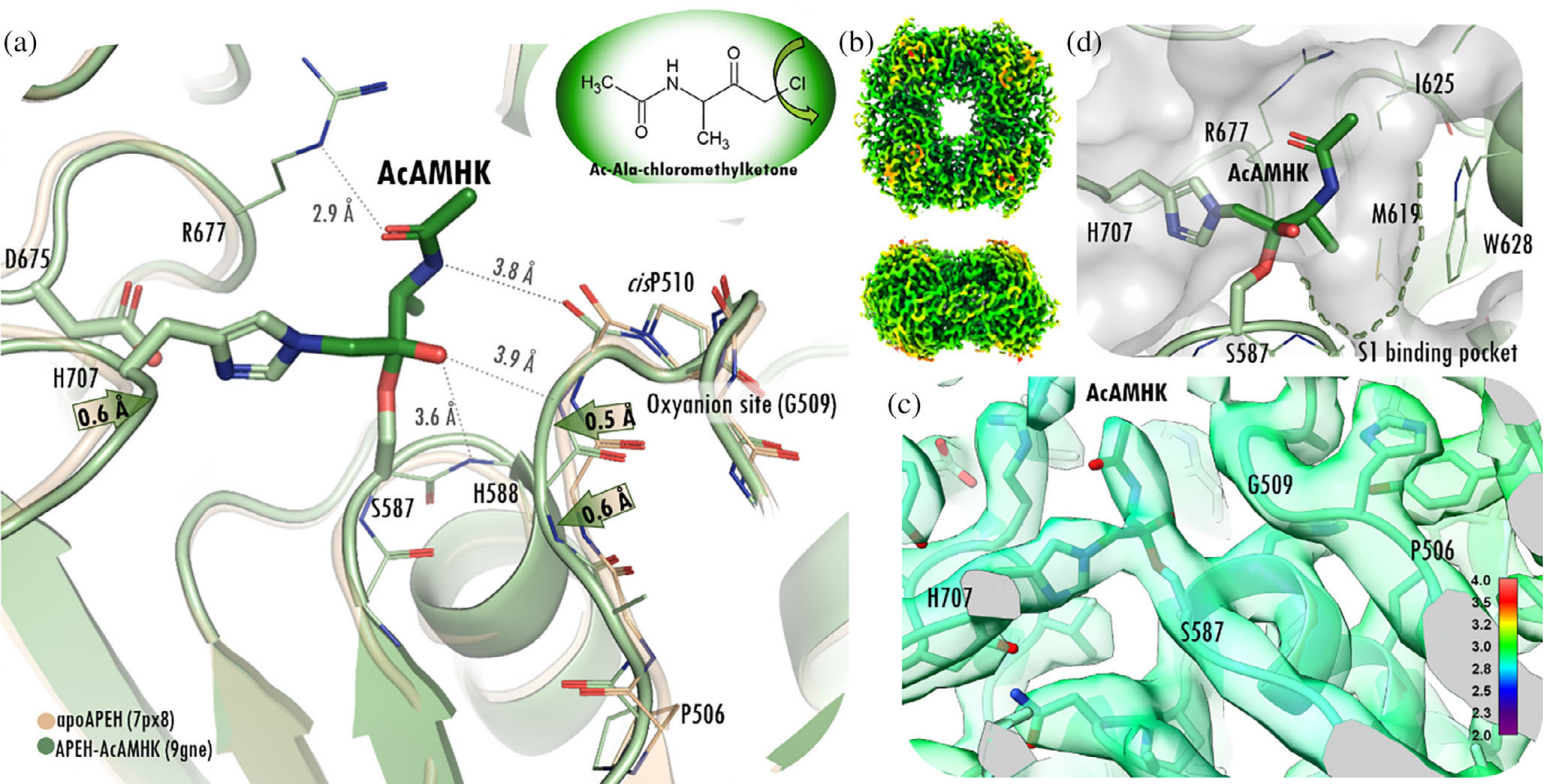
The structure of pAPEH in complex with a covalent substrate analogue inhibitor. (a) Acetyl‐alanyl‐chloromethyl ketone (AcACMK) covalently binds to the active site of APEH (Ser587 and His707), forming an acetyl‐alanyl‐methylhemiketal derivative (AcAMHK) of the enzyme. The oxyanion‐loop (residues 508–511) is shifted toward the ligand, and the catalytic His707 loop also moves closer as a result of the covalent bond formation. (b, c) Local resolution colored maps of pAPEH tetramer (top/sideview; b) and near the active site (c) calculated by Phenix (Liebschner et al., 2019) contoured at the 0.0581 threshold level (color scheme is shown in panel c). (c) The local resolution value for the AcAMK ligand and the oxyanion‐loop is 2.7 Å (turquoise). (d) Molecular surface of the catalytic cavity showing the S1 binding pocket (green dotted line) accommodating the Ala sidechain.

Chloromethyl ketone derivatives form complexes with serine proteases that resemble the tetrahedral 1st intermediate of the catalytic process by forming covalent bonds with both the catalytic Ser and His (Figure 1) (Powers et al., 2002). Here we determined the tetrameric structure of pAPEH in complex with acetyl‐alanyl‐chloromethyl ketone (AcACMK), a covalent inhibitor using cryo‐EM with a maximum resolution of 3.19 Å (Figures 1, 2, S1, and S2, Table S1).

The local resolution was calculated from half‐maps by Phenix (Powers et al., 2002) indicating an overall resolution range of 1.4–3.2 Å and a mean resolution of 2.7 Å for the tetramer (Figure 2b). This resolution also applies to the core of the hydrolase domain and the active site and its surrounding (Figure 2c).

The AcACMK inhibitor hydrolyzed into acetyl‐alanyl‐methyl‐hemiketal (AcAMHK) and bound covalently to the active Ser587 and His707 (Figure 2a) of each monomer of the tetrameric pAPEH structure. The oxygen of the tetrahedral center (most likely an *OH* group in the stable form of the complex) (Figure 2a) is oriented by the oxyanion site—the backbone amides of Gly509 and His588—similarly to what is expected during catalysis, however, neither distance (3.9 and 3.6 Å, respectively) is close enough for the formation of a proper *H*‐bond. This was a surprising finding, since these two hydrogen bonds are the signature interactions of the oxyanion pocket of serine proteases with their substrates. The amide *NH* moiety of AcAMHK points toward carbonyl oxygen of Gly509, which is—again—similar to those seen in substrate complexes of serine protease enzymes, but here it not quite close enough (3.8 Å) for a strong interaction. The acetyl group of AcAMHK is coordinated by the *Nε‐*atom of Arg677, which flips to fulfill this interaction (as compared to its position in the ligand‐free form of the enzyme). The small Ala sidechain of the inhibitor sits in the shallow, rather hydrophobic S1 binding pocket (Figure 2d).

Compared to the uncomplexed structure of pAPEH (PDB id 7px8), a slight rearrangement of the oxyanion‐loop can be observed, with the backbone *N* and *C*α of Gly509 shifting toward the inhibitor by 0.5 and 0.6 Å, respectively (measured after fitting the core structure of the hydrolase domains: the backbone of the eight β‐strands that form the central sheet and that of the helix which starts immediately following the catalytic Ser). Furthermore, the loop holding the catalytic His707 also shifts closer to the inhibitor by 0.5 Å, as measured at His707(*C*α). It seems as if the too spacious active site of the ligand‐free form would attempt to move closer to activate the enzyme sufficiently for binding the inhibitor, but the resultant active site pocket still seems quite a bit looser than in the case of the canonical binding mode.

### 2.2 Evaluation of the potential catalytic role of the oxyanion‐loop displacement

**FIGURE 3:**
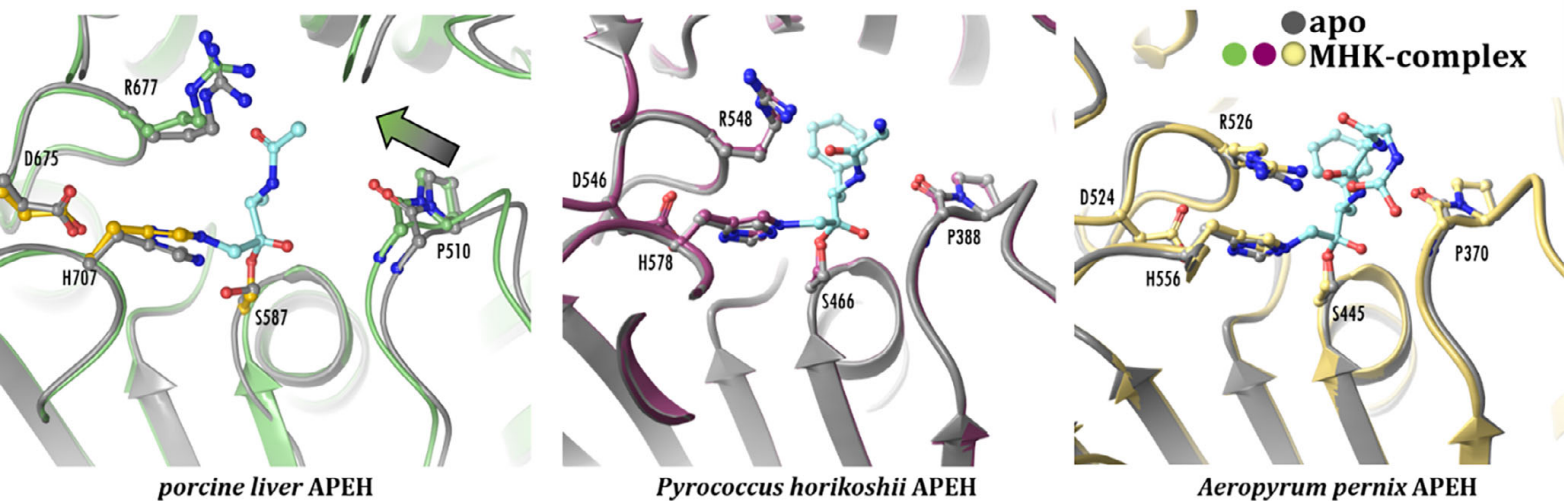
Structural changes of the active sites of different APEH enzymes upon binding a chlorometyl ketone inhibitor. Superimposed structures of the resulting covalent complexes and the structure and their respective ligand‐free forms are shown. A shift (indicated by the gray‐green arrow) of the oxyanion‐loop is apparent upon formation of the pAPEH‐AcA‐MHK complex (left, present work), whereas no shift was observed in case of the *Ph*APEH‐(Z)GGF‐MHK (middle, PDB id 4hxf and 4hxe) and *Ap*APEH‐(Z)GGF‐MHK (right, PDB id 4re6 and 3o4g) complexes.

A comparison of the ligand‐free and CMK‐inhibitor‐bound structures of archaeal APEHs (*Ph*APEH and *Ap*APEH, Figure 3) revealed that the shift of the oxyanion loop upon complex formation seems to be a distinctive feature of the mammalian APEH structure. Conversely, it seems that archaeal variants of APEH contain a pre‐formed reactive center that requires no adjustment upon ligand binding.

**FIGURE 4:**
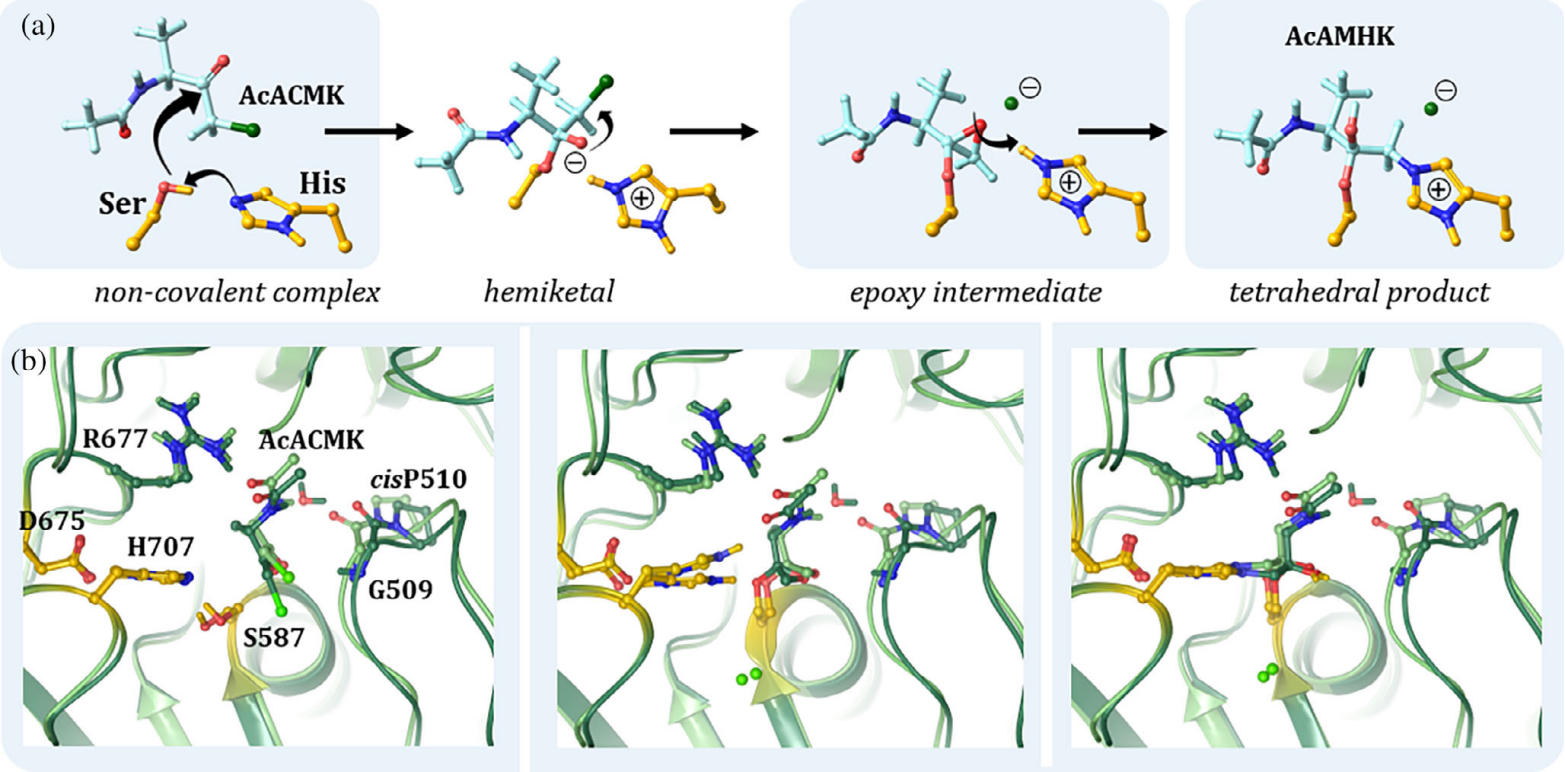
Covalent complex formation between pAPEH and AcACMK. (a) The steps of complex formation—with the considered states indicated by the blue boxes. (b) Calculated structures of the reactant state (left), the epoxy intermediate (middle) and the tetrahedral acyl‐enzyme (in this calculation this is the product) state (right). The model system with the direct *H*‐bond between the forward shifted oxyanion‐loop is shown in lighter green, while the conformations where a water is wedged between the two, in darker green (the C atoms of the catalytic triad are shown in yellow). Relative energies were calculated (B3LYP/6‐31G**) for the epoxy‐intermediate (middle): 2.4 kcal/mol for the model with direct *H*‐bond (light green) and 2.6 kcal/mol for the water‐wedged model (dark green); and for the tetrahedral product state: −17.8 and − 44.1 kcal/mol for the two models showing that the direct *H*‐bond forming model with the flexible oxyanion‐loop is energetically more favored.

To determine whether the shift of the oxyanion‐loop that we found between the ligand‐free and ligand‐bound forms of pAPEH is significant enough to exert an influence over its catalytic capacity, we carried out QM/MM calculations. To focus the differences of the compared models to the active site, the two systems considered were obtained from the trajectory of a restrained molecular dynamics (MD) simulation, where the backbone of the pAPEH tetramer was biased toward the conformation of the ligand‐free form: one with a direct *H*‐bond formed between the carbonyl *O‐*atom of Gly509 and the amide *NH* group of AcAMHK despite the restraints (via the slight forward shift of the oxyanion‐loop) in an arrangement that closely resembles the experimentally determined conformation of the complex, and another where a water molecule is inserted between the two with the oxyanion‐loop remaining in the relaxed conformation of the ligand‐free form (Figure S14). We calculated the conformational energies of significant states along the proposed reaction path of the complex formation process (Powers et al., 2002): the reactant state, the epoxy intermediate, and the product state (Figure 4). We found that while the relative energy of the putative epoxy intermediate was unperturbed, the product state of the reaction was considerably less favorable in the presence of the distant conformation of the oxyanion‐loop than in the model where direct association is formed between the loop and the inhibitor. The calculations thus indicate that even a modest shift of the oxyanion‐loop (by 1.1 Å, 1.2 Å, and 1.0 Å measured at the amide *H*‐atom of Gly509 in the QM/MM derived structures of the reactant, epoxy, and product states, respectively) causes a significant change in the reactivity of the catalytic machinery of pAPEH; thus, the ligand‐free form of the enzyme is indeed in a partially inactivated or latent conformation (Figure 4b).

To further probe the catalytic significance of the shift of the oxyanion loop, we produced *Ph*APEH and *Ap*APEH. Not only is the shape and position of the oxyanion loop unchanged by complex formation in these archaeal variants (see Figure 3), but it is also locked in a conformation that is very similar to its forward shifted state in pAPEH; thus, these systems can be used as functional models of the enzyme with a more restricted and confined catalytic site. We found that the archaeal variants are quite selective toward their specific substrate Ac‐Leu‐pNA, not being able to cleave Ac‐Ala‐pNA, while pAPEH processes both almost instantaneously. Furthermore, neither *Ap*APEH nor *Ph*APEH is inhibited by meropenem, the bulky carbapenem antibiotic that is a potent inhibitor of pAPEH (Kiss‐Szemán, Stráner, et al., 2022). Attempts at producing a co‐crystal structure of *Ap*APEH and meropenem were also unsuccessful—using the same protocol that afforded co‐crystals of *Ap*APEH with peptide‐like chloromethyl ketone inhibitor (Menyhárd et al., 2015; see Data S1, Chapter II: “Substrate/inhibitor preference of APEH from *Aeropyrum pernix* and *Pyrococcus horikoshii*”). Although the homology between pAPEH and its archaeal variants is low, the shape and constitution of the active site are similar except for the relaxed conformation of the oxyanion loop in the unligated state of pAPEH; thus, the difference in reactivity between the archaeal and mammalian forms might well be the consequence of the better accessibility and greater adaptability of the active site of the latter.

### 2.3 Organophosphate and benzenesulfonyl complexes of pAPEH

**FIGURE 5:**
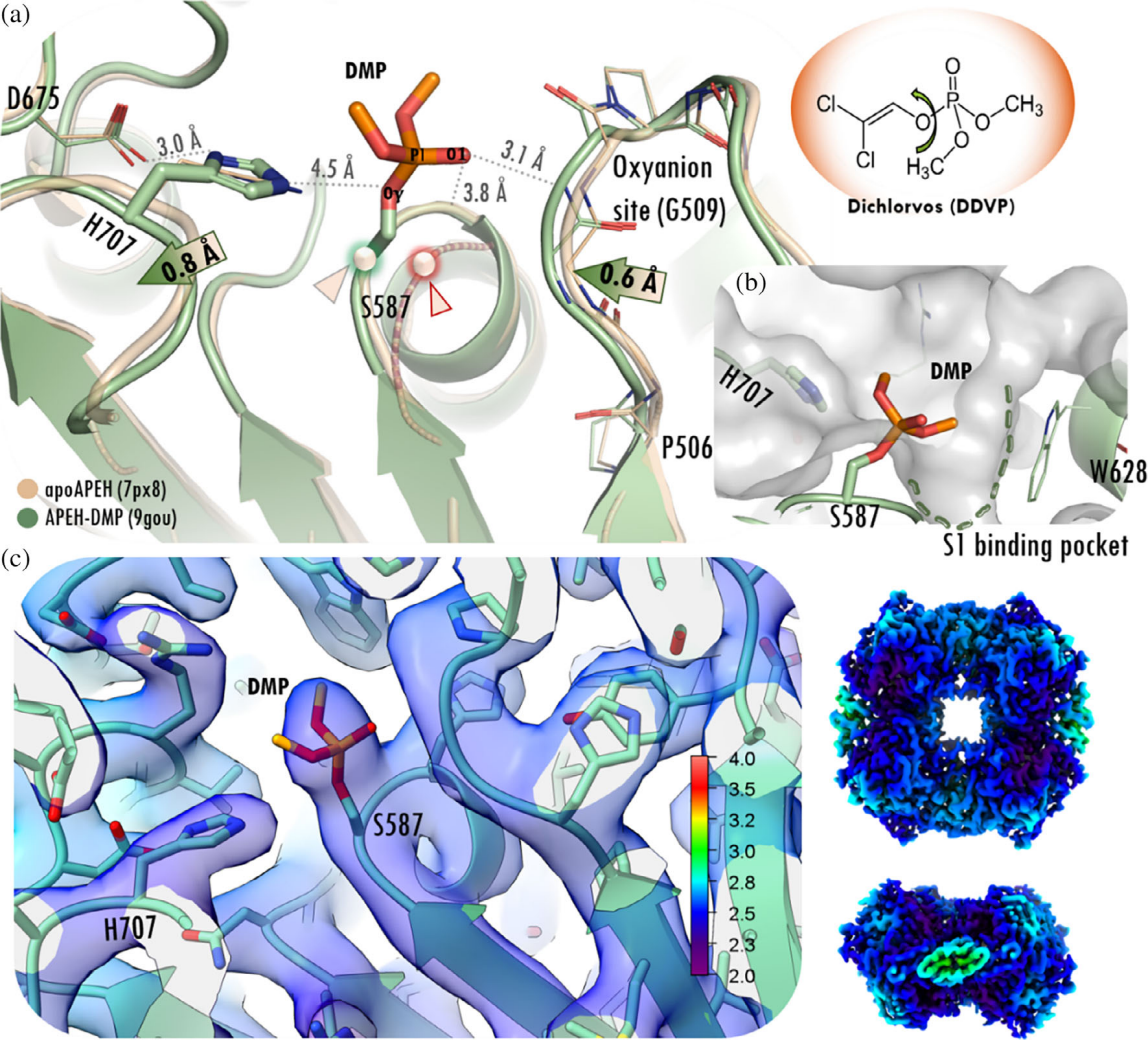
Dichlorvos hydrolyses and binds covalently to pAPEH. (a) In the structure of the complex solved by cryo‐EM (green, with the bound ligand in dark orange) the dimethoxyphosphate residue (DMP) covalently binds to the catalytic Ser of APEH. The loop holding the oxyanion site (Gly509) shifts toward the ligand, while the loop carrying the catalytic His707 moves further to accommodate the methoxy group. The structure is superimposed onto the ligand‐free form of pAPEH (light orange), where the alternate conformations of the active site serine are shown by the Cα atoms of the active Ser587, highlighted by green (active conformation) and red (inactive conformation) triangles. (b) Molecular surface of the catalytic cavity showing the S1 binding pocket (green dotted line) accommodating one of the methoxy groups. (c) Local resolution colored maps of the pAPEH‐DMP tetramer (top/sideview) and near the active site calculated by Phenix contoured at the 0.0781 threshold level. The local resolution value for the DMP ligand and the oxyanion‐loop is 2.4 Å (deep blue).

The structures of two further covalently inhibited complexes of pAPEH were determined using typical serine hydrolase inhibitors: an organophosphate compound (Casida & Quistad, 2005) and a classical sulfonyl‐fluoride serine protease inhibitor (Narayanan & Jones, 2015). The pAPEH—dimethoxy‐phosphate (DMP) complex was created by adding dichlorvos (DDVP), an organophosphate insecticide, to pAPEH. The structure of the complex was determined by cryo‐EM to 2.65 Å maximal resolution, in D2 symmetry (Figures 5, S3, S4, and Table S1). The local resolution was calculated from half‐maps by Phenix, indicating an overall resolution range of 1.1–2.7 Å and a mean resolution of 2.3 Å (Figure 5c). As indicated by the deep blue color, this resolution also applies to the core of the hydrolase domain, including the active site and its surrounding.

Dimethoxy‐phosphate (DMP) residue of dichlorvos is bound covalently to the active Ser587 (*O*γ‐*P*1) of APEH (Figure 5a). The free oxygen of the phosphate (*P*1‐*O*1 moiety, Figure 5a) is fixed and oriented by the oxyanion site. Here—in contrast to the pAPEH‐AcAMHK complex—one *H*‐bond is actually formed with the oxyanion‐loop (with the amide *N*H of Gly509), while the other interaction partner (the amide *N*H of His588) is still too far for a strong association. One of the methoxy groups is positioned within the S1 binding pocket (Figure 5b). Here no *H*‐bond with Arg677 is formed, so the Arg677 sidechain remains in the rotameric form seen in the structure of the uncomplexed form of the enzyme (PDB id 7px8), forming a salt‐bridge with Asp624 (distance between Arg677(*N*ε) and Asp624(*O*D1) is 3 Å). Compared to ligand‐free pAPEH, the shift of the oxyanion‐ and the His‐loops can be seen: the oxyanion‐loop moves closer to the inhibitor with the backbone *N* and *C*α of Gly509 shifting by 0.6 and 0.7 Å, respectively, in similar way to that seen in the pAPEH‐AcAMHK complex, however, the catalytic His‐loop moved in the opposite direction (by 0.8 Å measured between His707(*C*α)s), shifting away from the catalytic center. We found DMP bound in a non‐aged form—meaning that it retains both of its methoxy substituents. Aging is the spontaneous dealkylation process that often occurs in organophosphates bound to serine proteases, where the gradual loss of the *O*‐alkyl (or *N*‐alkyl) groups increases the irreversibly bound nature of the complex. Since the methoxy groups of DMP are effective leaving groups, it may undergo hydrolysis to form an aged product—a negatively charged phosphate—if sufficiently long time is provided for the reaction.

The cryo‐EM structure of pAPEH treated with AEBSF, a sulfonyl‐derivative serine‐protease inhibitor (Table 1 and Figures S5 and S6), showed similar rearrangements to those of the OP‐complex. The oxyanion loop shifted by 0.4 Å (toward the ligand) and the His‐loop by 0.2 Å (shifting away). The local resolution was calculated from half‐maps by Phenix, indicating an overall resolution range of 1.3–3.2 Å and a mean resolution of 2.61 Å, but with only partial density present on the ligand (local resolution 2.9 Å), which makes the interpretation of the binding mode of the inhibitor ambiguous. It can, however, be determined with certainty that it binds covalently to the catalytic serine, and one of the sulfonyl oxygen atoms is within *H*‐bonding distance to one or both of the NH groups of the oxyanion site. This is in contrast to that found in the case of dipeptidyl‐peptidase 4 (DPP‐IV, another member of the S9 protease family) where AEBSF was found bound not to the active Ser, but to a nearby Tyr (Narayanan & Jones, 2015). pAPEH carries a His (H507) at the corresponding position, which is turned toward the inside of the oxyanion loop and forms stabilizing interactions with its backbone carbonyls, and there are no accessible Tyr residues within the sheltered active site—thus the inhibitor directly coordinates to the active Ser.

**TABLE 1:** Key distances (average values where applicable) of the active site of ligand‐free, organophosphate‐bound, and CMK‐derivatized serine hydrolases of α/β hydrolase superfamily.

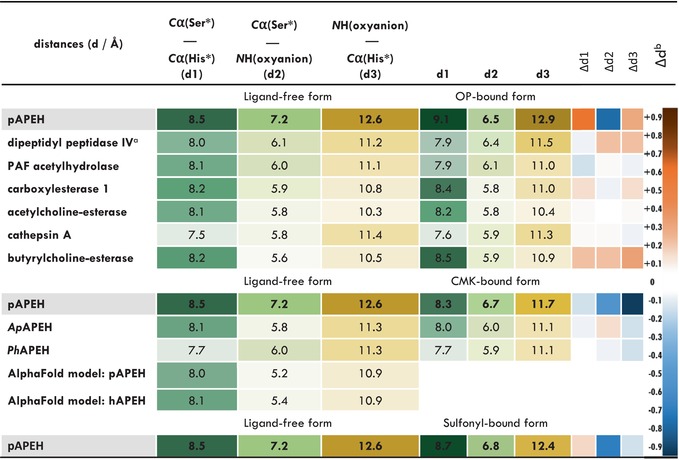

*Note*: The size of each active site was described using three distances forming a triangle: The distance of the catalytic Ser and His measured between their *C*α atoms (d1), the distance between the *C*α (Ser*) and the distal oxyanion coordinating *H*‐bond donor atom nitrogen of the oxyanion‐loop (d2), backbone *N* of Gly509 in case of pAPEH, *N*H (oxyanion) and the distance between this nitrogen‐bond donor atom of the oxyanion‐loop and the *C*α of the catalytic His (d3) (For individual values and PDB codes of the structures see Table S2). Darker colors indicate longer, while lighter colors indicate shorter distances. “*” the asterisk denotes the members of the catalytic triad.

^a^In dipeptidyl peptidase 4 the characteristic oxyanion‐loop of α/β hydrolases is shifted and coordination the oxyanion is carried out by the hydroxyl group of Tyr547, which replaces the loop, so in this case the Tyr*O*G1 atom was used in place of the amide of Gly.

^b^Compared to the corresponding distances in ligand‐free form structures, heatmap colorscheme applied in columns without numbers, where darker colors indicate larger difference in d1, d2, d3 distances between ligand‐free and ligand‐bound structures.

### 2.4 Evaluation of the role of Pro506 in the conformation of the oxyanion loop

**FIGURE 6:**
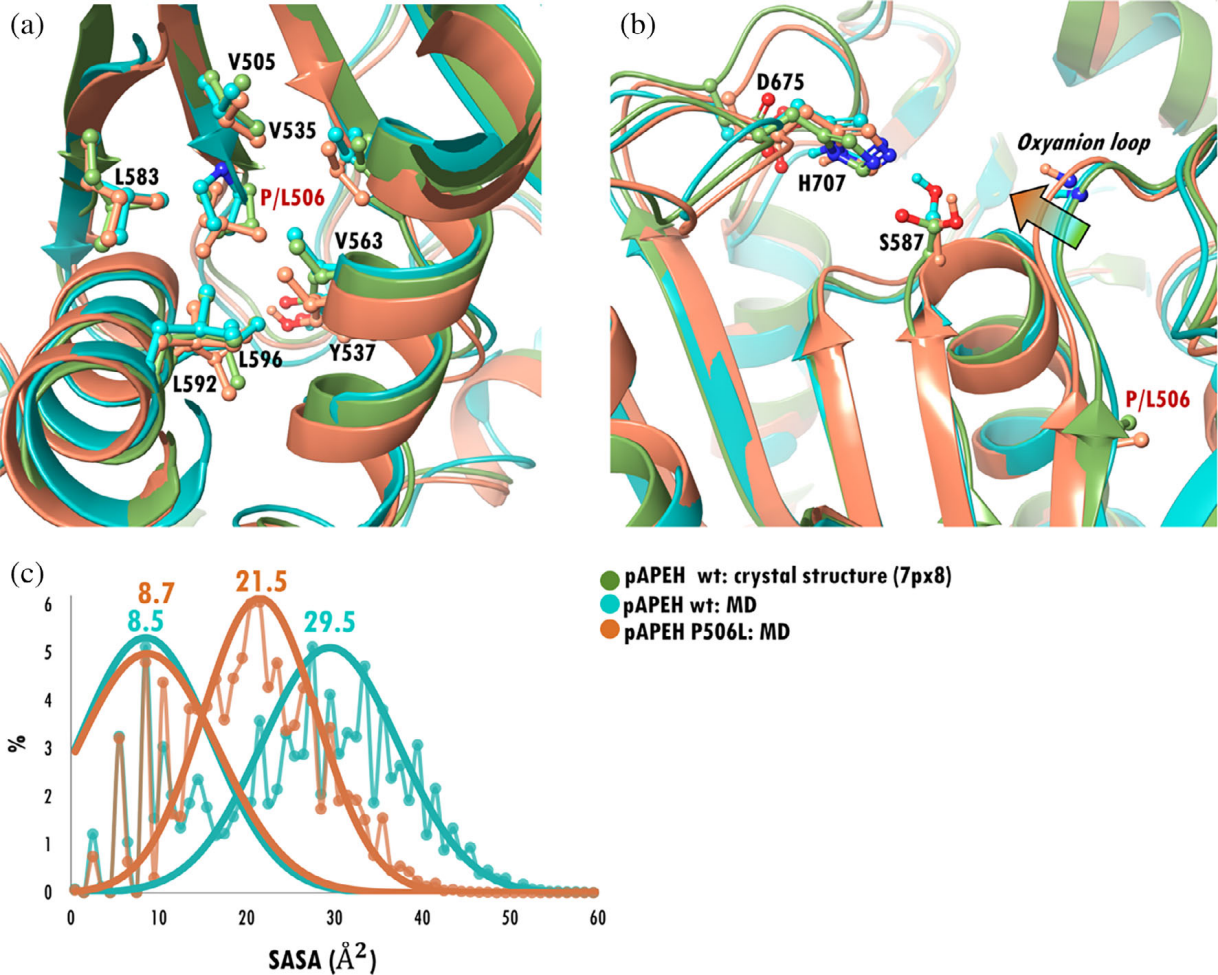
MD simulations of wt and P506L pAPEH. (a) The hydrophobic pocket around residue 506. Leu506 creates more favorable contacts here than Pro506 of the wt, contributing to the rigidification of the central core. (b) Differences seen at the active site: In case of the P506L pAPEH variant the oxyanion loop shifts forward, narrowing the catalytic pocket and obscuring the access to the catalytic Ser587. Representative structures (cluster mid‐structures) of the most populated clusters are shown. (c) The overall average of SASA decreases from 26 ± 11 Å^2^ to 20 ± 8 Å^2^ with the introduction of the P506L mutation. The distribution of SASA values measured during the last 100 ns of the trajectory is shown on the graph. The numbers above the peaks show the center of the fitted Gaussian curves with σ values of 0.5, 0.6, 0.6, AND 0.4 for maximum 1 and 2 of the wt and mutant systems, respectively.

We carried out in‐silico mutagenesis in combination with MD simulations to study the structural significance of Pro506. Two variants of tetrameric unligated pAPEH (as determined by cryoEM [7px8]) were studied: wt and P506L, to see whether the structure of the active site is truly influenced by the presence or absence of Pro506. Leu was chosen because it is often found in the corresponding position of α/β hydrolases. We found that the P506L mutation resulted in the forward shift of the oxyanion loop and a decrease in the solvent accessible surface area (SASA) of the side chain of the active Ser587. The distribution of SASA values is bimodal for both systems, corresponding to a similarly compact conformation with ~9 Å^2^ accessible surface and a more relaxed state, with 29.5 and 21.5 Å^2^ SASA for the wt and P506L pAPEH systems, respectively. (Figure 6) Results thus indicate that the presence of the strand‐breaker Pro in the central β‐sheet of the hydrolase domain contributes to the relaxation of the oxyanion loop and the coupled enlargement of the active site.

## 3 DISCUSSION

Enzyme reactions are often facilitated by structural rearrangements (Engel et al., 2006). The magnitude of these alterations varies from large subunit movements (ATP synthase [Lai et al., 2023], kinases [Reinhardt & Leonard, 2023], AND caspases [Riedl & Shi, 2004; Shi, 2004]) to small shifts of amino acid positions as in hemoglobin, where 0.4 Å displacement of the proximal His toward the heme iron results in an increased affinity for oxygen (Figure S7) (Eaton et al., 1999; Nagatomo et al., 2015).

In this study, the binding of the inhibitors to pAPEH showed some canonical, lock‐and‐key features as they utilized the shallow, rather hydrophobic S1 binding pocket to dock into the active site. S1 of pAPEH accommodates small polar groups such as Thr, Ala, Ser, or the flexible hydrophobic Met as its preferred sidechains (Krishna & Wold, 1992). Here we found that the Ala sidechain of AcAMHK and the methoxy group of DMP also dock into this pocket, as did the hydroxyethyl group of Meropenem (a carbapenem antibiotic studied in our previous study [Kiss‐Szemán, Takács, et al., 2022]), while other bulkier ligand‐substituents can fit into the large side opening of the monomer (Figure 6b).

Induced fit effects are also present (Figure 6a), involving repositioning of side chains of Arg677 (conserved as *H*‐bonding partner of the P2 residue of peptide substrates), Asp624 (its charge‐neutralizing pair). However, what truly seemed like a characteristic feature pertaining to pAPEH was that the binding of inhibitors also rearranges the position and conformation of its oxyanion‐ and catalytic His‐loops. The oxyanion‐loop shifted toward the inhibitor in all three cases while the His‐loop accommodated these changes. When binding the chloromethyl‐ketone inhibitor, the His‐loop of pAPEH moves forward since it has a chance of forming a covalent association with the reactive center, while in the case of bulky substituents, branching close to their anchor point (the active Ser) where no such covalent linkage is possible, the His‐loop shifts farther to accommodate the partner molecule. This adaptability was already recognized in the context of the binding of Meropenem to pAPEH, where the catalytic His‐loop shifted away from the active Ser while the His side chain flipped and was fixed by Asp214 in a decidedly un‐catalytic position (Kiss‐Szemán, Takács, et al., 2022) (Figure 6b).

The three complexes of pAPEH that we present here mimic the configuration of the first tetrahedral intermediate of the canonical peptide hydrolytic catalytic pathway of serine hydrolases, where two hydrogen bonds donated by the oxyanion site stabilize the negatively charged oxygen atom of the covalent enzyme/substrate adduct. It was thus quite surprising to find that the ligand‐binding geometry is suboptimal in the pAPEH‐inhibitor complexes studied here, lacking some of these critical *H*‐bonds (Figures 2a, 5a, 6a, and S6d). The pAPEH active site seems stretched to the limit, as evidenced by the calculations indicating that if the oxyanion loop were rigid, a water molecule would be wedged between the ligand and the oxyanion site (Figure 4b), considerably impairing the catalytic potential of the enzyme. The observed plasticity of the active site of mammalian APEH is possibly related to its biological role: operating with a highly adaptive and spacious, yet functional active site could potentiate it for the removal of oxidized, misfolded, or otherwise structurally “suspicious” compounds decorated with various substituents near the cleavage site. Among others, APEH is responsible for the clearance of proteins damaged by reactive oxygen species (ROS) (Hoernstein et al., 2023; Hoernstein et al., 2025; Nakai et al., 2012; Riccio et al., 2015; Shimizu et al., 2003; Shimizu et al., 2004; Tyler et al., 2021) as well as coordinating widely varied, unusual small‐molecular targets (see Data S1, chapter “Promiscuity of mammalian APEH”).

### 3.1 Physiological targets of organophosphates

There are two recognized targets of OPs, the inhibition of which leads to pathogenic conditions: acetylcholinesterase (AChE) and neuropathy target esterase (NTE) (Glynn, 1999). Inhibition of AChE causes over‐stimulation of acetylcholine receptors, resulting in muscle weakness, miosis, hypersalivation, sweating, or—in severe cases—respiratory failure, convulsions, and death, while interference with NTE leads to a condition called OP compound‐induced delayed neurotoxicity (OCIDN) that manifests in paralysis and ataxia a few weeks after the poisoning (Bouknight et al., 2020; Quistad & Casida, 2000). The inhibition of both AChE and NTE proceeds by the OP compounds phosphorylating the catalytic Ser residue of these serine hydrolases, first producing a phosphoester, similarly to that seen in the case of pAPEH in this study, followed by, in certain cases, a dealkylation step called aging (Hörnberg et al., 2007) that results in a non‐reactivatable enzyme complex, which ultimately leads to the formation of a negatively charged inhibitor moiety that is resistant to nucleophilic attack (Worek et al., 2004).

There are a number of further serine hydrolase enzymes—curiously mostly α/β hydrolases with a topologically similar fold of that of APEHs—that are similarly modified by OP compounds with so far uncharacterized physiological consequences. Of these, carboxylesterases (CES1, for example) and butyrylcholine esterase (BChE) are thought to be the primary endogenous bioscavengers of OPs. Both appear throughout the body and can coordinate a great variety of OPs without adverse physiological effects. In addition, platelet activating factor acetylhydrolase (PAF), cathepsin A and dipeptidyl peptidase 4 (DDP4) have also been shown to interact with OPs (Casida et al., 2008).

### 3.2 Size of the active site and substrate‐induced structural perturbations in APEH and other serine hydrolases

To see if the spaciousness and adaptability of the pAPEH active site is truly a unique feature among α/β hydrolases, we considered the ligand‐free and inhibitor‐bound forms of those mammalian (mostly human) members of the superfamily where structure determination was carried out for both. First, we focused on OP‐complexes.

**FIGURE 7:**
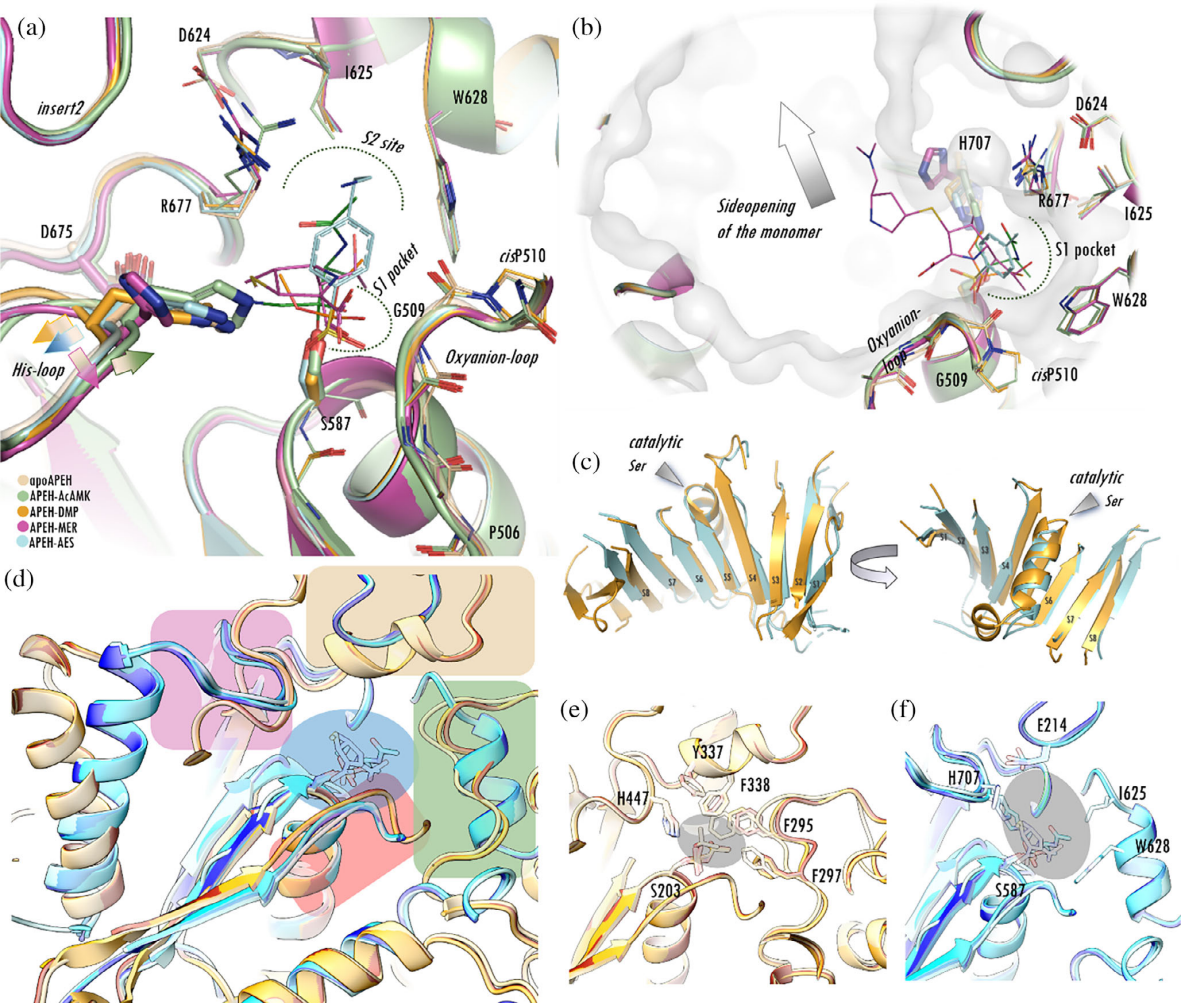
Comparison of ligand bound APEH and AChE structures. (a) Characteristic structural changes of pAPEH active site upon ligand binding: The oxyanion‐loop is shifted toward the coordinated oxygen atom of the ligand; the His‐loop flexibly follows the structural changes caused by ligand binding; and the catalytic Asp675 traces to the movement of the His‐loop (highlighted with color coded arrows). Conformational changes of *H*‐bonded pair of Arg677 and Asp624 involved in the S2 site are also shown. (b) The shallow rather hydrophobic S1 binding pocket is capable of accommodation of small or linear groups (Tyler et al., 2021). Bulkier substituents of the ligand reach beyond the S1 binding region into a large cavity leading to the side opening of the monomer. (c–f) Comparison of pAPEH and AChE, targets of phospholigands. (c) pAPEH (blue)and AChE (yellow) hydrolase domains share α/β hydrolase fold, shown with the central β‐sheets aligned. (d) Comparison of the active sites of phospholigand‐bound AChEs (PDB IDs 1j06: White, 2y2v: Yellow, 2jgm: Orange, 2jgk: Red, respectively) and APEHs (PDB IDs 7px8: Light blue, 9gne: Cyan, 9gou: Marine blue, 7qun: Dark blue). The covalently bound ligands (blue circle) occupy the same region. The active site of AChE is significantly more restricted with main structural elements being closer to the active Ser: The catalytic His‐loop (purple rectangle), oxyanion‐loops (red rectangle), the acyl‐pocket forming segment (green rectangle), and a mammalian specific flexible insertion APEHs (insert2 of pAPEH; in AchE there is a helix in this position, light brown rectangle). (e) Aromatic side chains lining the active site of AChE. Conformational change of Phe295 region makes it even more restricted during aging of organophosphorous ligands. (f) The active site of pAPEH is more spacious compared to AChE (highlighted in gray) with a more flexible His‐loop (as seen in meropenem bound structure PDB: 7qun).

The catalytic core structure of α/β hydrolases is conserved (Figure 7c): a β‐sheet forms its scaffold within which the catalytic serine is placed on a sharp turn (Ser‐loop) connecting a buried strand of this sheet and the helix that follows it. The catalytic His‐loop and the oxyanion‐loop can also be found in most serine hydrolases of this family—making the structural comparison straightforward. This is demonstrated on Figure 5, using the example of the pAPEH versus AChE comparison. It is immediately apparent that the His‐loop and the oxyanion‐loop are closer to the active Ser in the AChE structures (Figure 7d). The active site is further narrowed by a helix (residues 335–341) and the acyl‐pocket forming loop (residues 294–297) in the AChE structures, whilst in pAPEH structures a mammalian specific flexible insertion (*insert2*, residues 212–215) and the S1‐pocket forming helix (residues 625–632) create more space near the active site. When comparing the ligand‐free and OP‐inhibited forms of AChE, it is apparent that coordination of even bulky inhibitors is completed without structural rearrangement of the active site—therefore the ligand‐free form of AChE is already in a catalytically active conformation (Figures S8 and S9).

Comparison of the structures of human enzymes for which the structure of both the ligand‐free and OP‐inhibited forms are available with the pAPEH structures (which—sharing 98% sequence homology with the human enzyme—provide a sufficient model of it) showed that the pAPEH active site is the most spacious of them all. We measured three distances to characterize this spaciousness: the *Cα*(Ser*)*—Cα*(His*) (the asterisk denotes the members of the catalytic triad), *Cα*(Ser*)*—NH*(*oxyanion‐loop*) (selecting the heteroatom that coordinates the negatively charged phosphate oxygen of the substrate formed via the nucleophilic attack of the active Ser), and the NH(oxyanion‐loop)—*C*α(His*) distances (Tables 1 and S2: *d1*, *d2*, and *d3* distances, respectively). It can also be seen that pAPEH shows the largest changes in these dimensions upon OP coordination—brought about by the significant forward shift of the oxyanion‐loop. The two enzymes, beside pAPEH, that seem more adaptable than the rest (although possessing only a fraction of the spaciousness or pliability of pAPEH) are the two proposed detoxification serine hydrolases, BChE and CES1. While this relaxed and more open conformation of the ligand‐free form of pAPEH allows enough space for the docking of such unusual partners as carbapenem antibiotics or valproate‐glucuronide besides the OPs, it seems that the enlarged active site is not catalytically competent enough for processing them. Thus, when binding a substrate/inhibitor, the oxyanion‐loop moves forward to assist in the coordination of the negatively charged oxygen of the evolving complex. Interestingly, the single naturally occurring mutation of APEH, T541M, that can be suspected of reducing its catalytic activity (Kiss‐Szemán, Stráner, et al., 2022; Tsortouktzidis et al., 2019) by exerting a direct effect over the active site is located in the vicinity of the oxyanion‐loop, where it might contribute to the reshaping of the loop and restricting its free movement (all other naturally occurring mutations of APEH that cause loss or impairment of function are located on the tetramerization surfaces and most likely interfere with the self‐assembly of the tetramer form).

Similar rearrangements can be seen when pAPEH binds AcACMK. Since in this case a covalent bond is established both toward the catalytic Ser and the catalytic His, both the His‐loop and the oxyanion‐loop move closer to the inhibitor (and each other), tightening all three dimensions of the active site. However, no such shift is caused by the binding of CMK‐derivatives by the archaeal variants of the enzyme, as we have noted previously (Figure 3). In fact, both the size and the invariability of the active site of *Ap*APEH (or *Ap*AAP) and *Ph*APEH (or *Ph*AAP) are closer to those of the “regular” serine hydrolases than to those of pAPEH.

Serine proteases—proteolytic enzymes with a serine nucleophile—can be grouped into 13 clans, representing the various configurations of the catalytic apparatus and folds (Hedstrom, 2002; Jumper et al., 2021; Rawlings et al., 2018). Within these, besides the prolyl‐oligopeptidase family, of which APEH is also a member, three further distinct folds (those represented by trypsin, subtilisin, and ClpP peptidase) utilize a Ser‐His‐Asp triad to catalyze the hydrolysis of peptide bonds (Di Cera, 2009). Comparing the active site dimensions and variability of pAPEH to those of representative members of 10 clans (where appropriate experimentally determined structures were available) also demonstrates that pAPEH carries an unusually spacious and adaptive active site (Table S3): with both the distances of catalytic residues in the ligand‐free enzymes and their changes upon formation of tetrahedral intermediate‐like complexes being significantly larger in the case of pAPEH.

The composition of the active site is quite similar in the archaeal systems to that of pAPEH and human APEH, so much so that AlphaFold (Page & Di Cera, 2008; Varadi et al., 2022) predicts a similarly compact active site for the mammalian variants of the enzyme (Table 1), failing to anticipate the effect of the single striking difference between the archaeal and the mammalian forms: the presence of Pro506 near the active Ser at the stem of the oxyanion‐loop (in both the porcine and the human variants) (Figures S10 and S12). The presence of Pro506 shortens the 4th β‐strand of the core β‐sheet of pAPEH, allowing freer movement to both the oxyanion‐loop and the Ser‐loop that is supported by the neighboring 5th strand. We previously found that this relaxation of the tightly wound Ser‐loop is responsible for the partial inactivation of the ligand free‐form, which alternates between latent and active conformations, as a result (Kiss‐Szemán, Stráner, et al., 2022). Here, we established that this same Pro is also the most likely inducer of the backward shift and flexibility of the oxyanion‐loop, and also demonstrated that archaeal variants that carry Val at this position do not show the same promiscuity (see Data S1, Chapter II: Substrate/inhibitor preference of APEH from *Aeropyrum pernix* and *Pyrococcus horikoshii*). In fact, the majority of non‐eukaryotic APEHs do not carry a Pro in this position, and neither do most of the S9 serine protease family enzymes, nor any of the human α/β hydrolases sampled here for comparison (see Figures S10–S12 and Tables S2 and S3).

## 4 CONCLUSION

Due to its diverse functions, APEH has been indicated as a possible pharmacological target in various forms of cancer (Bergamo et al., 2013; Erlandsson et al., 1991; Gogliettino et al., 2021; McGoldrick et al., 2014; Naylor et al., 1989; Palumbo et al., 2016; Tangri et al., 2021) and as a biomarker of multiple organ injuries (Liu et al., 2025). The high affinity of APEH toward OPs has suggested that it could also be used as a biomonitor of OP exposure (Huang et al., 2025)—of an agricultural worker, for example—even if weeks have passed following the event, by determining the fraction of OP‐inhibited APEH content of red blood cells (Ramírez‐Santana et al., 2018). On the other hand, surprisingly, through this very same sensitivity toward the OPs, APEH has also become a potential target for cognitive enhancing drugs promoting synaptic plasticity and learning in young brains (García‐Rojo et al., 2017; Sandoval et al., 2012). Both developing sensitive and specific monitoring systems and attempting to design nontoxic but effective inhibitors of APEH require a precise understanding of the mode and requirements of the binding process—the structure of the pAPEH‐OP complex presented here can be utilized toward these goals. The structure of the AcAMHK complex also holds physiological significance: it was proposed that chloromethyl ketone derivatives of *N*‐acetylated peptides and their phosphonic analogues induce apoptosis in cancer cell lines through inhibition of APEH (Walczak et al., 2021; Yamaguchi et al., 1999).

Serine hydrolyses can be categorized according to various criteria. Their mechanism of action is mostly the same: a catalytic triad consisting of a Ser, His, and Asp residues cooperatively cleaves a covalent bond. The real difference lies in the wide variability of their biological function, encoded in their substrate selectivity and specificity. Mammalian APEH is a versatile detoxifier carrying the same fold and catalytic apparatus as some very specific α/β hydrolases. Here we found that pAPEH differs from canonical serine hydrolases by “dimension‐wise” very small differences, on the scale of 0.5–2.5 Å. However, these small but consistent differences are the only telltale signs of its different functionality. A Pro residue present in the central β‐sheet of the α/β hydrolase domain (Pro506) causes a break of a β‐strand, and through this, a general loosening of the active site, complemented by more widespread, but still characteristic features such as its shallow primary substrate recognition pocket (S1), and a *cis*Pro (Pro510) that acts as a rigidifier of the oxyanion loop. We propose that these differences together shape the easily approachable active site of APEH with its reactivatable catalytic machinery, turning mammalian APEH into a potent, all‐purpose degrading enzyme. The adaptability of the active site is essential for detoxification since this function entails elimination of oxidized, misfolded, or otherwise structurally impaired oligopeptides and proteins that appear in necessarily unpredictable shapes and sizes. The example of APEH demonstrates why evolution might favor the upkeep of promiscuity rather than optimizing an enzyme toward specificity, although it cannot be determined with certainty whether the appearance or disappearance of a functionally distinctive natural mutation such as Pro506 in the present case is an evolutionary response (a feature) or just a coincidence (or a bug) that has evolved into a true function. Either way, the structurally modest but catalytically significant variations produced by the appearance of Pro506 in mammalian APEH can be viewed as the result of the natural‐intelligence design process where the composition and topology of the catalytic apparatus, the Ser‐His‐Asp triad in this case, cannot be significantly altered without losing catalytic efficiency, but selectivity can nonetheless be optimized—similar to the steps taken very recently in an AI‐based design campaign to create and optimize artificial counterparts of these serine hydrolase enzymes (Bonollo et al., 2025; Lauko et al., 2025).

## 5 MATERIALS AND METHODS

### 5.1 Purification of porcine liver APEH and inhibition with AcACMK (ligand1) and DDCV (ligand2)

The preparation and purification of the mammalian APEH sample (from porcine liver) was carried out as in our previous study (Kiss‐Szemán, Stráner, et al., 2022). Tetrameric composition was verified by size exclusion chromatography. To monitor that the catalytically competent form of the enzyme was preserved during the purification process, concentrated samples of pAPEH were incubated with *N*‐acetyl‐alanine *p*‐nitroanilide (Ac‐Ala‐pNA, AANA, eNovation Chemicals LLC) as a substrate (Jones et al., 1994) (1.6 μM in 5% DMF/water) in buffer (50 mM phosphate, pH = 8, 0.3 M NaCl, 1 mM EDTA, 5 mM mercaptoethanol) at 37°C (reaction mixture: 10 μL of AANA solution, 985 μL buffer, 5 μL protein sample). The formation of *p*‐nitroaniline was measured spectrophotometrically by monitoring the increase in absorbance at 410 nm.

APEH purified from porcine liver (1 mg/mL, 0.01 mM solution in 10 mM TRIS buffer, pH = 7.5) mixed with ligands (*ligand 1*: acetyl‐alanyl‐chloromethylketone, AcAlaCMK, synthesized in house (Figure S13). Briefly: acetyl‐alanine was reacted with isobutylchloroformate and the resulting mixed anhydride was treated with diazomethane; then the diazomethylketone was treated with HCl to yield the acetyl‐alanyl‐chloromethylketone product (Figure S13). *Ligand 2*: dichlorvos, DDCV, purchased from Merck, Pestanal, analytical standard; *ligand 3*: AEBSF, 4‐(2‐aminoethyl)benzenesulfonyl fluoride, purchased from Merck). All compounds are >95% pure by HPLC. Ligands were added in 10‐fold molar excess dissolved in sample buffer; the samples were incubated at 37°C (total reaction volume: 100 μL). After 1 h of incubation, the protein sample was washed with 10 mL of sample buffer to wash away the unbound ligands, using ultrafilter units (Amicon, MWCO 30 kDa). After adjusting the protein concentration to the original value, there was no measurable specific activity. The samples were frozen in N_2_(liq.) and stored at −80°C.

### 5.2 CryoEM sample preparation and data acquisition

*APEH—Ligand 1 complex*: Purified protein sample (2 μL) of 0.5 mg ml^−1^ concentration in 10 mM TRIS (pH = 7.5) buffer was placed on a Quantifoil R1.2/1.3 grid (Cu 400 mesh, glow discharged for hydrophilization with 20 mA, for 60 s by GloQube Quorum) and was vitrified. After 2 s of blotting time, the grid (4°C, 90% humidity) was plunge‐frozen in liquid ethane (Leica EMGP2). CryoEM single particle data collection was performed using a CRYOARM 300II microscope operated at 300 kV equipped with a K3 camera (Gatan). Images were recorded at 80,000‐fold magnification corresponding to 0.5115 Å per pixel using a 20 eV energy filter (Omega Filter) with an exposure time of 4 s and a total electron dose of 40 e/Å^2^. Spherical aberration coefficient (Cs) was 2.7 mm and defocus range 0.5–2.5 μm. A total of 8604 micrographs were collected from a single grid (Figures S1 and S2 and Table S1).

*APEH*—*Ligand 2 complex*: Purified protein sample (3.5 μL) of 0.5 mg ml^−1^ concentration in 10 mM TRIS (pH = 7.5) buffer was placed on a Quantifoil R2/2 grid (Cu 300 mesh, glow discharged beforehand). Sample was blotted (wait time 30s, blot time 4 s, blot force +2, with no drain time, at 4°C and 95% chamber humidity) and was vitrified in liquid ethane (Vitrobot MkIV). CryoEM single particle data collection was performed using a KRIOS G4 microscope operated at 300 kV equipped with a EF—Falcon 4i camera. Images were recorded at 165,000‐fold magnification corresponding to 0.75 Å per pixel using a 10 eV slit with exposure time of 1.9 s and a total electron dose of 11.9 e/Å^2^. Spherical aberration coefficient (Cs) was 2.7 mm and defocus range−1.5 − (−0.5) μm. A total of 12,467 micrographs were collected from a single grid (Figures S3, S4, and Table S1).

*APEH—Ligand 3 complex*: Purified protein sample (3.5 μL) of 0.5 mg ml^−1^ concentration in 10 mM TRIS (pH = 7.5) buffer was placed on a Quantifoil R2/2 grid (Cu 300 mesh, glow discharged beforehand). Sample was blotted (wait time 30 s, blot time 4 s, blot force +2, with no drain time, at 4°C and 95% chamber humidity) and was vitrified in liquid ethane (Vitrobot MkIV). CryoEM single particle data collection was performed using a KRIOS G4 microscope operated at 300 kV equipped with a EF—Falcon 4i camera. Images were recorded at 165,000‐fold magnification corresponding to 0.75 Å per pixel using a 10 eV slit with exposure time of 1.9 s and a total electron dose of 11.9 e/Å^2^. Spherical aberration coefficient (Cs) was 2.7 mm and defocus range −1.5 − (−0.5) μm. A total of 4506 micrographs were collected from a single grid (Figures S5 and S6 and Table S1).

### 5.3 Cryo‐EM data processing

Movies were subjected to beam‐induced motion correction using CryoSPARC (Punjani et al., 2017) and contrast transfer function parameters were estimated by CTFFIND4 (Rohou & Grigorieff, 2015). All of the following processes were performed using CryoSPARC. Particles were auto‐picked, and two rounds of reference‐free two‐dimensional (2D) classification were performed.

APEH‐Ligand 1: In total, 2,151,591 particles from 8604 micrographs were auto‐picked and subjected to 2D classification, where eight classes were selected with 412,960 particles that were used for ab initio reconstruction. 3D refinement was performed applying C1 and D2 symmetry. Final 3D refinement and postprocessing yielded a map with an overall resolution of 3.48 Å (C1 symmetry) and 3.19 Å (D2 symmetry), estimated by the gold‐standard FSC = 0.143 criterion (Figure S1).

APEH‐Ligand 2: In total, 1,433,246 particles from 12,467 micrographs were auto‐picked and subjected to the 2D classification, where eight classes were selected with 141,000 particles that were used for 3D Ab initio reconstruction. 3D refinement was performed applying C1 and D2 symmetry. Final 3D refinement and postprocessing yielded a map with an overall resolution of 2.99 Å (C1 symmetry) and 2.65 Å (D2 symmetry), estimated by the gold‐standard FSC = 0.143 criterion (Figure S3).

APEH‐Ligand 3: In total, 2,445,076 particles from 4506 micrographs were auto‐picked and subjected to the 2D classification, where 10 classes were selected with 169,418 particles that were used for 3D Ab initio reconstruction. 3D refinement was performed applying C1 and D2 symmetry. Final 3D refinement and postprocessing yielded a map with an overall resolution of 2.98 Å (C1 symmetry) and 2.78 Å (D2 symmetry), estimated by the gold‐standard FSC = 0.143 criterion (Figure S5).

### 5.4 Model building and refinement

Model building was carried out for all the three experiments by docking the previously determined structure of the uncomplexed APEH (PDB id: 7px8), using Phenix Dock in Map (Liebschner et al., 2019). Manual finishing of the tetramer was carried out in Coot (Emsley & Cowtan, 2004) and the structures were refined with real space refinement using Phenix (Afonine et al., 2018).

### 5.5 Validation and visualization

Refined structures were validated in Phenix (Figures S2, S4, and S6 and Table S1). Local resolution estimation of cryoEM maps was calculated with Phenix (Liebschner et al., 2019; Figures 1b and 2b). Figures were generated using PyMOL (https://pymol.org), Maestro (Schrödinger Suite [Schrödinger Release 2019‐3: Maestro, Schrödinger, LLC, New York, NY, 2019]), and UCSF ChimeraX (Pettersen et al., 2021). Comparison and geometrical analysis of the structures were carried out using Maestro, superimposing the core structures of the hydrolase domains of the studied enzymes: the backbone atoms of the eight β‐strands of the central β‐sheet and the helix following the catalytic Ser.

### 5.6 Calculations

The starting conformations for the QM/MM calculations were derived from a molecular dynamics (MD) trajectory of a hybrid model generated using the ligand‐free form of pAPEH (based on PDB structure 7px8^7^), modeling missing segments using Maestro (Schrödinger Suite [Schrödinger Release 2019‐3: Maestro, Schrödinger, LLC, New York, NY, 2019]) with the AcAMK inhibitor inserted in the conformation determined by cryo‐EM in the present study. A 100 ns NPT simulation was carried out at 310 K and 1 bar using GROMACS and the AMBER‐ff99SBildnp* force field (Pronk et al., 2013). The system was solvated with OPC water molecules (Izadi et al., 2014) in a dodecahedral box. Total charge was neutralized and physiological salt concentration (0.15 M) was set using Na^+^ and Cl^−^ ions. Energy minimization of starting structures was followed by sequential relaxation of constraints on protein atoms in three steps and an additional NVT step (100 ps) to stabilize pressure, but a 100 kJ/mol*nm^2^ restraint was maintained for the backbone atoms of the 9–732 segment of each chain. Restraints had to be introduced to derive starting models that would afford comparable QM/MM energies representing only the effect of the modest shift of the oxyanion loop and filter out the excessive conformational heterogeneity of the gatekeeper segments (res. 110–115 and 182–192) that we encountered in a previous study (Kiss‐Szemán, Stráner, et al., 2022). After clustering all appearing monomer conformations (4 in the tetrameric systems of each snapshot) based on the position of the main‐chain and Cβ atoms (with a 1 Å cutoff) over the 50–100 ns segment of the trajectory, two representative conformations were chosen for QM/MM analysis: one where a direct *H*‐bond is formed between the inhibitor and the backbone carbonyl of Gly509 of the oxyanion loop and one where the loop remains in the relaxed conformation of the ligand‐free form and a water is inserted between the two (Figure S14). QM/MM calculations were carried out using Jaguar (Bochevarov et al., 2013; Schrödinger LLC, 2019). B3LYP/6‐31G** method in combination with OPLS3‐based molecular mechanics (Harder et al., 2016). QM region (132/135 atoms) included the entire Ser587 and His707 residues (capped by the preceding and following residues), the sidechains of Asp675 and Arg677 (capped by their respective main‐chain atoms), the Ac‐Ala‐CMK inhibitor in its covalently bound form and one/two water molecule(s). The MM region contained an entire monomer of pAPEH (and all waters and ions within 6 Å of it), but only the residues and waters within 9 Å of the catalytic triad were free to move.

To study the P506L mutation of pAPEH, we used the same starting structure built based on structure 7px8^7^. The mutation was introduced into the unchanged matrix of the wt form. MD was carried out as described above, but in this case no restraints were applied. The last 100 ns of 250 ns long production runs were evaluated. The trajectories of the tetrameric forms were dissected into monomeric units in this case too, fitted to each‐other, and this “monomer‐trajectory” was analyzed. Solvent accessible surface of the sidechain of the catalytic Ser was calculated, and the snapshots were clustered based on the position of the main‐chain and Cβ atoms (with a 1 Å cutoff) using GROMACS.

Modeling of the pAPEH‐POM‐ERJ complex was carried out using the Monte Carlo Multiple Minimum (MCMM) conformational search method as implemented in the Schrödinger Suite (Bochevarov et al., 2013). Starting model was built using the pAPEH—dimethoxy‐phosphate complex determined in this study. MC steps involved the random variation (within the range of 0–180°) of a randomly selected subset of all torsional angles of POM‐ERJ and random translations (within the range of 0–3 Å) and rigid‐body rotation (between 0 and 180°) of the ligand with respect to pAPEH. The perturbed structures were minimized using the Polack‐Ribièr conjugate gradient algorithm. Unique structures within a 21 kJ/mol energy window above the global minimum were stored. Calculations were carried out using the OPLS3 force field (Harder et al., 2016), allowing the relaxation of all protein sidechains reaching within 6 Å of the ligand. Solvent effects were modeled by the GB/SA algorithm (using water as solvent).

## AUTHOR CONTRIBUTIONS

**Anna J. Kiss‐Szemán:** Conceptualization; data curation; writing – original draft; writing – review and editing; validation; methodology; investigation; visualization; software; formal analysis; supervision; project administration. **Luca Takács:** Investigation. **Imre Jákli:** Software; data curation. **Zoltán Bánóczi:** Investigation. **Naoki Hosogi:** Data curation. **Daouda A. K. Traore:** Data curation. **Veronika Harmat:** Conceptualization; methodology; writing – review and editing. **András Perczel:** Conceptualization; funding acquisition; resources; supervision; writing – review and editing; project administration. **Dóra K. Menyhárd:** Conceptualization; writing – original draft; writing – review and editing; supervision; methodology; software; formal analysis; investigation; visualization; validation; data curation.

## CONFLICT OF INTEREST STATEMENT

The authors declare no competing interests.

## Supporting information


**FIGURE S1.** Workflow of cryo‐EM data processing for APEH‐AcAMHK (EMD‐51464, 9GNE).
**FIGURE S2.** Validation of the cryo‐EM data processing for APEH‐AcAMHK (EMD‐51464, 9GNE).
**FIGURE S3.** Workflow of cryo‐EM data processing for APEH‐DMP (EMD‐51501, 9GOU).
**FIGURE S4.** Validation of the cryo‐EM data processing for APEH‐DMP (EMD‐51501, 9GOU).
**FIGURE S5.** Workflow of cryo‐EM data processing for APEH‐AES (EMD‐52489, 9HXQ).
**FIGURE S6.** Validation of the cryo‐EM data processing of APEH‐AES (EMD‐52489, 9HXQ) and structure evaluation.
**TABLE S1.** Cryo‐EM data collection, refinement and validation statistics for APEH‐AcAMHK, APEH‐DMP and APEH‐AES.
**FIGURE S7.** The shift resulting in a conformational change that increases the affinity for oxygen (cooperative binding) in hemoglobin. Upon binding oxygen, a shift in the heme group's structure occurs, resulting in a slight displacement (0.4 Å) of the iron atom and a change in the position of neighboring amino acids. Unbound structure shown in cyan (PDB id: 2hbs) and the oxygen‐bound structure in yellow (PDB id: 6bb5). The shift of 0.4 Å is measured at the distance between the iron‐ion of the heme and His92 Nε2 atoms.
**FIGURE S8.** The binding of even bulky inhibitors to AChE is completed without structural rearrangement of the active site. The structures of (a) apoAPEH (PDBid 7px8) and apoAChE (1j06) showing the catalytic Ser and His residues aligning well (gray circle). The oxyanion loop is longer and in closer proximity to the catalytic Ser in AChE (blue rectangle). The binding pocket forming backbone (also called aging loop in AChE, yellow box) and the mammalian specific flexible insert in APEH (red box) create more space around the active site. (b) apoAChE (PDB id: 1j06) and ligand bound structures with VX (PDB id: 2y2u), methamidophos (MeP, PDB id: 2jge) and fenamiphos (FeP, PDB id:2jgf), (c) apoAChE (PDB id: 1j06), AChE‐DFP (PDB id: 2jgi) and AChE with aged DFP (PDB id: 2jgm): the *aging loop* is moved upon DFP binding and does not change with aging. (d) apoAChE (1j06), AChE‐VX (2jgh) and AChE with aged VX (2jgl), (e) apoAChE (1j06), AChE‐MeP (2jge) and AChE with aged MeP (2jgj), (f) apoAChE (1j06), AChE‐FeP (2jgf) and AChE with aged FeP (2jgk): the *aging loop* is shifted upon aging.
**FIGURE S9.** Organophosphorous compounds binding to APEH and acetylcholinesterase (AChE). Leaving groups, indicated by the red circles, are the substituents of the ligand that dissociate in the first reaction step of the serine hydrolase reaction. Substituents that are accommodated by the primary binding pocket (S1 for APEH and acyl‐pocket for AChE) are also the groups that take part in the aging reactions (green dotted rectangle). The OPs are arranged by decreasing IC_50_ values measured for APEH. The oxon (P=O bond) forms of OPs—created during natural metabolic processing from the corresponding thion –compounds (P=S bond)—are more susceptible toward the nucleophilic attack of the serine‐hydrolase enzymes, thus more reactive and more effective in inhibition of the enzymes (Hoernstein et al., 2023; Shimizu et al., 2004). The remaining substituents succumb to the aging process where the negatively charged phosphate is created, leaving the enzyme permanently inhibited, however, this process is modulated by the surrounding sidechains (Sandomenico et al., 2021). OPs with more hydrophobic leaving groups bind more effectively to AChE, because of the aromatic sidechains (Phe295, 297, 338, Tyr337, 341) in the proximity of the binding site.
**TABLE S2.** Characteristic distances of the active site of apo‐, organophosphate‐bound, and CMK‐derivatized serine hydrolases of the α/β hydrolase superfamily. Neither of these enzymes carry Pro in the position corresponding to Pro506 of pAPEH.
**FIGURE S10.** The phylogenetic tree of S9 enzyme family created by MEROPS database (Atkins, 2020; Honaker et al., 2011). The green box highlights the S9C subfamily (of which APEHs are members), the yellow box indicates the S9C subfamily entries that contain a *Pro* residue corresponding to Pro506 of pAPEH (on the 4th strand of the hydrolase domain core β‐sheet) and mammalian enzymes with this particular *Pro* mutation are marked with the blue box. (Light brown rectangles show the exceptions to this mutation.) Note: The MEROPS database contains seprate entries of the same organism with incomplete sequences.
**FIGURE S11.** The phylogenetic tree of the human APEH gene aligned with Ensembl (Mishra et al., 2022). The alignement shows that the APEH gene is present in all domains of life. For a detailed view visit: https://www.ensembl.org/Homo_sapiens/Gene/Compara_Tree?db=core;g=ENSG00000164062;r=3:49674014-49683971.
**FIGURE S12.** Phylogenetic tree of human APEH, gene alignment and the amino acids at the Pro506 site (pAPEH numbering) and the catalytic Ser. The gene and protein alignment was conducted with Panther (Nagai et al., 1997) and visualized with iTol (Mori et al., 2007) and WebLogo (Deshayes et al., 2017). The logos show the site of the strand‐breaker Pro of pAPEH and the following residues (blue outline) and the immediate surroundings of the catalyitic Ser (light blue outline). In the logos, orange triangles indicate the residues found at the position of Pro506, purple stars mark the glycine residue of the oxyanion‐site (if present) and red asterisks mark the catalytic serine residues. (Interestingly *Ap*APEH and *Ph*APEH are not present in this tree; this might be due to low sequence homology as Panther uses Hidden Markov Models (HMMs) with a cutoff criteria of 30% sequence homology).
**TABLE S3.** Characteristic distances of the active site of apo‐, OP‐, phosphonate‐ and CMK‐derivatized serine proteases of the serine protease clans (Dreucean et al., 2019).
**FIGURE S13.** Characterization of acetyl‐alanyl‐chloromethylketone (Ac‐Ala‐CMK). RP‐HPLC, Zorbax C18 5 μM, 150 × 4.6 mm, 300 Å pore size. Gradient: 0 min 0% B, 2 min. 0% B, 22 min. 90% B (eluent A: 0.1% TFA/water, eluent B: 20% 0.1%TFA/water and 80% acetonitrile). Flow 1 mL/min. *R*_*t*_ = 11,0 min. (ESI‐MS Bruker Esquire 3000plus, *M*_measured_ = 163 g/mol).
**FIGURE S14.** Representative structures of the five clusters (cluster centers) obtained during the short, restrained MD simulation of the pAPEH‐AcAMK complex intended to provide starting structures for the subsequent QM/MM calculations. Dark gray and maroon show the conformer where direct H‐bond is formed between the oxyanion loop and the inhibitor (35.1% of the snapshots belong to this cluster) and lighter gray and pink colors indicate those arrangements (4 clusters) where a more relaxed conformation of the oxyanion loop was found. The cryo‐EM structure of ligand‐free pAPEH (PDB: 7px8) is shown in green.

## Data Availability

Cryo‐EM map and atomic model have been deposited in the Electron Microscopy Data Bank (EMDB) and PDB, respectively, with the following accession codes APEH‐AcAMHK: EMD‐51464, 9GNE, APEH‐DMP: EMD‐51501, 9GOU, and APEH‐AES: EMD‐52489, 9HXQ. The crystal structure of *Ap*APEH from co‐crystallizing attempts with meropenem has been deposited with the PDB under accession code 9S6B. Authors will release the atomic coordinates and experimental data upon article publication.
