# Supplementary material for: Ligand binding Pro‐miscuity of acylpeptide hydrolase, structural analysis of a detoxifying serine hydrolase
Source: Protein Sci. 2025 Oct 11;34(11):e70320. doi: 10.1002/pro.70320 (PMC12514842; doi:10.1002/pro.70320)
Supplement: Supplementary file 1 — FIGURE S1. Workflow of cryo‐EM data processing for APEH‐AcAMHK (EMD‐51464, 9GNE). FIGURE S2. Validation of the cryo‐EM data processing for APEH‐AcAMHK (EMD‐51464, 9GNE). FIGURE S3. Workflow of cryo‐EM data processing for APEH‐DMP (EMD‐51501, 9GOU). FIGURE S4. Validation of the cryo‐EM data processing for APEH‐DMP (EMD‐51501, 9GOU). FIGURE S5. Workflow of cryo‐EM data processing for APEH‐AES (EMD‐52489, 9HXQ). FIGURE S6. Validation of the cryo‐EM data processing of APEH‐AES (EMD‐52489, 9HXQ) and structure evaluation. TABLE S1. Cryo‐EM data collection, refinement and validation statistics for APEH‐AcAMHK, APEH‐DMP and APEH‐AES. FIGURE S7. The shift resulting in a conformational change that increases the affinity for oxygen (cooperative binding) in hemoglobin. Upon binding oxygen, a shift in the heme group's structure occurs, resulting in a slight displacement (0.4 Å) of the iron atom and a change in the position of neighboring amino acids. Unbound structure shown in cyan (PDB id: 2hbs) and the oxygen‐bound structure in yellow (PDB id: 6bb5). The shift of 0.4 Å is measured at the distance between the iron‐ion of the heme and His92 Nε2 atoms. FIGURE S8. The binding of even bulky inhibitors to AChE is completed without structural rearrangement of the active site. The structures of (a) apoAPEH (PDBid 7px8) and apoAChE (1j06) showing the catalytic Ser and His residues aligning well (gray circle). The oxyanion loop is longer and in closer proximity to the catalytic Ser in AChE (blue rectangle). The binding pocket forming backbone (also called aging loop in AChE, yellow box) and the mammalian specific flexible insert in APEH (red box) create more space around the active site. (b) apoAChE (PDB id: 1j06) and ligand bound structures with VX (PDB id: 2y2u), methamidophos (MeP, PDB id: 2jge) and fenamiphos (FeP, PDB id:2jgf), (c) apoAChE (PDB id: 1j06), AChE‐DFP (PDB id: 2jgi) and AChE with aged DFP (PDB id: 2jgm): the aging loop is moved upon DFP binding and does not change with [file PRO-34-e70320-s001.docx]

**Supplementary Information for**

**Ligand binding *Pro*-miscuity of acylpeptide hydrolase,**

**structural analysis of a detoxifying serine hydrolase**

Anna J. Kiss-Szemán,^1^ Luca Takács,^1^ Imre Jákli,^1,2^ Zoltán Bánóczi,^3,4^ Naoki Hosogi,^5^ Daouda A.K. Traore,^6^

Veronika Harmat,^1,2^ András Perczel^1,2,7*^, Dóra K. Menyhárd^2,7*^

^1^ Laboratory of Structural Chemistry and Biology, Institute of Chemistry, Eötvös Loránd University, Budapest, Hungary.

^2^ HUN-REN – ELTE Protein Modelling Research Group, Budapest, Hungary.

^3^ Department of Organic Chemistry, Institute of Chemistry, ELTE Eötvös Loránd University, 1117, Budapest, Hungary.

^4^ HUN-REN – ELTE Research Group of Peptide Chemistry, 1117, Budapest, Hungary.

^5^ Electron Microscopy Application Department, EM Business Unit, JEOL Ltd, 3-1-2 Musashino Akishima, Tokyo 196-8556, Japan.

^6^ Materials and Structural Analysis Division, Thermo Fisher Scientific, Achtseweg Noord 5, 5651 GG, Eindhoven, The Netherlands

^7^ Medicinal Chemistry Research Group, HUN-REN Research Centre for Natural Sciences, Magyar Tudósok Körútja 2, H-1117 Budapest, Hungary

Corresponding authors: Dóra K. Menyhárd and András Perczel

*e-mail: dora.k.menyhard@ttk.elte.hu; perczel.andras@ttk.elte.hu

This file includes:

SI Chapter I: Promiscuity of mammalian APEH

SI Chapter II: Substrate/inhibitor preference of APEH from *Aeropyrum pernix* and *Pyrococcus horikoshii*

Figures S1-S14

Tables S1-S3

**SI Chapter I:** **Promiscuity of mammalian APEH**

We attempted to ascertain whether the ligand profile of APEH can be thought of as unusual, or promiscuous among human serine hydrolases. We have focused on three unexpected but confirmed partners of the enzyme: VPA-G (see Refs. 20-25 of Main Text)^MT20-25^, POM-ERJ, a lipophilic ester of a phosphonic acid antibiotic^MT27^ and the most widely used carbapenems (that were also shown to inhibit APEH).^MT8^

In a previous work^MT8^ we have carried out docking of VPA-G and unhydrolyzed meropenem into the active site pocket of unligated pAPEH (model built based on 7px8) and here we carried out conformational search for the binding of POM-ERJ also. In these pre-reaction complexes all three ligands bound in the vicinity of the catalytic Ser and also docked a sidechain into the S1 pocket. The arrangements clearly reflect a possibility of covalent attack by the catalytic Ser.


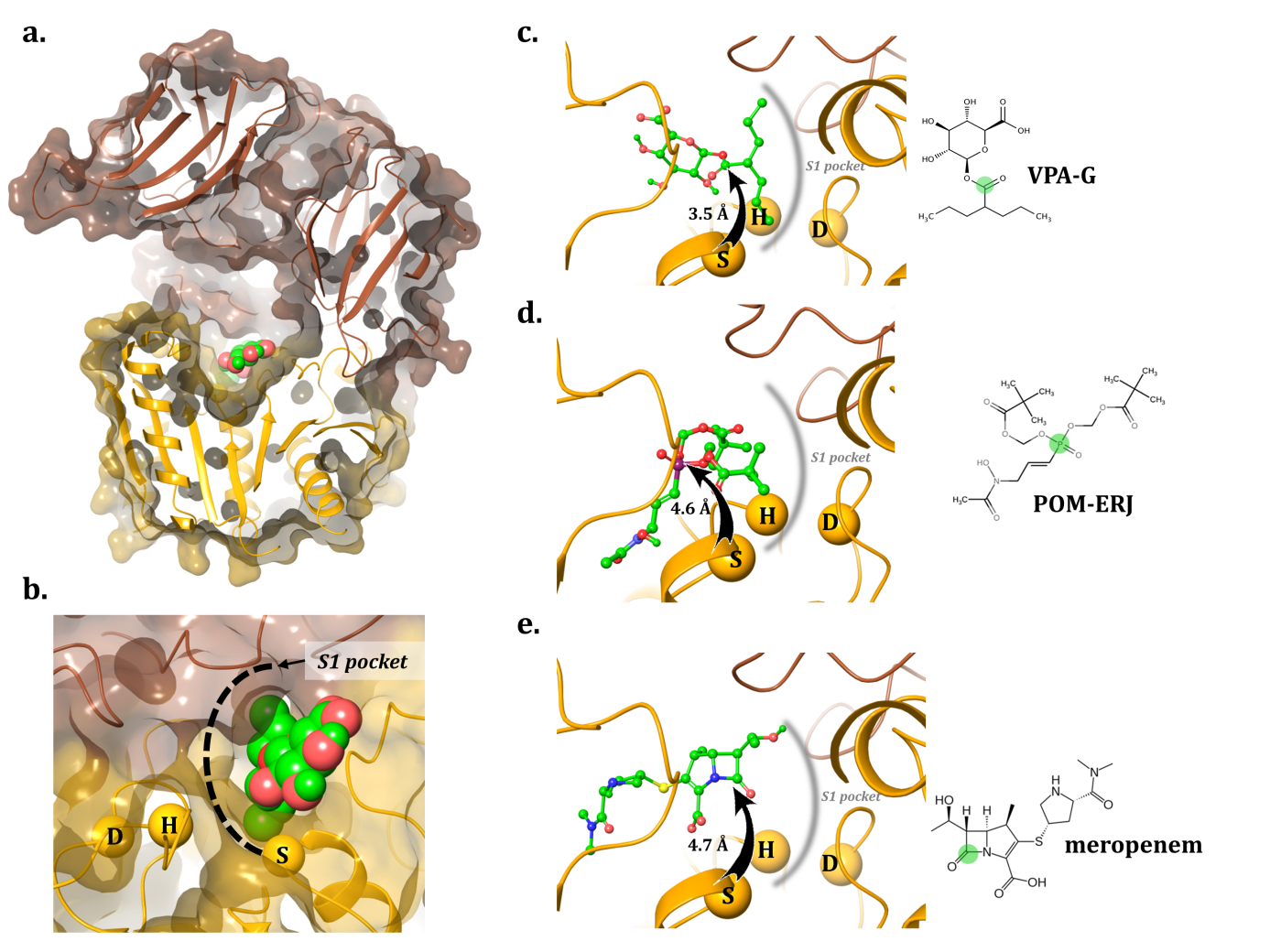


**Binding mode of selected ligands to pAPEH – as calculated by MCMM docking. a.)** VPA-G (green) docked into the active pocket between the propeller (brown) and hydrolase (yellow) domains of pAPEH. **b.)** Close-up of the active site, with the S1 pocket also shown. **c.-e.)** Active-site arrangement and the distance between the OG atom of Ser and the attack site of the ligands (indicated by the green dot on the schematic representation) in case of VPA-G, POM-ERJ and meropenem, respectively. The residues of the catalytic triad are represented by the spheres placed on their Cα atoms.

To find possible other targets of these ligands, first we checked the PubChem Database^1^ for known protein targets but found no serine hydrolases (aside for APEH) among the very few human enzymes indicated (highlighted in grey):


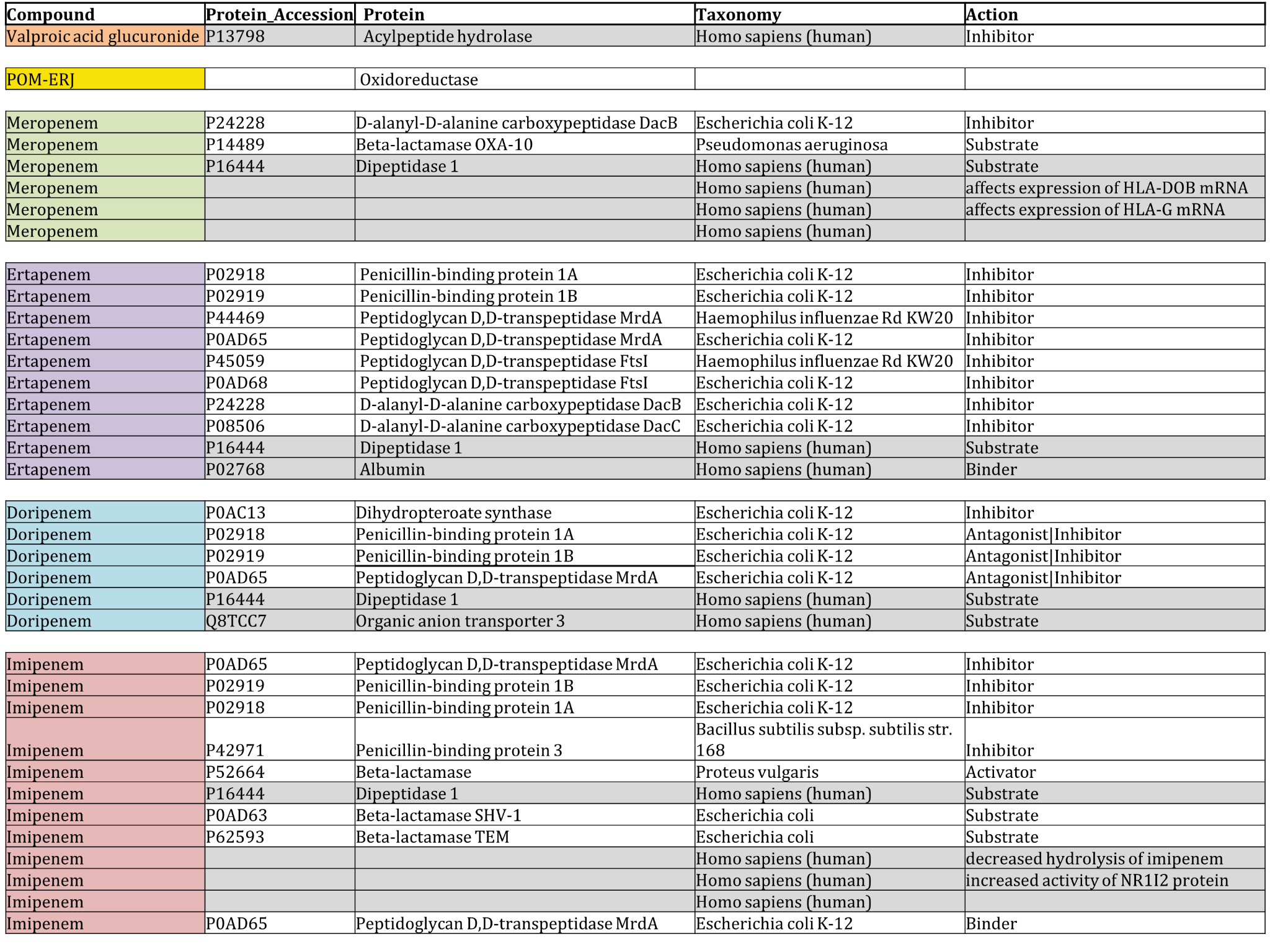


We also carried out predictions of possible such interactions, focusing on VPA-G, POM-ERJ and meropenem. SwissTargetPrediction^2^ proposed 41 possible targets for VPA-G, 14 for POM-ERJ and 82 targets for meropenem, but all with rather low probability. Here we list the proteases from among these hits (highlighting Ser-proteases in darker, Cys-proteases in lighter grey):


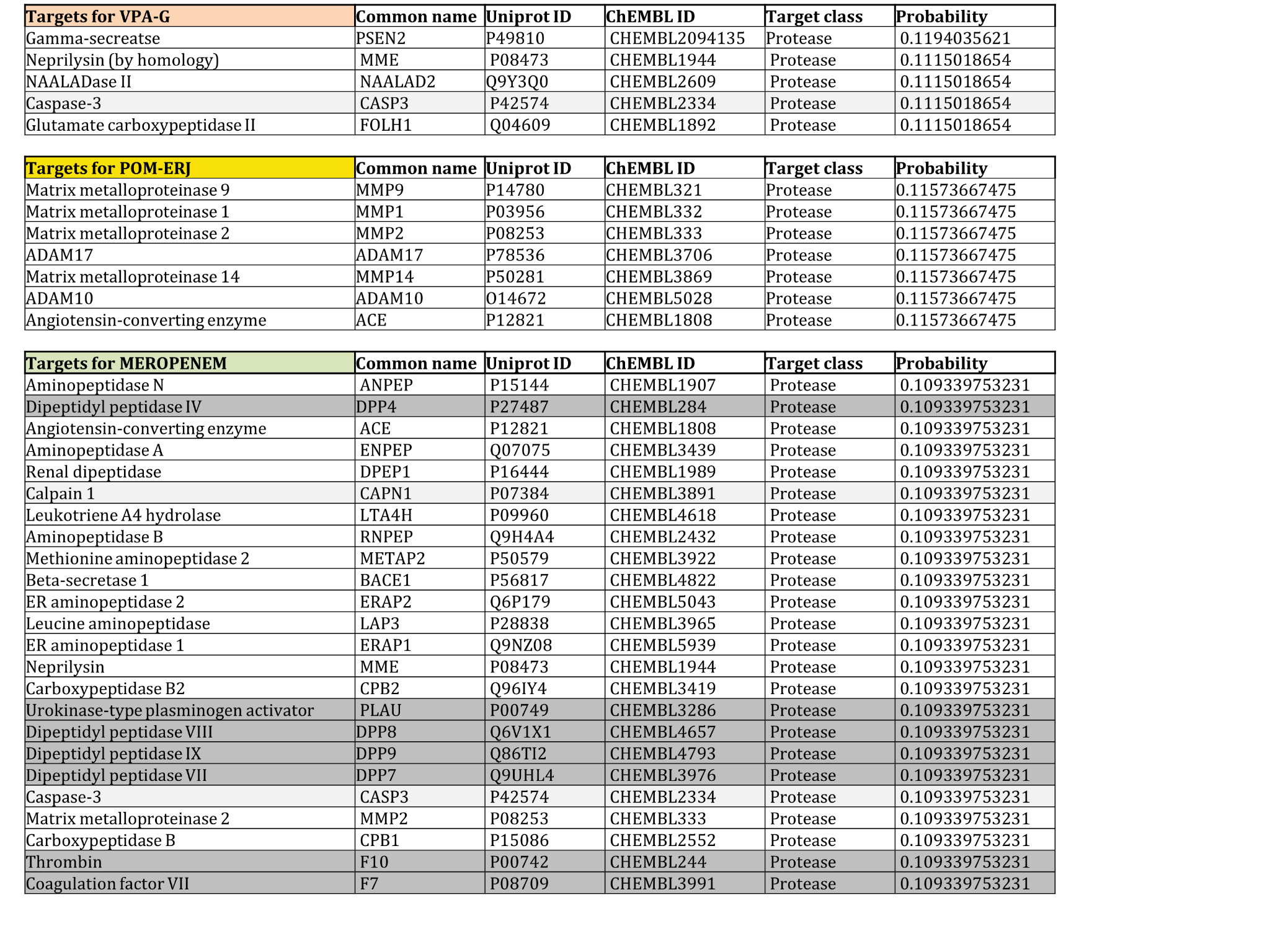


We also applied the Similarity Ensemble Approach (SEA) protocol which also relates proteins based on the similarity among their ligands.^3^ Human enzymes are highlighted in gray in the Table below, but none of the proposed partners are proteases.


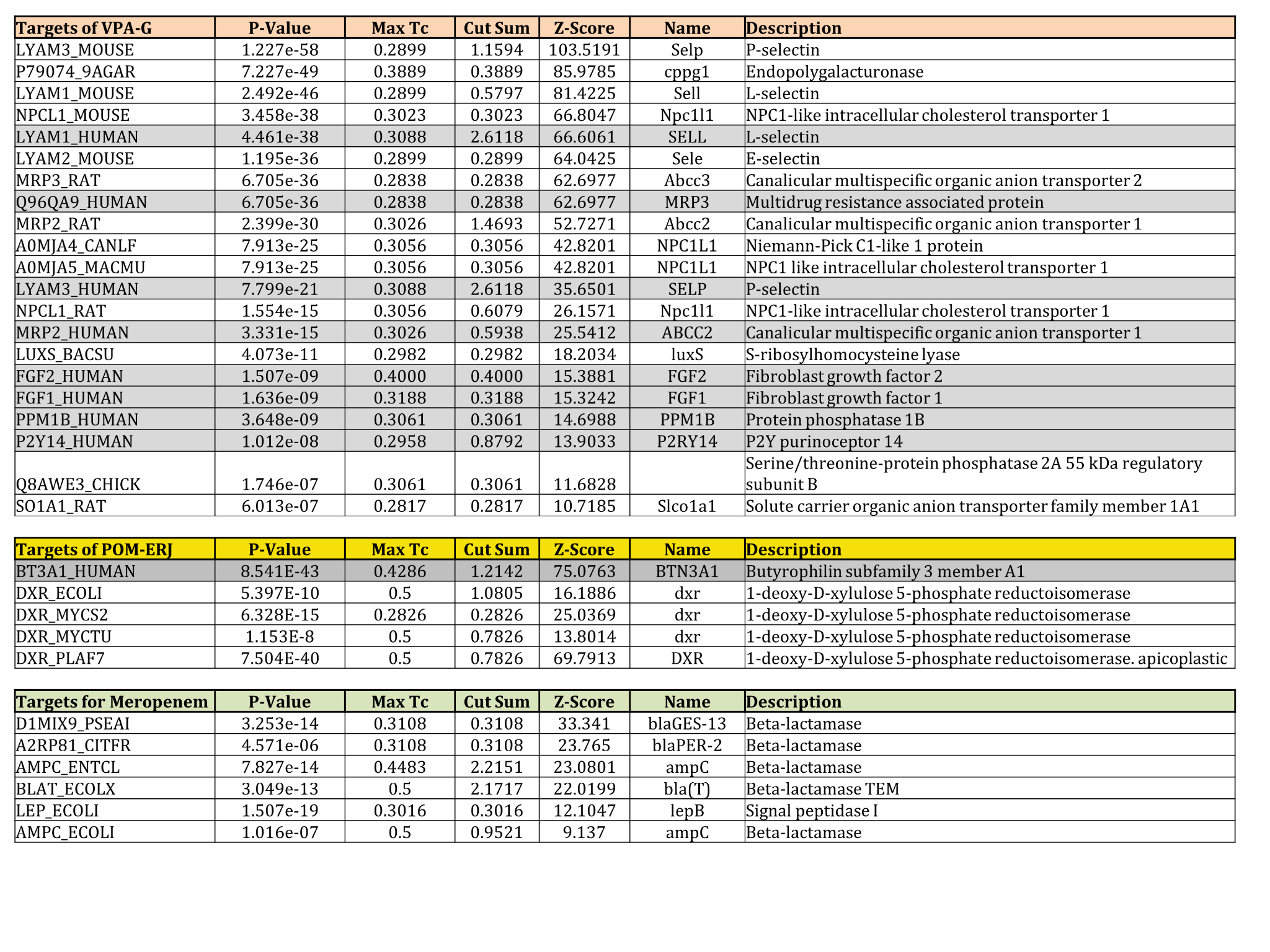


PharmMapper^4^ - an integrated pharmacophore matching platform for potential target identification - was also used, a protocol that provides not only plausible targets, but also estimates the most probable binding mode of the queried substrate. Hits with z-score > 1 were reviewed.

In case of VPA-G 134 targets were proposed, of which two are serine proteases: dipeptidyl peptidase IV (DPP-IV) and thrombin. Since these were also identified by the Swiss TargetPrediction protocol (as possible targets of meropenem), we checked the predicted binding mode of VPA-G within these proteins. In case of DPP-IV, PharmMapper positioned VPA-G over the glycan chain of a surface Asn sidechain (at the solvent exposed top of the propeller domain: Asn85) over 50 Å from the active site. In case of thrombin, VPA-G was fit into the active site in a conformation that places its hydrolysable carbonyl 5.8 Å from the OG atom of the active Ser.


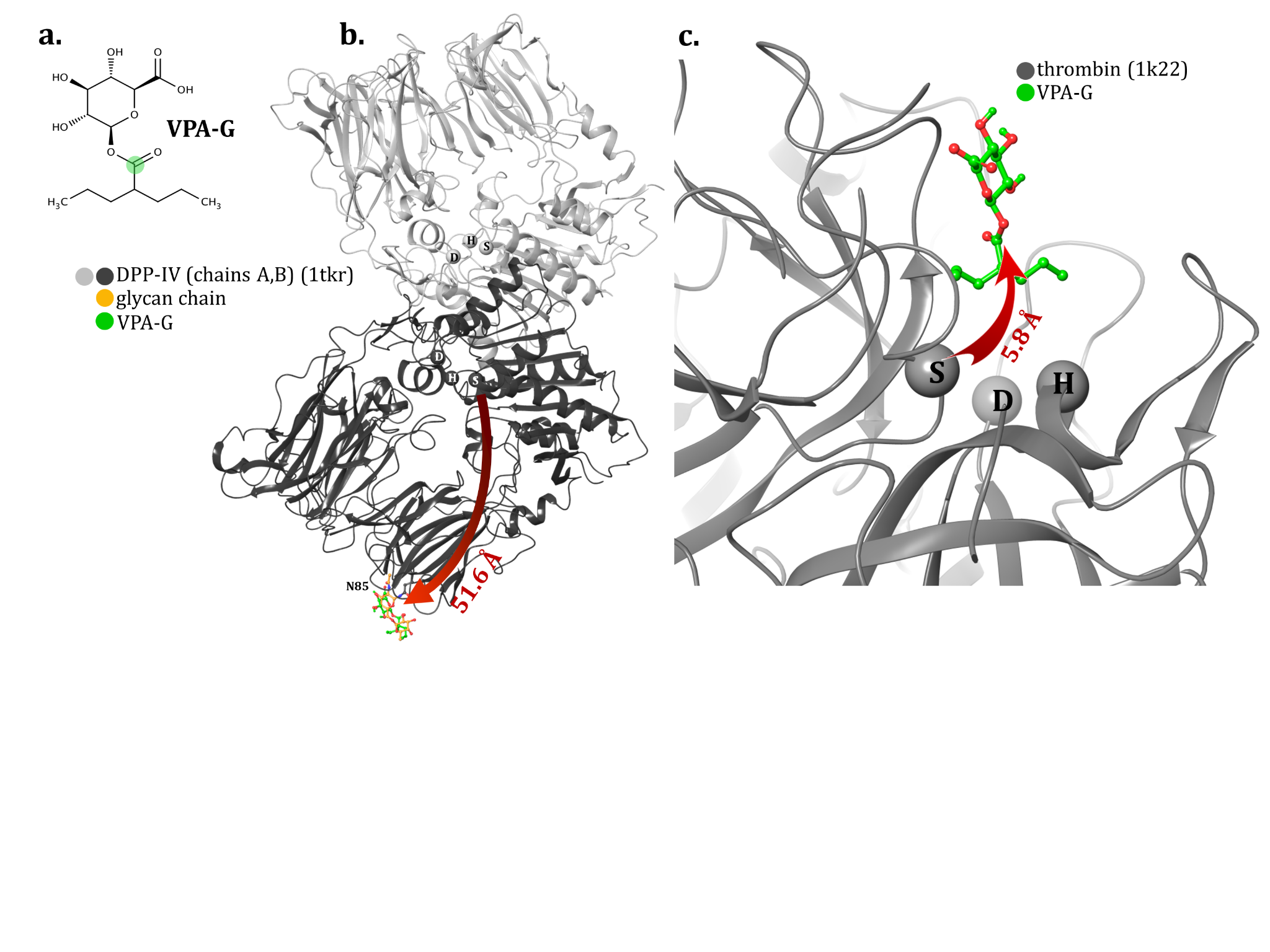
 **Results of PharmMapper search of possible targets of VPA-G. a.)** The structure of VPA-G, with the hydrolysable carbonyl highlighted in green. **b.)** Predicted binding site in case of DPP-IV overlapping the glycan chain of Asn85. **c.)** Binding site near the active Ser of thrombin. The Cα atoms of the catalytic triad (Ser-His-Asp) are shown as spheres. Red arrows indicate the path connecting the active Ser and the hydrolysable carbonyl.

This distance is sufficiently close to view this arrangement as a pre-reaction complex, however since the thrombin catalytic site is in a fully solvent exposed pocket, and because VPA-G forms only a single H-bond with the protein matrix, its expected residence time is short and the probability of a successful attack by the active Ser of thrombin is low.

In light of these findings, it can be concluded that beside APEH, no other serine hydrolase targets of VPA-G are known or could easily be proposed. This is supported by the fact that inhibiting APEH causes the immediate clearance of VPA (see Refs. 20-25 of Main Text), which also makes it unlikely that there is another hydrolase that might similarly be capable of cleaving and recirculating its VPA-G metabolite.

Similarly, DPP-IV and thrombin were found to be possible serine protease targets of POM-ERJ. The attack distance is near optimal in case of DPP-IV, however the in the docked pose a direct clash is formed with Tyr547 of the enzyme.


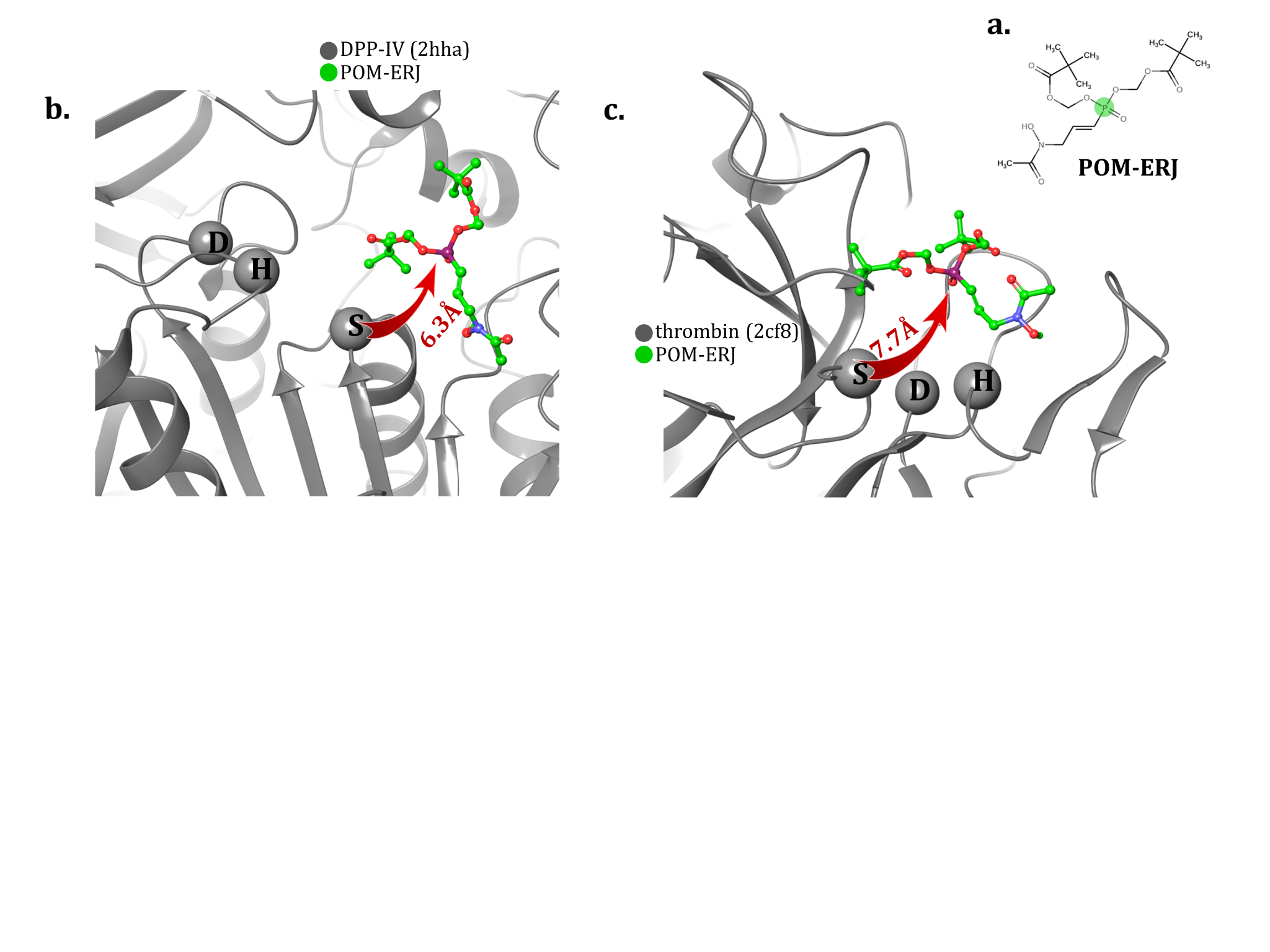
 **Results of PharmMapper search of possible targets of POM-ERJ. a.)** The structure of VPA-G, with the hydrolysable carbonyl highlighted in green. **b.-c.)** Predicted binding sites in case of DPP-IV and thrombin. The Cα atoms of the catalytic triad (Ser-His-Asp) are shown as spheres. Red arrows indicate the path connecting the active Ser and the hydrolysable carbonyl.

In case of meropenem, PharmMapper identified 3 hydrolase targets, among them DPP-IV and thrombin, similarly to the results of the Swiss TargetPrediction search. In the DPP-IV-meropenem complex, the hydrolysable carbonyl of the β-lactam ring is 9.5 Å from the OG atom of the active Ser, while in case of butyrylcholinesterase this same attack-distance is 11.3 Å. These are both too distant to consider as reaction-prone arrangements, however the algorithm found a reasonably compact complex with thrombin as a target for meropenem, just as in case of VPA-G and POM-ERJ. The same concerns can be raised here as in case of VPA-G - the exposed active pocket of thrombin and the low number of specific contacts - but in case of meropenem, experimental evidence also indicates that such an interaction is not formed. In a study investigating the anti-inflammatory and anticoagulant effects of aminoglycoside antibiotics, meropenem was used as a control and it was shown that it does not inhibit either thrombin- or PAF-acetylhydrolase - induced aggregation.^5^ It is also notable that APEH was shown to be more potently inhibited by most organophosphates than their disease-associated targets, acetylcholinesterase and butyrylcholinesterase.^MT26-31^

**
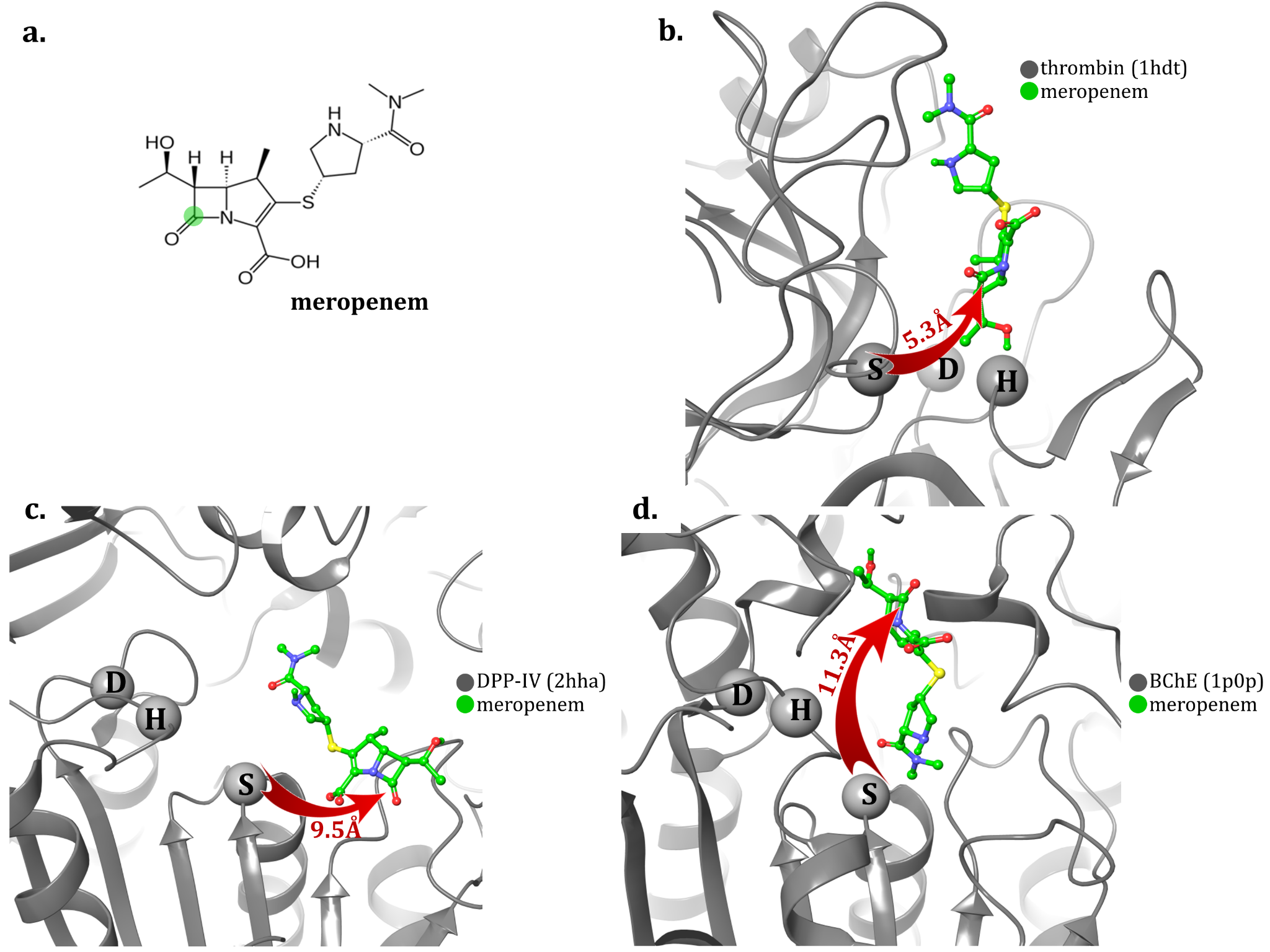
**

**Results of PharmMapper search of possible targets of meropenem. a.)** The structure of meropenem with the hydrolysable carbonyl highlighted in green. **b.)** Predicted binding site in case of thrombin, **c.)** DPP-IV and **d.)** butyrylcholinesterase. The Cα atoms of the catalytic triad (Ser-His-Asp) are shown as spheres. Red arrows indicate the path connecting the active Ser and the hydrolysable carbonyl.

Taken together, these considerations indicate that APEH, carrying Pro506 within its core β-sheet, might indeed have a wider substrate profile than its structural and functional homologues that do not.

**SI Chapter II: Substrate/inhibitor preference of APEH from *Aeropyrum pernix* and *Pyrococcus horikoshii***

**SI Chapter II/a: Reactivity toward chloromethyl ketone decorated peptide inhibitors and meropenem**

Archaeal APEHs show very similar topological buildup to pAPEH and their primary targets are also *N*‑acetylated *N*-terminal residues of oligopeptides but carry a Val in the position corresponding to Pro506 of pAPEH.

To study the catalytic significance of the presence/absence of Pro and the coupled shift of the oxyanion loop we produced APEHs from *Aeropyrum pernix* and *Pyrococcus horikoshii* via bacterial expression.^6^ Although the homology between pAPEH and its archaeal variants is low, the shape and constitution of the active site is similar except for the relaxed conformation of the oxyanion loop in unligated pAPEH, thus the archaeal systems can be used as functional models of pAPEH with a more restricted and confined catalytic site.


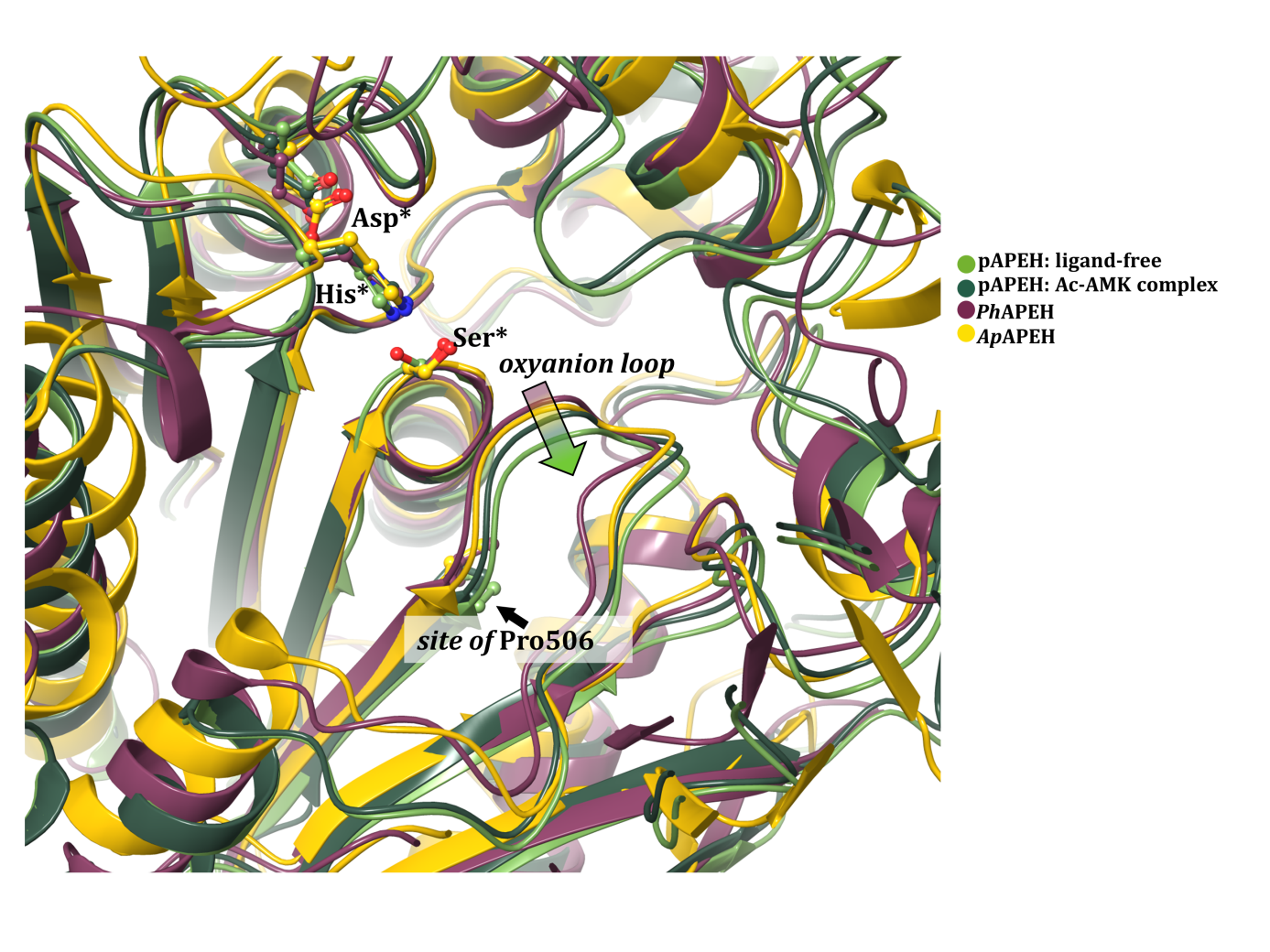


**Comparing the structure of archaeal APEHs to those of the ligand-free and complexed state of pAPEH.** The shift of the oxyanion loop is indicated by the colored arrow. (PDB structures used for creating the image: pAPEH (ligand-fee): 7px8, pAPEH (Ac-AMK complex): this study, *Ph*APEH: 4hxe, *Ap*APEH: 3o4g.)

Their activity was tested at different temperatures (25°C room temperature, RT, 37, 50 and 70°C) using *N*-acetyl-leucine-*p*-nitroanilide (Ac-Leu-pNA) (4mg/ml in 5%DMF-H_2_O) and enzyme activity was monitored using absorbance at 405 nm. The hydrolytic reaction was successful at 90°C within an hour (solution turned yellowish, visible by eye), but within 24h also at room temperature. Although both *Ap*APEH and *Ph*APEH are thermophilic archaeal proteins it was crucial that their activity could be tested at 25°C also, because the antibiotic molecule (Meropenem) is sensitive to temperature, especially in aqueous solutions. Thus, 4 samples were prepared: 1) Ac-Leu-pNA solution in Buffer1 (50mM phosphate, pH=8, 0.3M NaCl, 1mM EDTA, 5mM mercaptoethanol) 2) *Ap*APEH/*Ph*APEH (0.5 mg/ml) with Ac-Leu-pNA in Buffer1, 3) *Ap*APEH/*Ph*APEH with Meropenem (10-fold molar excess) in Buffer1 4) Meropenem solution in Buffer1 (using the same concentration as in 3.). Temperatures were kept constant for 72h. Absorbance was tested after 24 and 72h on all samples. After 72h the samples treated with Meropenem were washed with 20-fold excess of Buffer1 to wash away non-covalently bound compounds and a fresh solution of Ac-Leu-pNA was added. The absorbance of the solution was measured after 24h incubation at 25°C. Also a control sample set was prepared with serine-protease inhibitor cocktail (cOmplete tablets, Roche) to check if *Ap*APEH and *Ph*APEH can be inhibited the regular way. The protein samples (0.5 mg/ml in Buffer1) were mixed with inhibitor cocktail (according to manufacturers’ instructions) and after 1 and 24h Ac-Leu-pNA substrate was added to the mixture and absorbance (405nm) was measured. It should be noted that while pAPEH was able to hydrolase both Ac-Leu-pNA and Ac-Ala-pNA within 1 h, *Ap* and *Ph*APEHs produced no detectable hydrolysis with substrate Ac-Ala-pNA (that is specific for pAPEH). Thus, we found that the archaeal variants are quite selective toward their specific substrate Ac-Leu-pNA, and neither *Ap*APEH nor *Ph*APEH is inhibited by meropenem, the bulky carbapenem antibiotic that is a potent inhibitor of pAPEH.


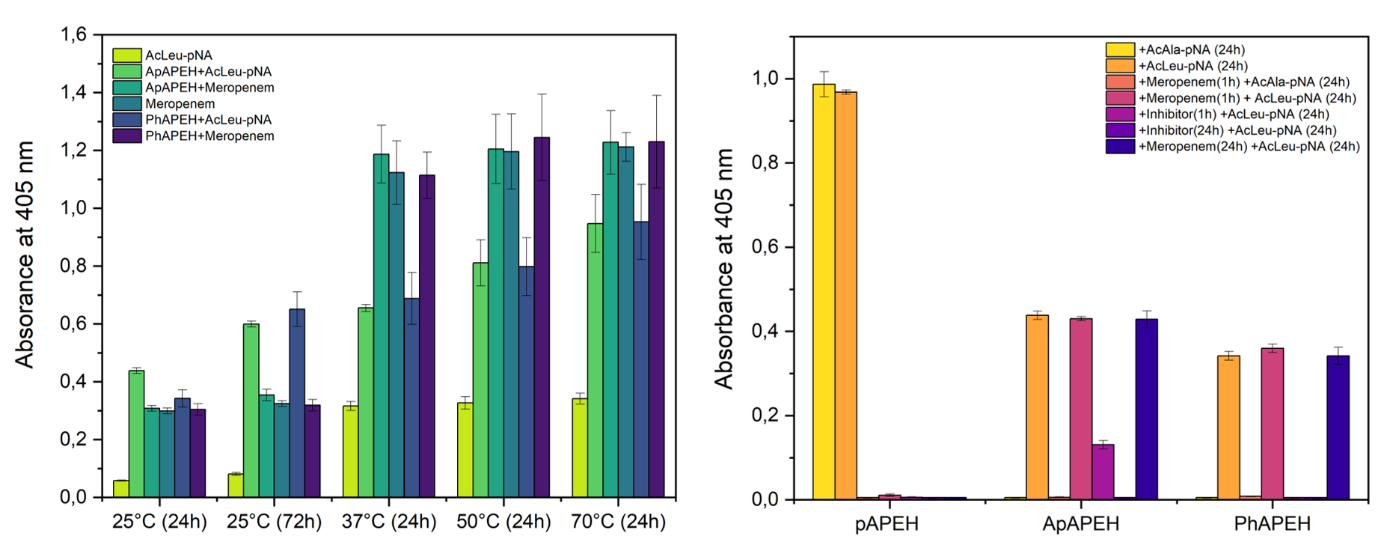
**Reactivity of APEHs against substrates acetyl-leucyl-p-nitroanilide (AcLeu-pNA), acetyl-alanyl-p-nitroanilide (AcAla-pNA) and meropenem.** Although both archaeal enzymes showed higher activity at elevated temperatures, decomposition of Ac-Leu-pNA and Meropenem at these temperatures is also evident. Archaeal APEHs show reduced but detectable activity even at 25°C (left panel). Activity tests conducted at 25°C are shown on the right panel. All values are the average of 3 parallel sample measurements showing the error.


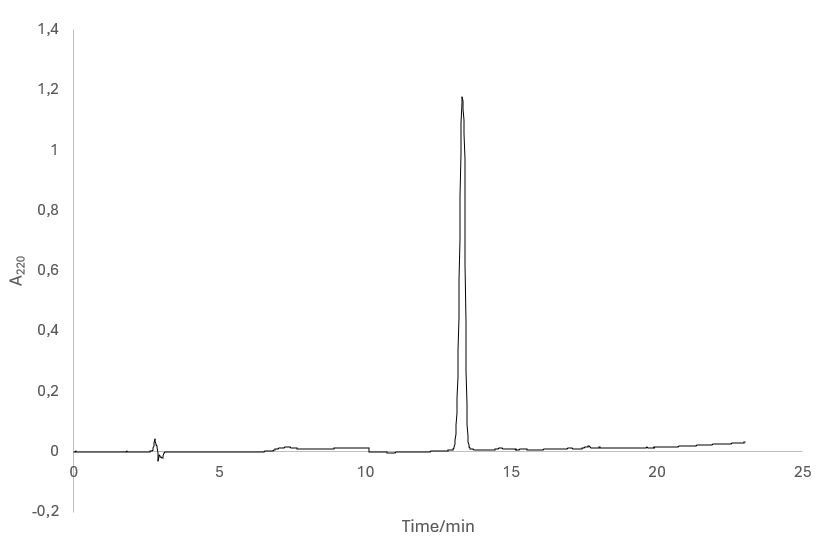

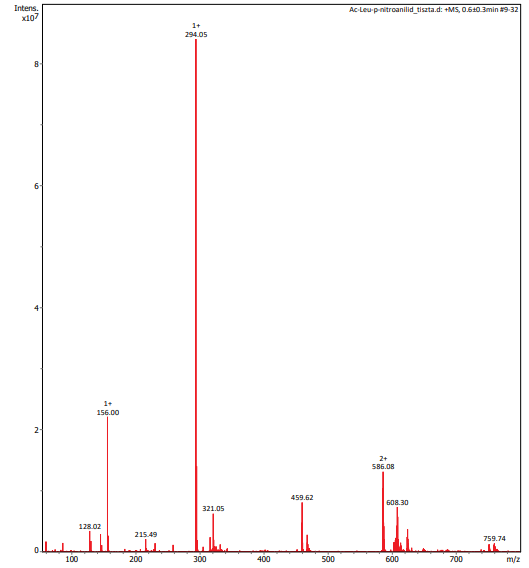


**Characterization of acetyl-alanyl-p-nitroanilide.** *Analytical RP-HPLC*: Column:Thermo BDS Hypersil C18 4.6 mm x 10 cm, 3 µm; Gradient: 0 min 0% B, 1 min 0% B, 16 min 90% B, Flow rate: 2 ml/min, A eluent: 0.1% TFA/water, B eluent: 0.1% TFA in acetonitrile-water (80:20, v/v %), R_t_: 13.2 min. *ESI-MS:* Instrument: Bruker Esquire 3000 plus (Germany), Method: Positive ion electrospray ionization, Solvent: 0.1% acetic acid in acetonitrile-water (50:50, V/V), Soluble in water-acetonitril (9:1 V/V). M_calc._: 293.13 g/mol, M_meas._: 293.05 g/mol

**SI Chapter II/b: Crystallographic study on meropenem binding of *Aeropyrum pernix* APEH**

Attempts were made to produce co-crystals of archaeal *Ap*APEH and meropenem. Previously *Ap*APEH co-crystals with a covalent chloromethyl ketone inhibitor (containing Ac-Phe-CMK meeting the primary substrate binding site-specificity of *Ap*APEH) were obtained using both a co-crystallization protocol and by soaking the crystals into the inhibitor solution^7^. We hypothesized that the D524A mutation could loosen up the active site providing more flexibility for accommodation of the carbapenem ring that is bulkier than the peptide moieties of protease substrates. Though the catalytic activity of D524A *Ap*APEH is significantly lower than that of the wild type enzyme, substrate binding and initial complex formation is only moderately affected by the mutation (measured in presence of peptide substrates and inhibitors)^8^, so we set up the experiment expecting a non-covalent binding.

Co-crystallization was carried out following previously determined protocol (PDB: 3o4h). The crystals were then soaked for a lengthy period in a cryo-solution saturated by meropenem, shifting the pH to neutral. To prevent the competition of a non-polar small molecule for the substrate binding pocket, hyperbranched polyglycerol (HbPG) was used as cryo-protectant. Data collection was carried out at EMBL-Hamburg beamline P13, at DESY^9^ at 100K. Data collection and refinement statistics are compiled in the table below.

**Crystallographic data for co-crystallization result of *Ap*APEH and meropenem:**

| **Crystallization** | |
| --- | --- |
| Setup | hanging drop method, at 20°C, 1.0 / 1.0 / 0.5 volume ratio of  crystallization sol. / protein sol. / meropenem sol. |
| Crystallization solution | 0.45 mM EDTA, 33 mM sodium acetate buffer pH=5.0, 1.2% PEG Mw 4000 |
| Protein solution | 0.24 mM *ApAPEH* D524A in 20 mM Tris pH=8.0 |
| Meropenem solution | 62.5 mM meropenem in methanol |
| Cryo solution | 30% w/w HbPG solurion , pH 7.0, saturated with meropenem (containing undissolved meropenem). Soaking period: 2 weeks. |
| **Data collection and data reduction*** | |
| Synchrotron source | PETRA III, EMBL-Hamburg, DESY, beamline: P13 |
| Wavelength (Å) | 0.9763 |
| Temperature (K) | 100 |
| Space group | P1 |
| Cell dimensions  *a*, *b*, *c* (Å)  *α*, *β*, *γ* (°) | 71.042 98.283 98.902 105.51 103.19 100.39 |
| Unique reflections (last shell) | 105834 (7565) |
| Resolution (Å) (last shell) | 40.0-2.30 (2.36-2.30) |
| *R_meas_* (last shell) | 0.081 (1.022) |
| *I/σ(I)*  (last shell) | 9.36 (1.05) |
| Completeness (%) (last shell) | 98.1 (95.0) |
| Redundancy (last shell) | 3.0 (1.8) |
| *CC_1/2_*  (last shell) | 0.997 (0.502) |
| *Wilson B* (Å^2^) | 62.56 |
| ***Refinement**** | |
| Resolution (Å) | 39.2-2.30 |
| No. reflections (No. reflections in cross validation) | 105790 (2101) |
| *R_work_, R_free_* | 0.1968, 0.2286 |
| Model content | - 2 *ApAPEH* dimers: chains A-B and C-D. Molecules A and C are in closed, B and D in open conformations - 4 fragments of HbPG - 251 water molecules |
| No. non-hydrogen atoms  Protein  Ligands: HbPG fragments  Water | 16716  23  251 |
| B-factors  Protein  Ligands: HbPG fragments  Water | 70.06  81.44  59.45 |
| R.m.s. deviations  Bond lengths (Å)  Bond angles (°) | 0.002  0.562 |
| Ramachandran plot (%)  Outliers / Allowed / Favored | 0 / 0.55 / 97.69 |
| PDB ID | 9S6B |

* The dataset was processed using the XDS^10^ package. The phase problem was solved by molecular replacement with Phaser^11^ using a dimer of ApAPEH from isostructural PDB structure 3O4H (uncomplexed D524A mutant ApAPEH) ^7^  after removal of the segments 444-446, 523-525 and 555-557 from the model. The structure was refined using Phenix^12^. First, rigid body refinement of individual domains was carried out. In later refinement cycles torsion angle non-crystallographic symmetry restraints were applied. Bulk solvent, coordinate, individual B-factor and TLS refinement (initially with one TLS group/chain, at the later stages of refinement with automatically defined TLS groups) were included. B factors were reset before the final refinement cycles. Model building was carried out using Coot^24^. The structure was validated using MolProbity^13^ and Coot^14^.

Despite the high excess of meropenem and combined co-crystallization and soaking method, the drug molecule was not found in the electron density maps of either the open or the closed molecules of *Ap*APEH. As expected, the His-loops of the open monomers were disordered. Surprisingly, an increase in flexibility was detected also in regions further from the mutation site: at some surface loops, one side of molecule C exposed to the solvent region and even within the active site of one of the closed molecules. This could be explained by the combination of the absence of stabilizing effect of nonspecifically bound small molecular cryoprotectant at the enzyme surface and the loss of the side chain interactions of Asp524 caused by the mutation. The branched HbPG polymer did not access the active site region, so it could not hinder meropenem binding.


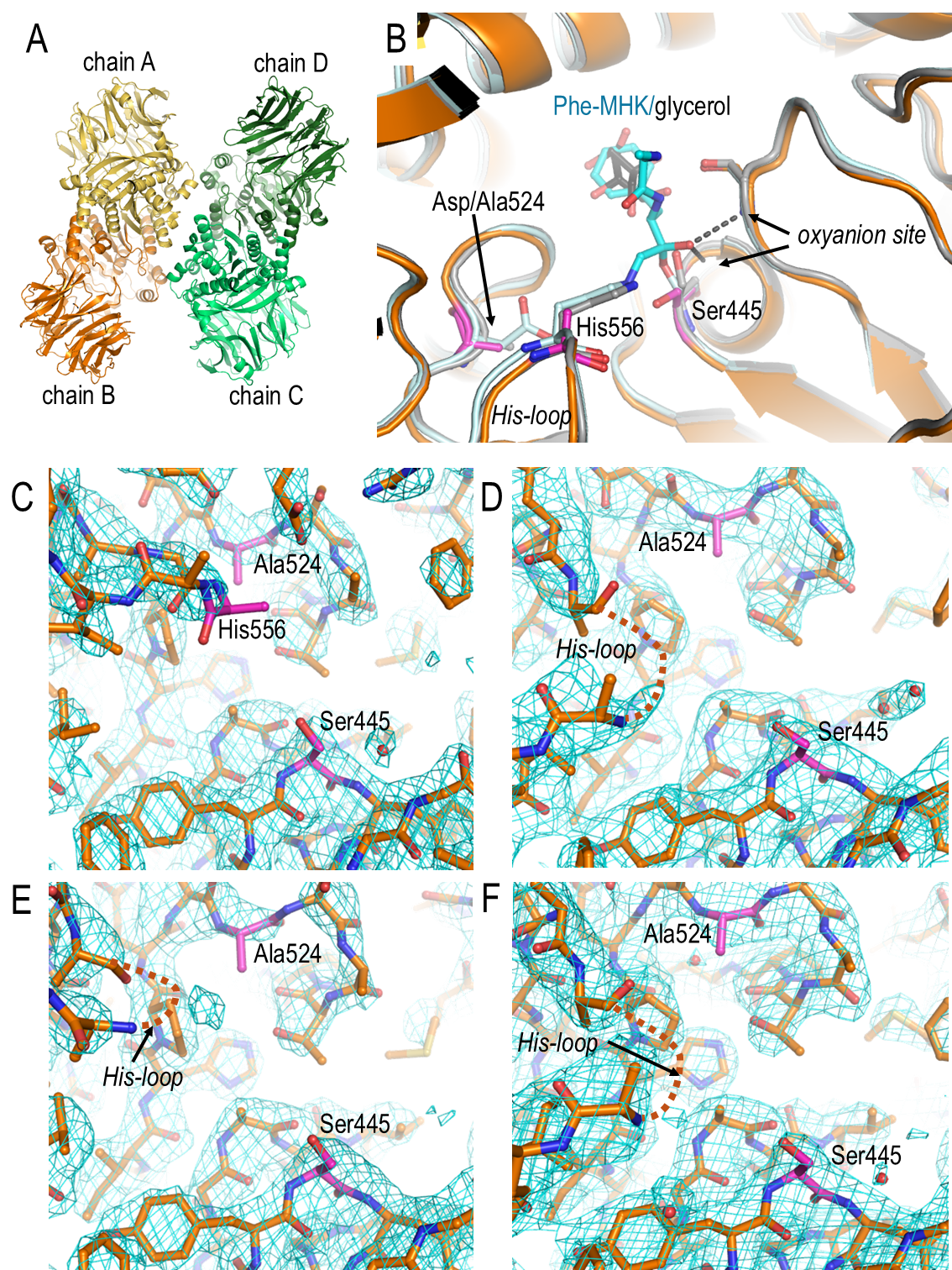


**Crystal structure of *Ap*APEH co-crystallized with meropenem**. A) The unit cell contains two homodimers, A-B and C-D. The molecules in open (catalytically latent) conformations are shown in darker shades. B) Comparison of the closed monomer (chain A, in orange, with the catalytic triad shown with magenta carbon atoms) with that of the uncomplexed *Ap*APEH D524A mutant form (PDB: 3O4H, grey) and wild type *Ap*APEH - chloromethyl ketone covalent complex (PDB: 4RE6, light blue). In the latter two the S1 substrate binding pocket is occupied with glycerol and the phenylalanyl-methylhemiketone moiety (shown in darker shades). C-F) 2*F_o_-F_c_* type electron density map contoured at the 1.0 σ level around the active site for molecules A, B, C and D, respectively. Note, in molecules B,C and D, a 2-3 residue long segment of the His-loop including His556 could not be located in the electron density (dotted line), so it was not built in the structure.

In summary, the fact that similar conditions that allow for the in situ binding of chloromethyl ketone peptide derivatives to *Ap*APEH did not result in the formation of a meropenem complex, supports the results of our in vitro binding study: the archaeal enzymes equipped with shifted and more rigid oxyanion loops are less adaptable for ligand binding.

**Figure S1. Workflow of cryo-EM data processing for APEH-AcAMHK (EMD-51464, 9GNE).**

A) Data processing workflow using cryoSPARC, showing 2D classes (left panel) and a typical micrograph for APEH-AcAMK complex (particles circled in red). B) Maps obtained applying C1 and D2 point group symmetries in 3D refinement and the calculated map to map FSC curves (from cryoSPARC). Initial model of 7px8 was docked in the sharpened maps using Phenix Dock in Map. (Chimera 0.0581 threshold level).


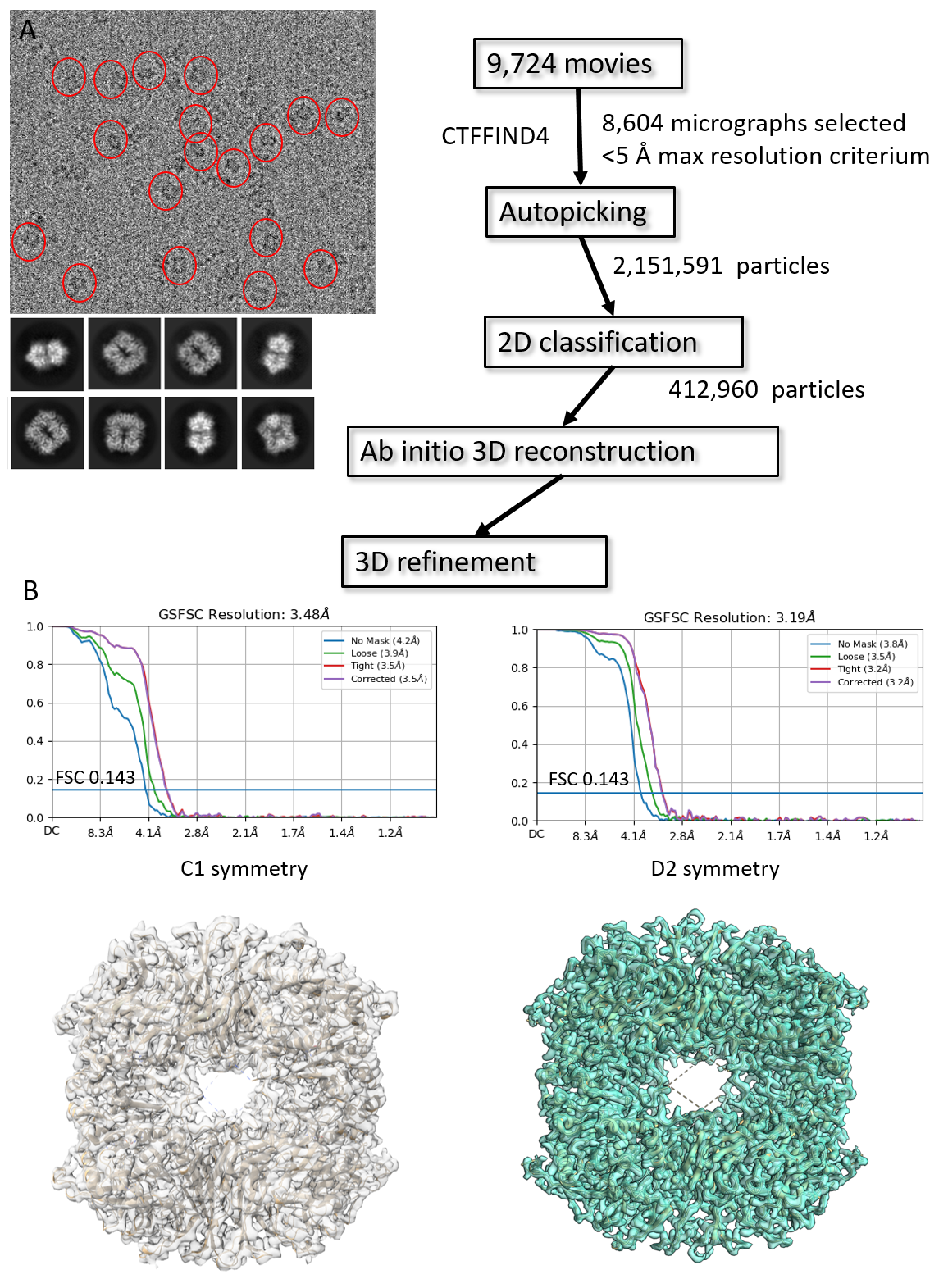


**Figure S2. Validation of the cryo-EM data processing for APEH-AcAMHK (EMD-51464, 9GNE).**

A) The calculated half-maps FSC curves using Phenix and B) the calculated model to map FSC curves using Phenix validation.


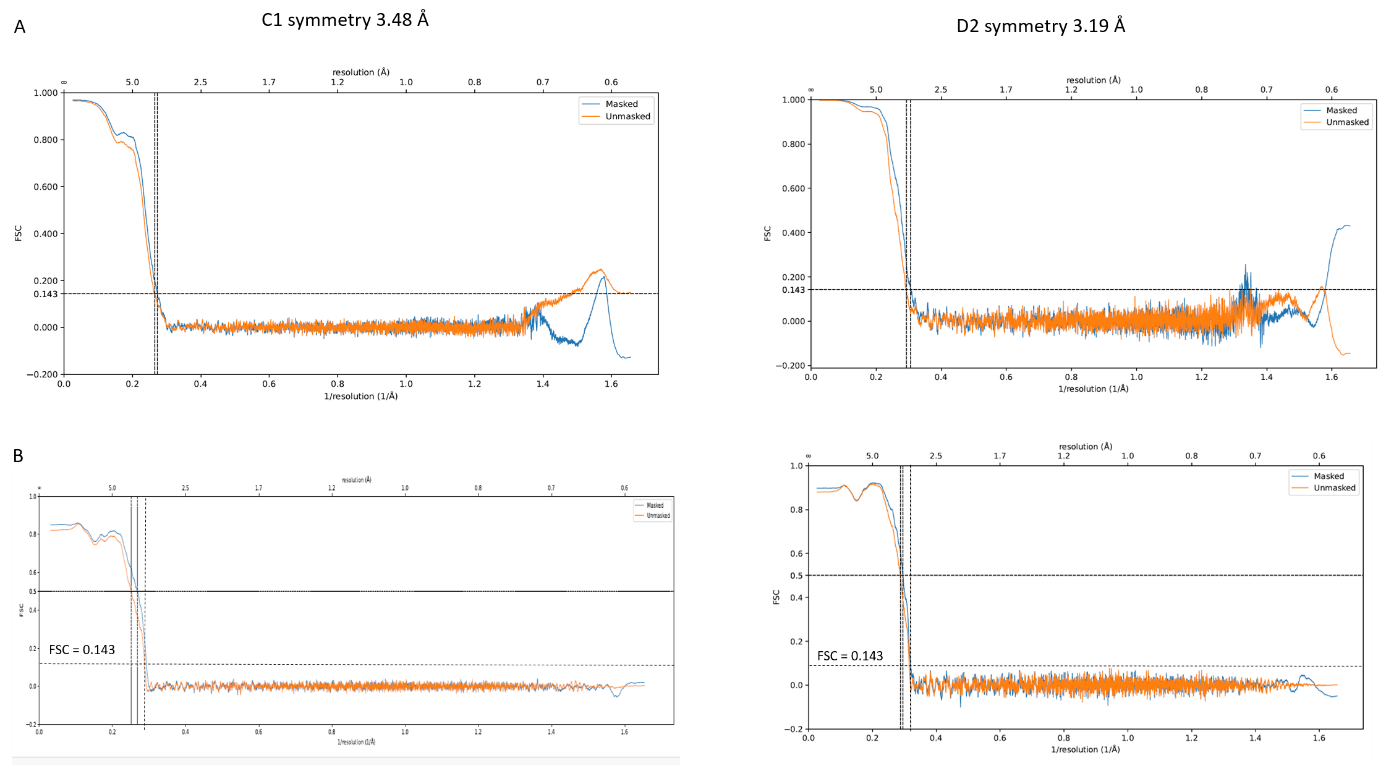


**Figure S3. Workflow of cryo-EM data processing for APEH-DMP (EMD-51501, 9GOU).**

A) Data processing workflow using cryoSPARC, showing 2D classes (left panel) and a typical micrograph for APEH-DMP complex (particles circled in red). **B**) Maps obtained applying C1 and D2 point group symmetries in 3D refinement and the calculated map to map FSC curves (from cryoSPARC). Initial model of 7px8 was docked in the sharpened maps using Phenix Dock in Map. (Chimera 0.0781 threshold level).


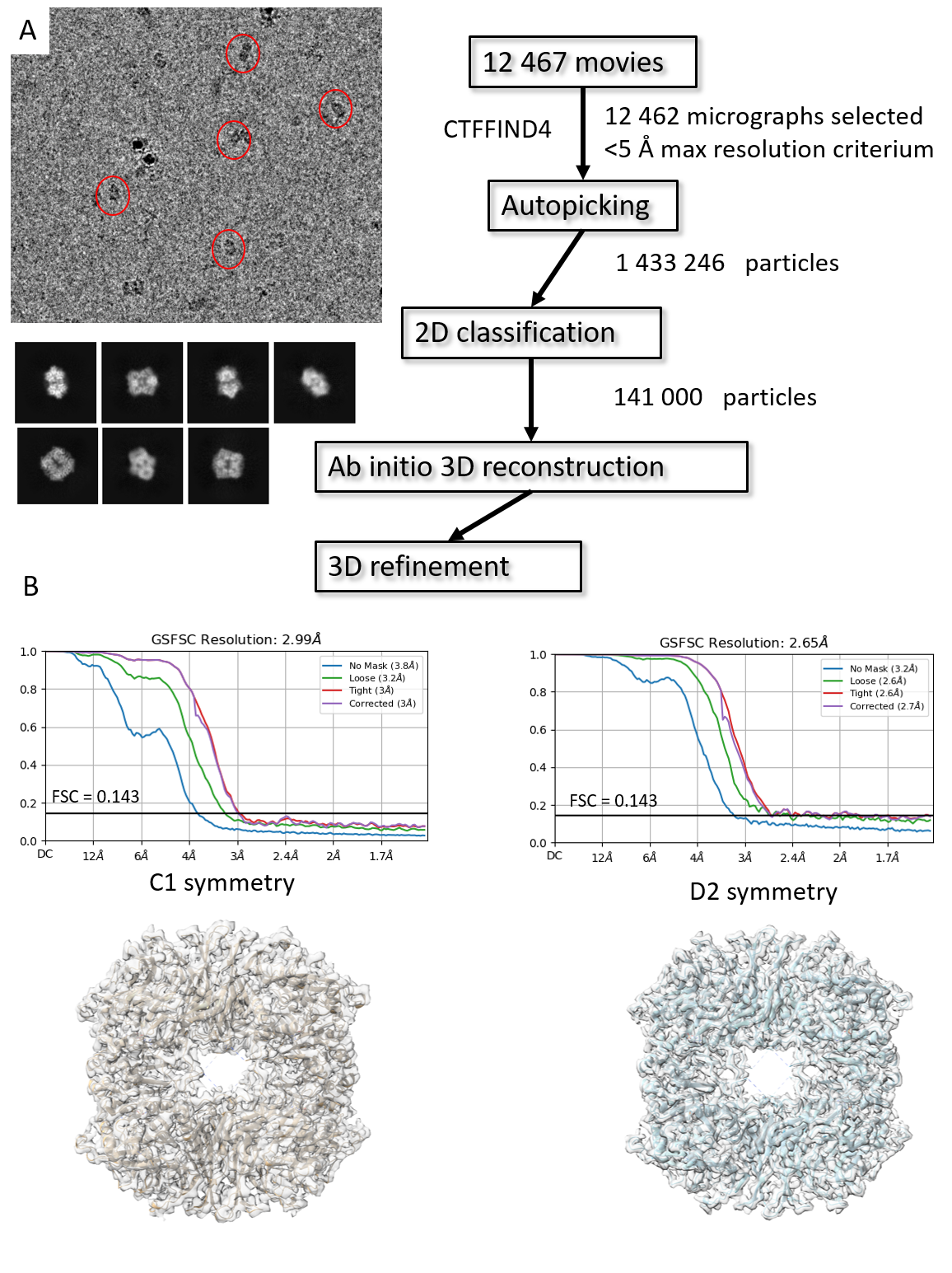


**Figure S4. Validation of the cryo-EM data processing for APEH-DMP (EMD-51501, 9GOU).**

A) The calculated half-maps FSC curves using Phenix and B) the calculated model to map FSC curves using Phenix validation.


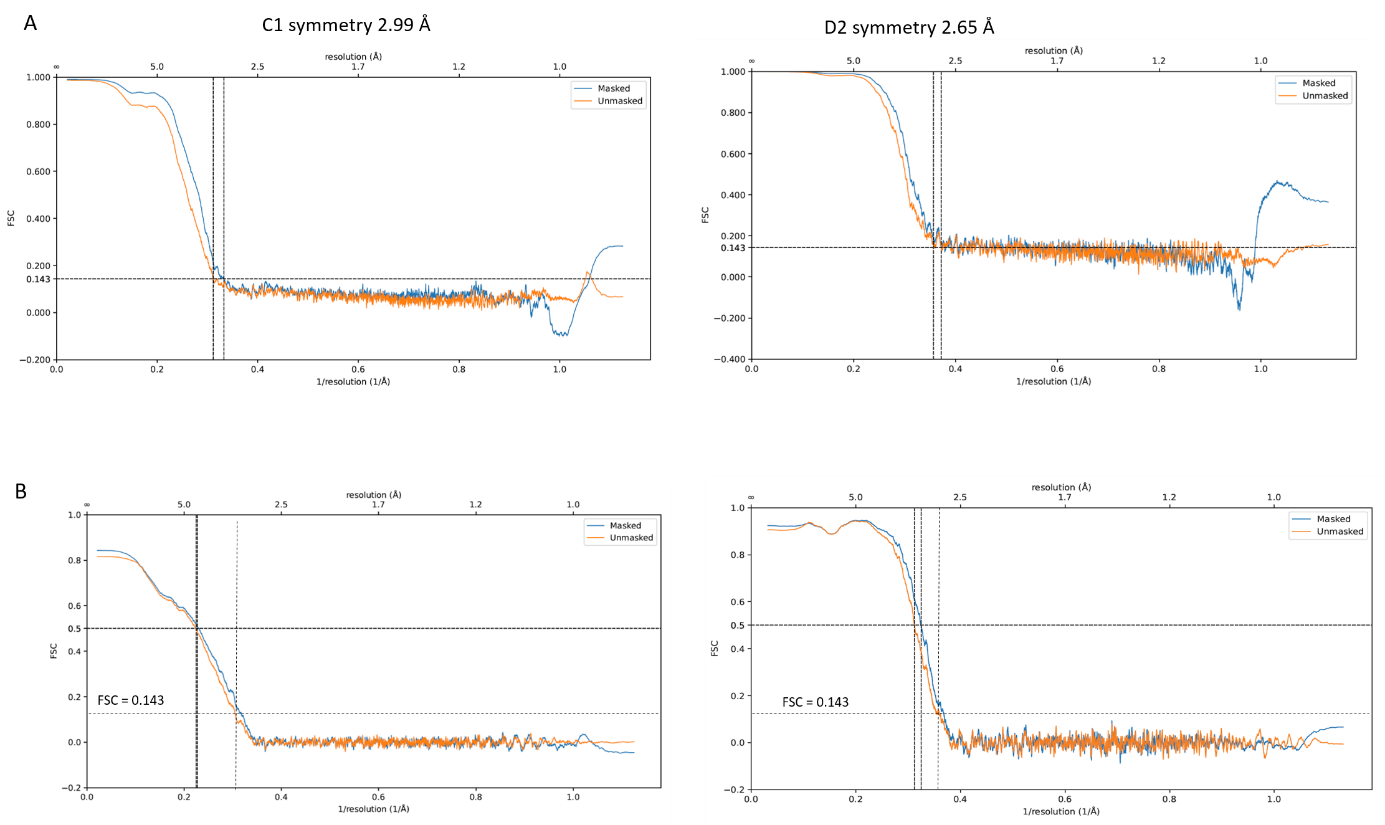


**Figure S5. Workflow of cryo-EM data processing for APEH-AES (EMD-52489, 9HXQ).**

A) Data processing workflow using cryoSPARC, showing 2D classes (left panel) and a typical micrograph for APEH-AES complex (particles circled in red). **B**) Maps obtained applying C1 and D2 point group symmetries in 3D refinement and the calculated map to map FSC curves (from cryoSPARC). Initial model of 7px8 was docked in the sharpened maps using Phenix Dock in Map. (Chimera 0.0781 threshold level).


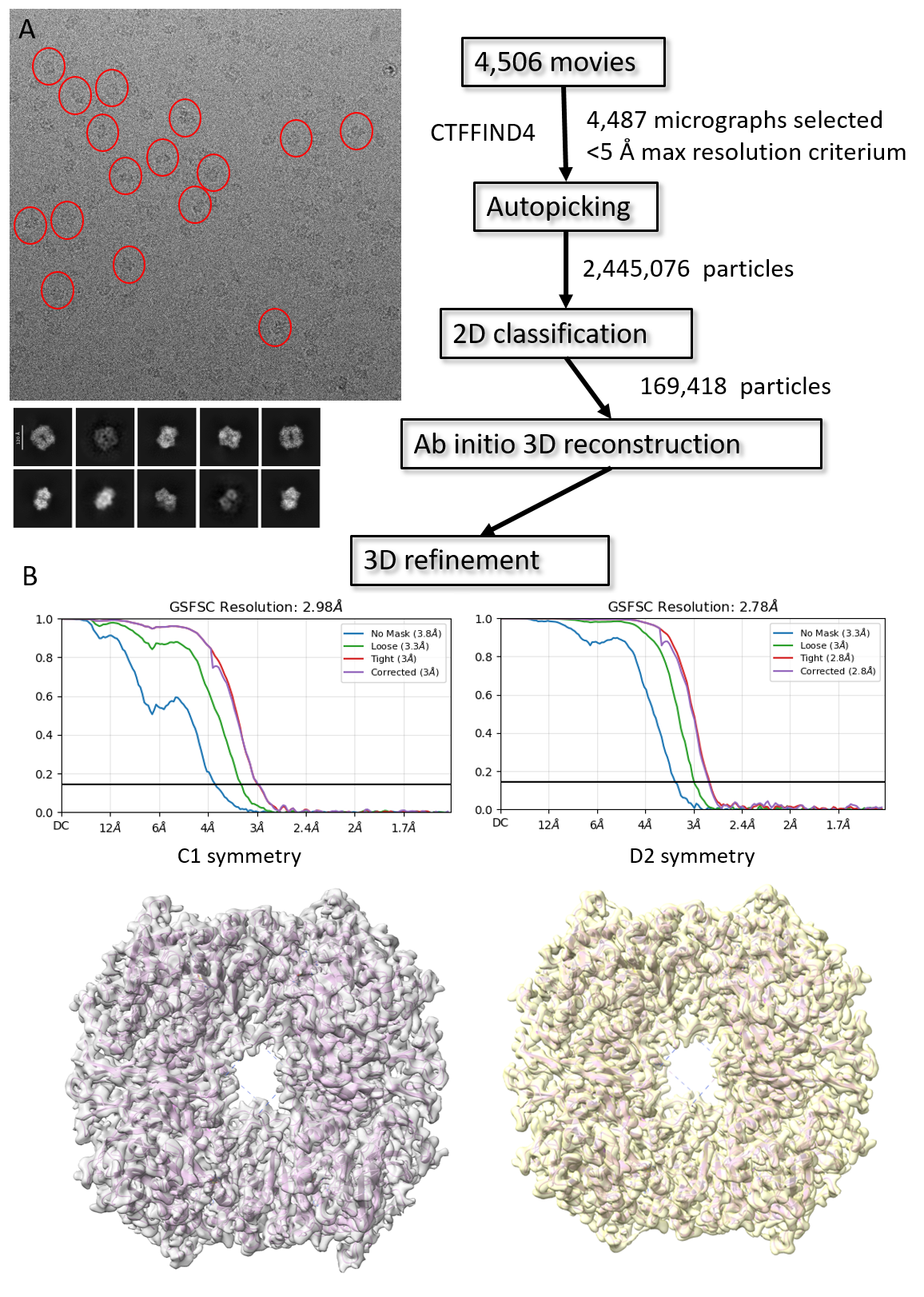


**Figure S6. Validation of the cryo-EM data processing of APEH-AES (EMD-52489, 9HXQ) and structure evaluation.**

A) The calculated half-maps FSC curves using Phenix and B) the calculated model to map FSC curves using Phenix validation. C) The shallow and rather hydrofobic S2 binding pocket accomodates the aminoethylbenzene group of the hydrolysed AEBSF. D) The covalent binding of AES to catalytic Ser587 of APEH, showing the considerable shifts compared to the apoAPEH structure (PDB: 7px8). E,F) Local resolution-colored maps of the pAPEH tetramer (topview) and near the active site calculated by Phenix, contoured at the 0.0516 threshold level. F) The local resolution value for the AES ligand and the oxyanion loop is 2.9 Å (cyan).


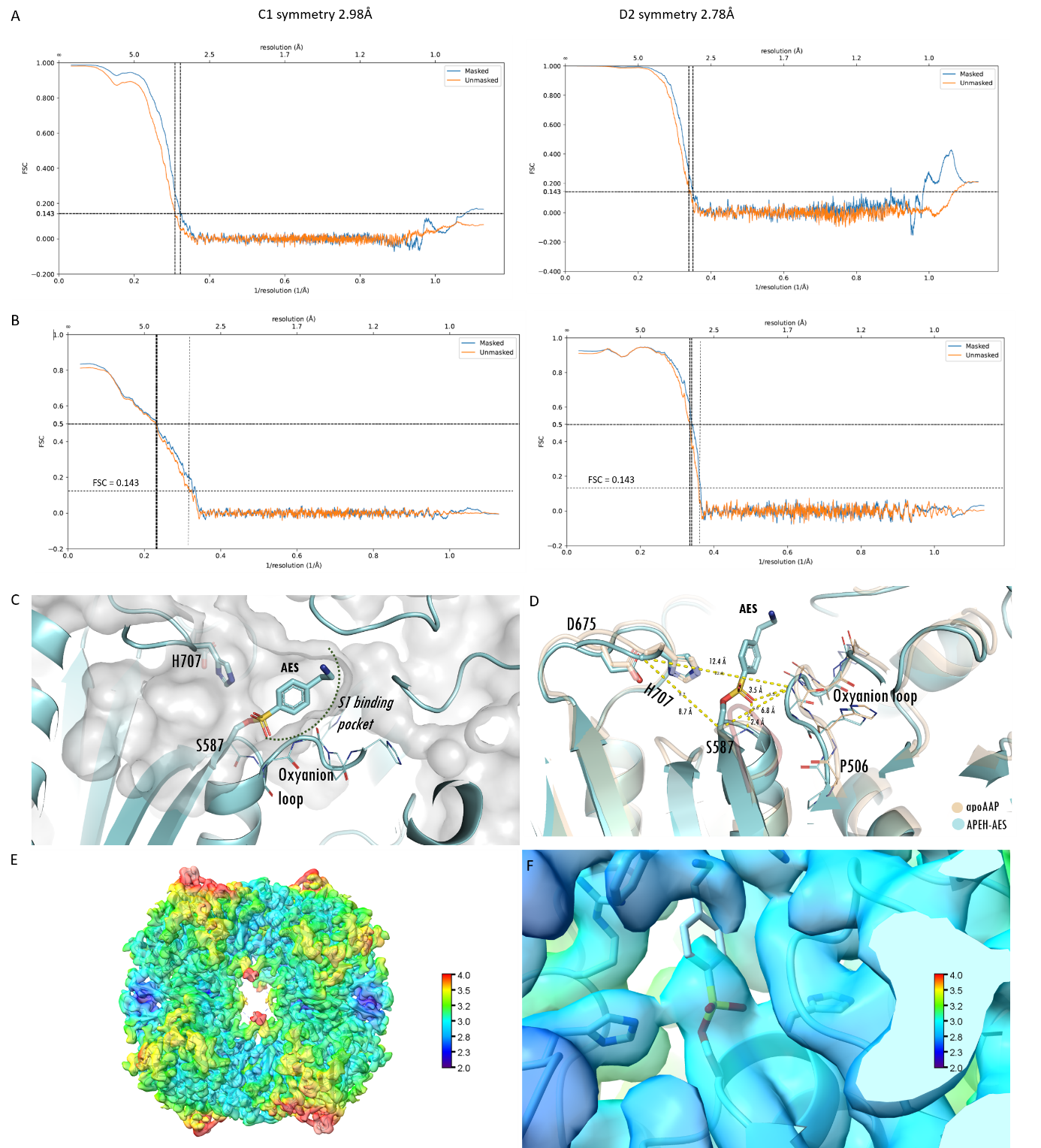


**Table S1**. **Cryo-EM data collection, refinement and validation statistics for APEH-AcAMHK, APEH-DMP and APEH-AES.**

| **Data collection** | **APEH-AcAMHK** | **APEH-DMP** | **APEH-AES** |
| --- | --- | --- | --- |
| Microscope | CRYOARM 300II | KRIOS G4 | KRIOS G4 |
| Detector | K3 | Falcon 4i | Falcon 4i |
| Magnification | ⨯80,000 | ⨯165,000 | ⨯165,000 |
| Voltage (kV) | 300 | 300 | 300 |
| Electron exposure (e/Å^2^) | 40 | 12 | 12 |
| Defocus range (μm) | 0.5 - 2.5 | -1.5 – (-0.5) | -1.5 – (-0.5) |
| Pixel size (Å) | 0.5167 | 0.7519 | 0.7519 |
| **Data processing** | | |  |
| Accession codes | EMD-51464,  PDB ID: 9GNE | EMD-51501,  PDB ID: 9GOU | EMD-52489,  PDB ID: 9HXQ |
| Symmetry imposed | D2* | D2* | D2* |
| Initial particle images (no.) | 2,151,591 | 1,433,246 | 2,445,076 |
| Final particle images (no.) | 412,960 | 141,000 | 169,418 |
| Map resolution (Å) | 3.19 | 2.65 | 2.78 |
| FSC threshold | 0.143 | 0.143 | 0.143 |
| **Refinement** | | |  |
| Model resolution (Å) | 3.2 | 2.6 | 2.8 |
| Model composition | | |  |
| Non-hydrogen atoms | 19244 | 20746 | 20740 |
| Protein residues | 2736 | 2788 | 2788 |
| B-factors (min/max/mean) | | |  |
| Protein | 28.58/117.64/64.54 | 80.01/191.03/118.55 | 59.29/163.90/97.63 |
| ligand | 50.25/71.05/63.26 | 129.64/147.10/138.04 | 115.65/139.28/128.47 |
| r.m.s. deviations | | |  |
| Bond length (Å) | 0.006 | 0.002 | 0.002 |
| Bond angles (°) | 0.624 | 0.478 | 0.511 |
| **Validation** | | |  |
| MolProbility score | 1.22 | 1.65 | 1.05 |
| Clashscore | 2.64 | 7.91 | 2.57 |
|  |  |  |  |
| Rotamer outliers (%) | 0 | 0 | 0 |
| CaBLAM outliers (%) | 1.71 | 2.06 | 1.43 |
| Ramachandran plot (%) | | |  |
| Outliers | 0 | 0 | 0 |
| Allowed | 81 (2.99%) | 95 (3.41%) | 57 (2.07%) |
| Favored | 2655 (97.03%) | 2693 (96.59%) | 2731 (97.97%) |

**models using C1 symmetry not shown*

**Figure S7. The shift resulting in a conformational change that increases the affinity for oxygen (cooperative binding) in hemoglobin.** Upon binding oxygen, a shift in the heme group's structure occurs, resulting in a slight displacement (0.4 Å) of the iron atom and a change in the position of neighboring amino acids. Unbound structure shown in cyan (PDB id: 2hbs) and the oxygen-bound structure in yellow (PDB id: 6bb5). The shift of 0.4 Å is measured at the distance between the iron-ion of the heme and His92 Nε2 atoms.


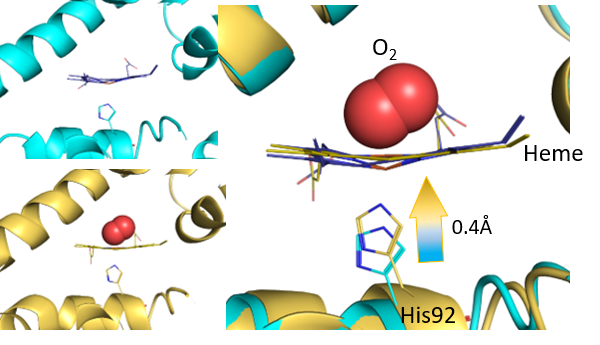


**Figure S8. The binding of even bulky inhibitors to AChE is completed without structural rearrangement of the active site.** The structures of A) apoAPEH (PDBid 7px8) and apoAChE (1j06) showing the catalytic Ser and His residues aligning well (grey circle). The oxyanion loop is longer and in closer proximity to the catalytic Ser in AChE (blue rectangle). The binding pocket forming backbone (also called aging loop in AChE, yellow box) and the mammalian specific flexible insert in APEH (red box) create more space around the active site. B) apoAChE (PDB id: 1j06) and ligand bound structures with VX (PDB id: 2y2u), methamidophos (MeP, PDB id: 2jge) and fenamiphos (FeP, PDB id:2jgf), C) apoAChE (PDB id: 1j06), AChE-DFP (PDB id: 2jgi) and AChE with aged DFP (PDB id: 2jgm): the *aging loop* is moved upon DFP binding and does not change with aging. D) apoAChE (1j06), AChE-VX (2jgh) and AChE with aged VX (2jgl), E) apoAChE (1j06), AChE-MeP (2jge) and AChE with aged MeP (2jgj), F) apoAChE (1j06), AChE-FeP (2jgf) and AChE with aged FeP (2jgk): the *aging loop* is shifted upon aging.

**
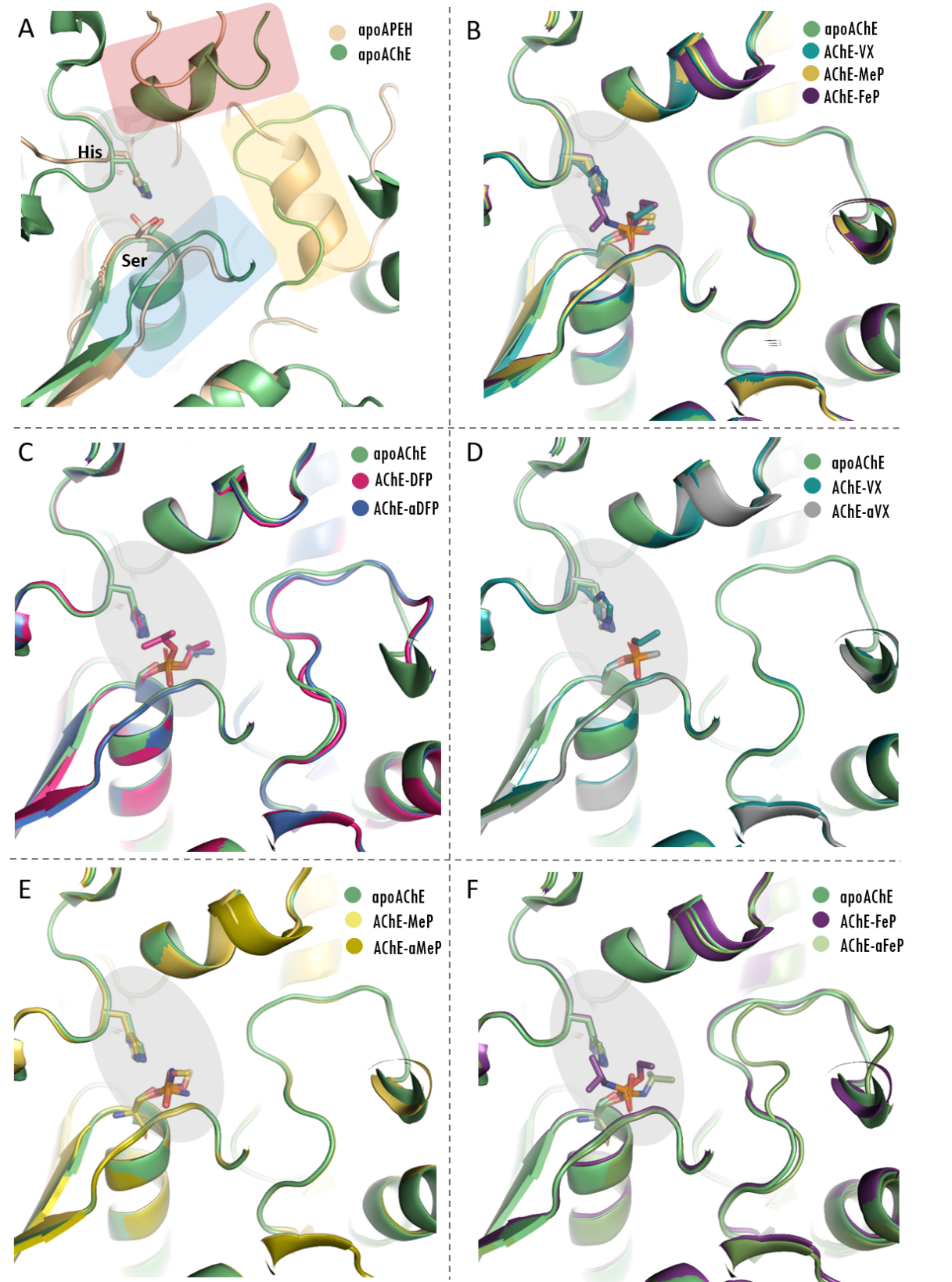
**

**Figure S9.** **Organophosphorous compounds binding to APEH and acetylcholinesterase (AChE).** Leaving groups, indicated by the red circles, are the substituents of the ligand that dissociate in the first reaction step of the serine hydrolase reaction. Substituents that are accommodated by the primary binding pocket (S1 for APEH and acyl-pocket for AChE) are also the groups that take part in the aging reactions (green dotted rectangle). The OPs are arranged by decreasing IC_50_ values measured for APEH. The oxon (P=O bond) forms of OPs – created during natural metabolic processing from the corresponding thion –compounds (P=S bond) – are more susceptible towards the nucleophilic attack of the serine-hydrolase enzymes, thus more reactive and more effective in inhibition of the enzymes.^15,16^ The remaining substituents succumb to the aging process where the negatively charged phosphate is created, leaving the enzyme permanently inhibited, however this process is modulated by the surrounding sidechains.^17^ OPs with more hydrophobic leaving groups bind more effectively to AChE, because of the aromatic sidechains (Phe295, 297, 338, Tyr337, 341) in the proximity of the binding site.


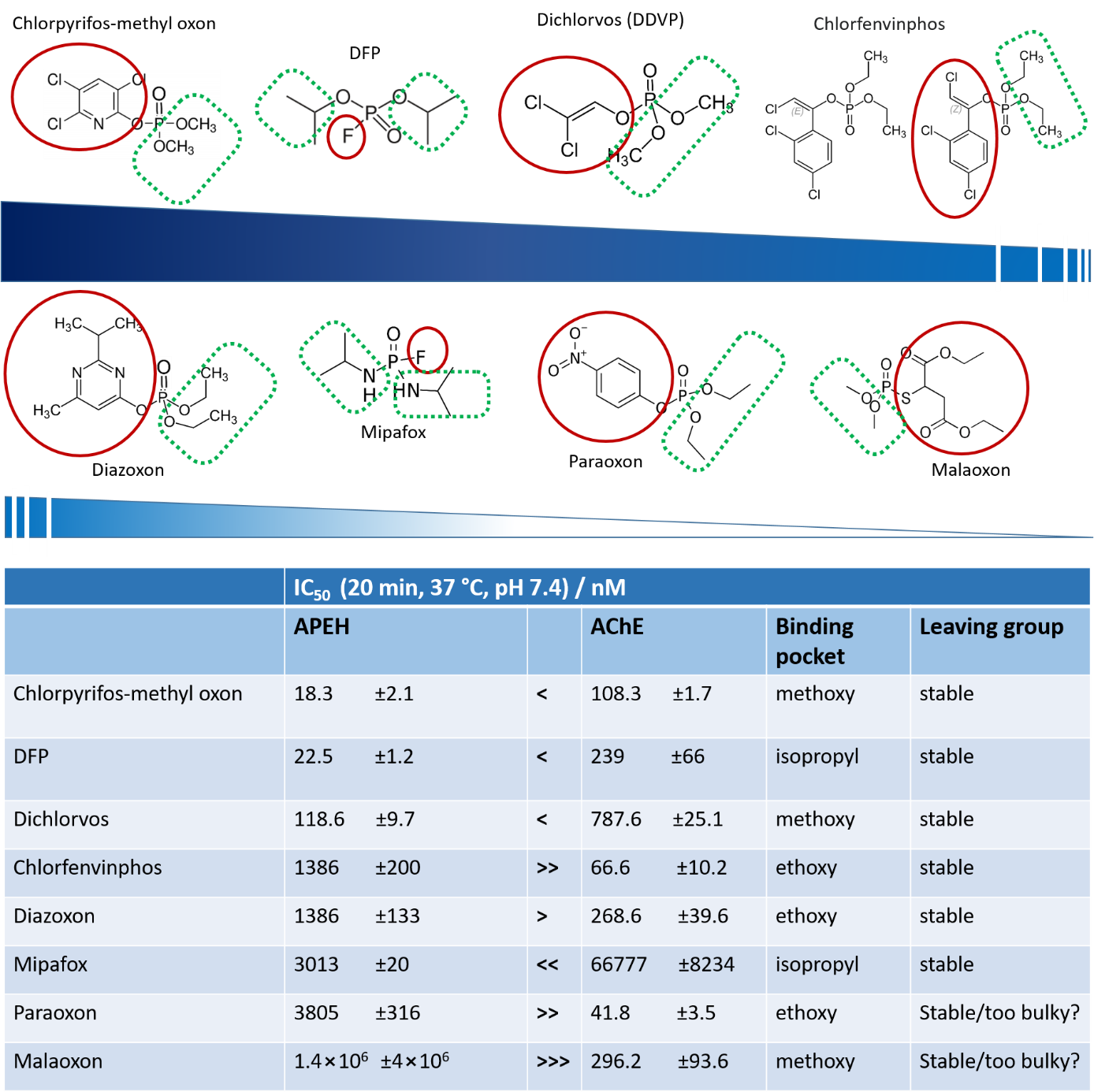


**Table S2. Characteristic distances of the active site of apo-, organophosphate-bound, and CMK-derivatized serine hydrolases of the α/β hydrolase superfamily.** Neither of these enzymes carry Pro in the position corresponding to Pro506 of pAPEH.

| distances (Å) | PDB ID | C_α_-Ser*-- C_α_-His* | C_α_-Ser*-- oxyanion-loop | oxyanion-loop  -- C_α_-His* | PDB ID | C_α_-Ser*-- C_α_-His* | C_α_-Ser*-- oxyanion-loop | oxyanion-loop  -- C_α_-His* |
| --- | --- | --- | --- | --- | --- | --- | --- | --- |
|  | apo form | | | | OP-bound form | | | |
| pAPEH | 7px8_A | 8.4 | 7.1 | 12.6 | **A** | **9.1** | **6.5** | **12.9** |
| pAPEH | 7px8_B | 8.4 | 7.6 | 12.4 | **B** | **9.1** | **6.5** | **12.9** |
| pAPEH | 7px8_C | 8.4 | 7.2 | 12.6 | **C** | **9.0** | **6.5** | **12.9** |
| pAPEH | 7px8_D | 8.5 | 7.1 | 12.7 | **D** | **9.1** | **6.7** | **12.9** |
| acetylcholine-esterase | 1J06_A | 8.0 | 5.8 | 10.3 | 2jgf_A | 8.0 | 5.8 | 10.2 |
| acetylcholine-esterase | 1J06_B | 8.1 | 5.8 | 10.3 | 2jgf_B | 8.0 | 5.8 | 10.3 |
| acetylcholine-esterase |  |  |  |  | 2jgi_A | 8.2 | 5.8 | 10.4 |
| acetylcholine-esterase |  |  |  |  | 2jgi_B | 8.3 | 5.8 | 10.4 |
| acetylcholine-esterase |  |  |  |  | 2jgj_A | 8.1 | 5.8 | 10.4 |
| acetylcholine-esterase |  |  |  |  | 2jgj_B | 8.1 | 5.9 | 10.3 |
| acetylcholine-esterase |  |  |  |  | 2jgk_A | 8.3 | 5.9 | 10.6 |
| acetylcholine-esterase |  |  |  |  | 2jgk_B | 8.4 | 5.8 | 10.3 |
| acetylcholine-esterase |  |  |  |  | 2jgl_A | 8.4 | 5.7 | 10.6 |
| acetylcholine-esterase |  |  |  |  | 2jgl_B | 8.3 | 5.9 | 10.5 |
| acetylcholine-esterase |  |  |  |  | 2jgm_A | 8.3 | 6.0 | 10.6 |
| acetylcholine-esterase |  |  |  |  | 2jgm_B | 8.3 | 5.8 | 10.3 |
| butyrylcholine-esterase | 2pm8_A | 8.3 | 5.8 | 10.7 | 3djy | 8.6 | 5.9 | 11.0 |
| butyrylcholine-esterase | 2pm8_B | 8.0 | 5.3 | 10.4 | 3dkk | 8.5 | 5.9 | 11.0 |
| butyrylcholine-esterase |  |  |  |  | 1p0q | 8.5 | 5.8 | 10.8 |
| butyrylcholine-esterase |  |  |  |  | 1xlw | 8.5 | 5.9 | 10.8 |
| butyrylcholine-esterase |  |  |  |  | 2xqj | 8.5 | 6.0 | 11.0 |
| butyrylcholine-esterase |  |  |  |  | 2xqk | 8.6 | 5.9 | 11.0 |
| butyrylcholine-esterase |  |  |  |  | 4bbz | 8.4 | 6.0 | 10.9 |
| PAF acetylhydrolase | 3d59 | 8.1 | 6.0 | 11.1 | 3d5e | 8.1 | 6.1 | 11.1 |
| PAF acetylhydrolase |  |  |  |  | 3f96 | 8.1 | 6.0 | 11.1 |
| PAF acetylhydrolase |  |  |  |  | 3f97 | 8.0 | 6.1 | 11.1 |
| PAF acetylhydrolase |  |  |  |  | 3f98 | 7.5 | 6.2 | 10.6 |
| PAF acetylhydrolase |  |  |  |  | 3f9c | 7.9 | 6.3 | 11.2 |
| dipeptidyl peptidase IV | 1tk3 | 8.0 | 6.1 | 11.2 | 1tkr | 8.1 | 6.4 | 11.5 |
| carboxylesterase 1 | 2h7c | 8.2 | 5.9 | 10.9 | 2hrq | 8.5 | 5.8 | 11.0 |
| carboxylesterase 1 | 4ab1 | 8.2 | 6.0 | 10.8 | 2hrr | 8.4 | 5.8 | 11.0 |
| carboxylesterase 1 | 5a7f | 8.1 | 5.7 | 10.6 | 3k9b | 8.3 | 5.7 | 11.2 |
| cathepsin A | 1ivy_A | 7.5 | 5.9 | 11.3 | 6wia | 7.6 | 5.9 | 11.3 |
|  | 1ivy_B | 7.4 | 6.0 | 11.4 |  |  |  |  |
|  | 4ci9 | 7.7 | 5.8 | 11.3 |  |  |  |  |
|  | 4mws_A | 7.6 | 5.7 | 11.4 |  |  |  |  |
|  | 4mws_B | 7.6 | 5.6 | 11.4 |  |  |  |  |
|  | 4mwt_A | 7.6 | 5.7 | 11.4 |  |  |  |  |
|  | 4mwt_B | 7.5 | 5.6 | 11.3 |  |  |  |  |
|  | apo-form | | | | CMK-bound form | | | |
| pAPEH | 7px8_A | 8.4 | 7.1 | 12.6 | **A** | **8.0** | **6.3** | **11.4** |
| pAPEH | 7px8_B | 8.4 | 7.6 | 12.4 | **B** | **8.4** | **6.7** | **11.8** |
| pAPEH | 7px8_C | 8.4 | 7.2 | 12.6 | **C** | **8.4** | **6.8** | **11.8** |
| pAPEH | 7px8_D | 8.5 | 7.1 | 12.9 | **D** | **8.3** | **6.8** | **11.9** |
| *Ap*APEH | 3o4g_A | 8.1 | 5.8 | 11.3 | 4re5_A | 8.1 | 6.0 | 11.0 |
| *Ap*APEH | 3o4g_C | 8.1 | 5.8 | 11.3 | 4re5_B | 8.1 | 6.0 | 11.1 |
| *Ap*APEH |  |  |  |  | 4re6_A | 8.0 | 5.9 | 11.0 |
| *Ph*APEH | 4hxe | 7.7 | 6.0 | 11.3 | 4hxf | 7.7 | 5.9 | 11.1 |
| pAPEH model: AF-P19205-F1-v4 | | 8.0 | 5.2 | 10.9 |  |  |  |  |
| hAPEH model: AF-P13798-F1-v4 | | 8.1 | 5.4 | 10.9 |  |  |  |  |
|  | |  |  |  |  |  |  |  |
|  | apo-form | | | | ligand-bound form | | | |
| LysoPLA model: AF-095372-F1 | | 8.2 | 6.0 | 10.8 |  |  |  |  |
|  |  |  |  |  | 5syn_A | 8.4 | 6.3 | 11.1 |
|  |  |  |  |  | 5sym_A | 8.3 | 6.3 | 10.9 |
|  |  |  |  |  | 6bje | 8.5 | 5.7 | 11.1 |
|  | |  |  |  |  |  |  |  |

**Figure S10. The phylogenetic tree of S9 enzyme family created by MEROPS database.**^18,19^ The green box highlights the S9C subfamily (of which APEHs are members), the yellow box indicates the S9C subfamily entries that contain a *Pro* residue corresponding to Pro506 of pAPEH (on the 4^th^ strand of the hydrolase domain core β-sheet) and mammalian enzymes with this particular *Pro* mutation are marked with the blue box. (Light brown rectangles show the exceptions to this mutation.) Note: The MEROPS database contains seprate entries of the same organism with incomplete sequences.


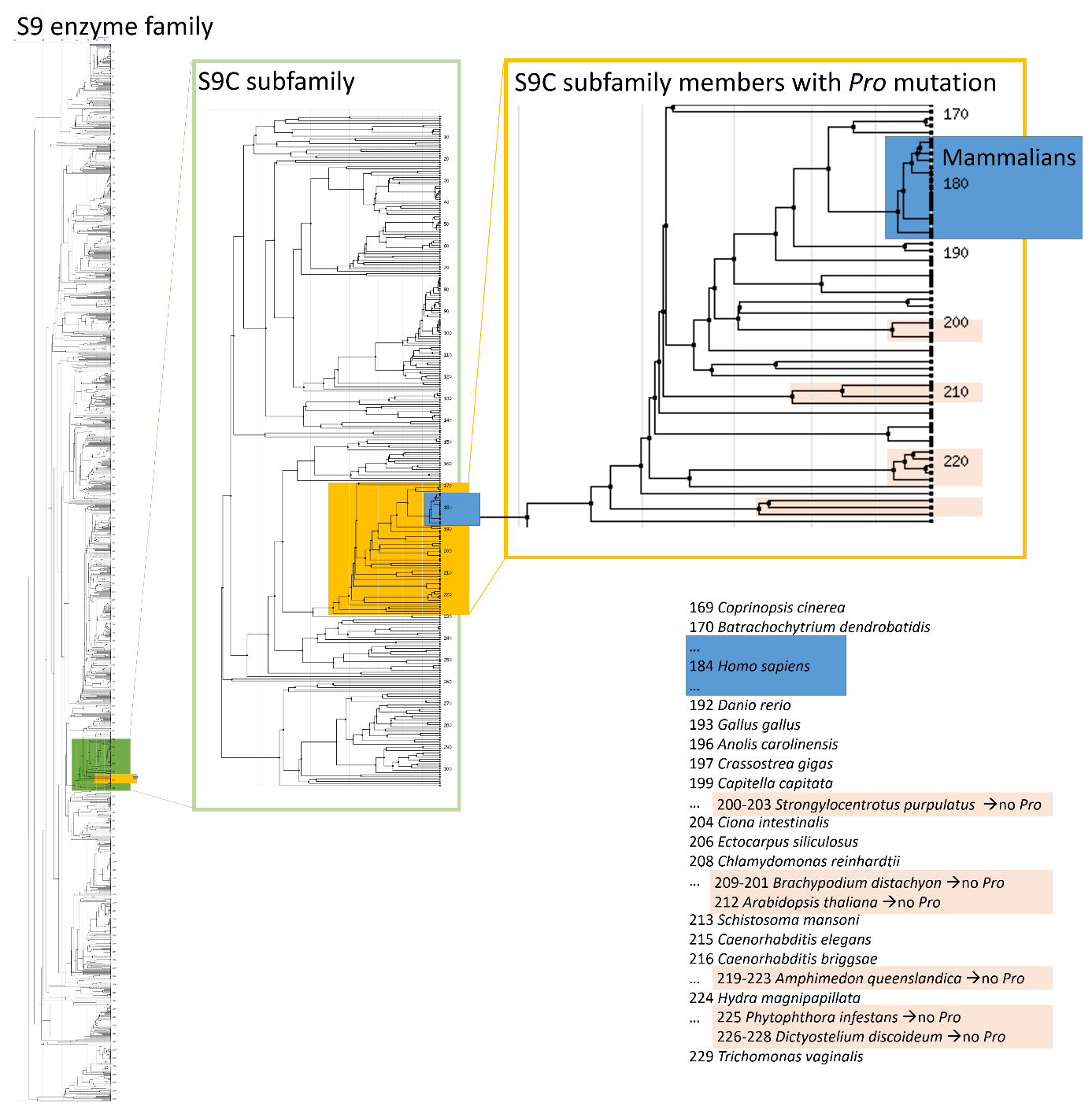


**Figure S11. The phylogenetic tree of the human APEH gene aligned with Ensembl.**^20^ The alignement shows that the APEH gene is present in all domains of life. For a detailed view visit: https://www.ensembl.org/Homo_sapiens/Gene/Compara_Tree?db=core;g=ENSG00000164062;r=3:49674014-49683971^
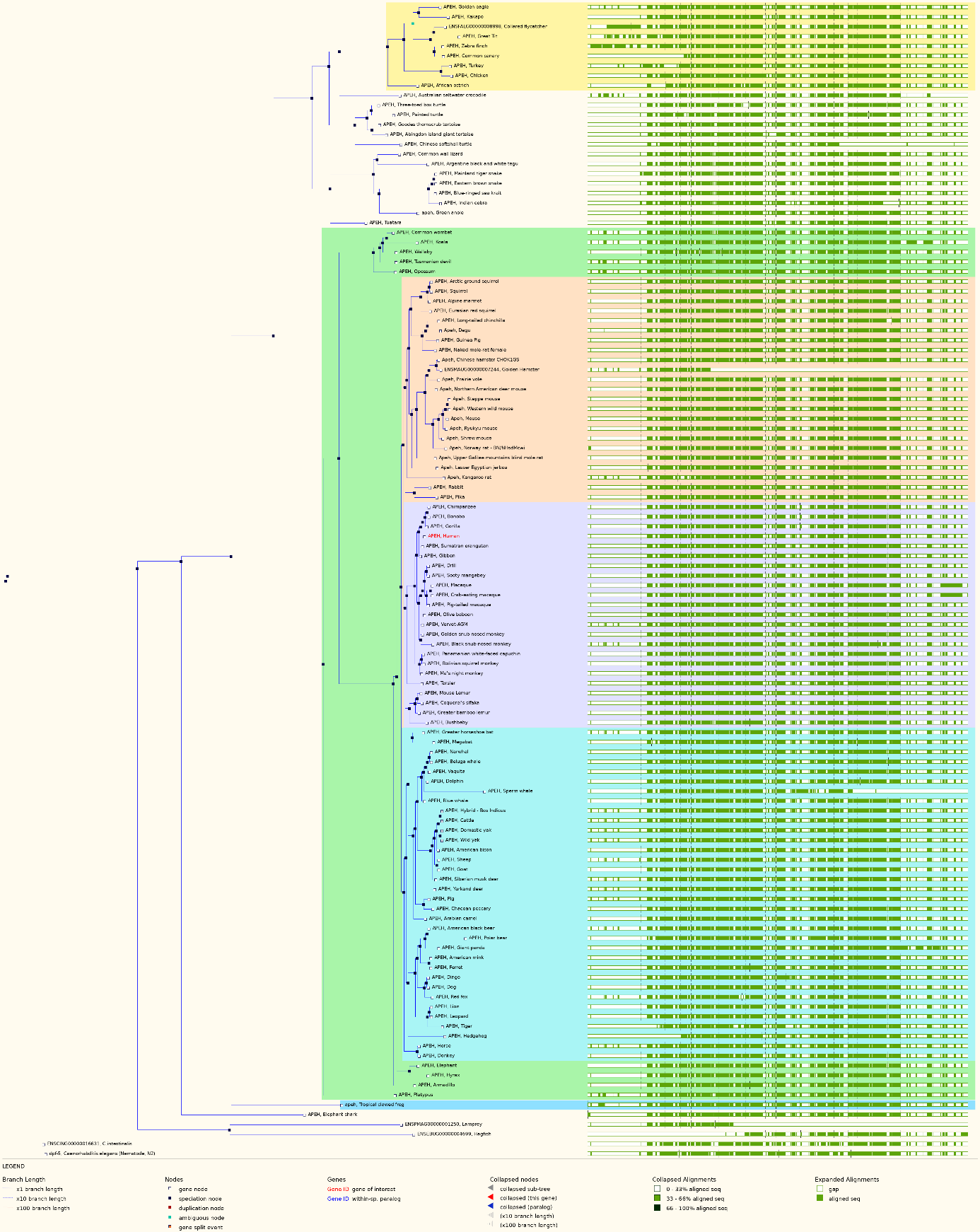
^

**Figure S12. Phylogenetic tree of human APEH, gene alignment and the amino acids at the Pro506 site (pAPEH numbering) and the catalytic Ser.** The gene and protein alignment was conducted with Panther^21^ and visualized with iTol^22^ and WebLogo.^23^ The logos show the site of the strand-breaker Pro of pAPEH and the following residues (blue outline) and the immediate surroundings of the catalyitic Ser (light blue outline). In the logos, orange triangles indicate the residues found at the position of Pro506, purple stars mark the glycine residue of the oxyanion-site (if present) and red asterisks mark the catalytic serine residues. (Interestingly *Ap*APEH and *Ph*APEH are not present in this tree; this might be due to low sequence homology as Panther uses Hidden Markov Models (HMMs) with a cutoff criteria of 30% sequence homology.)

**
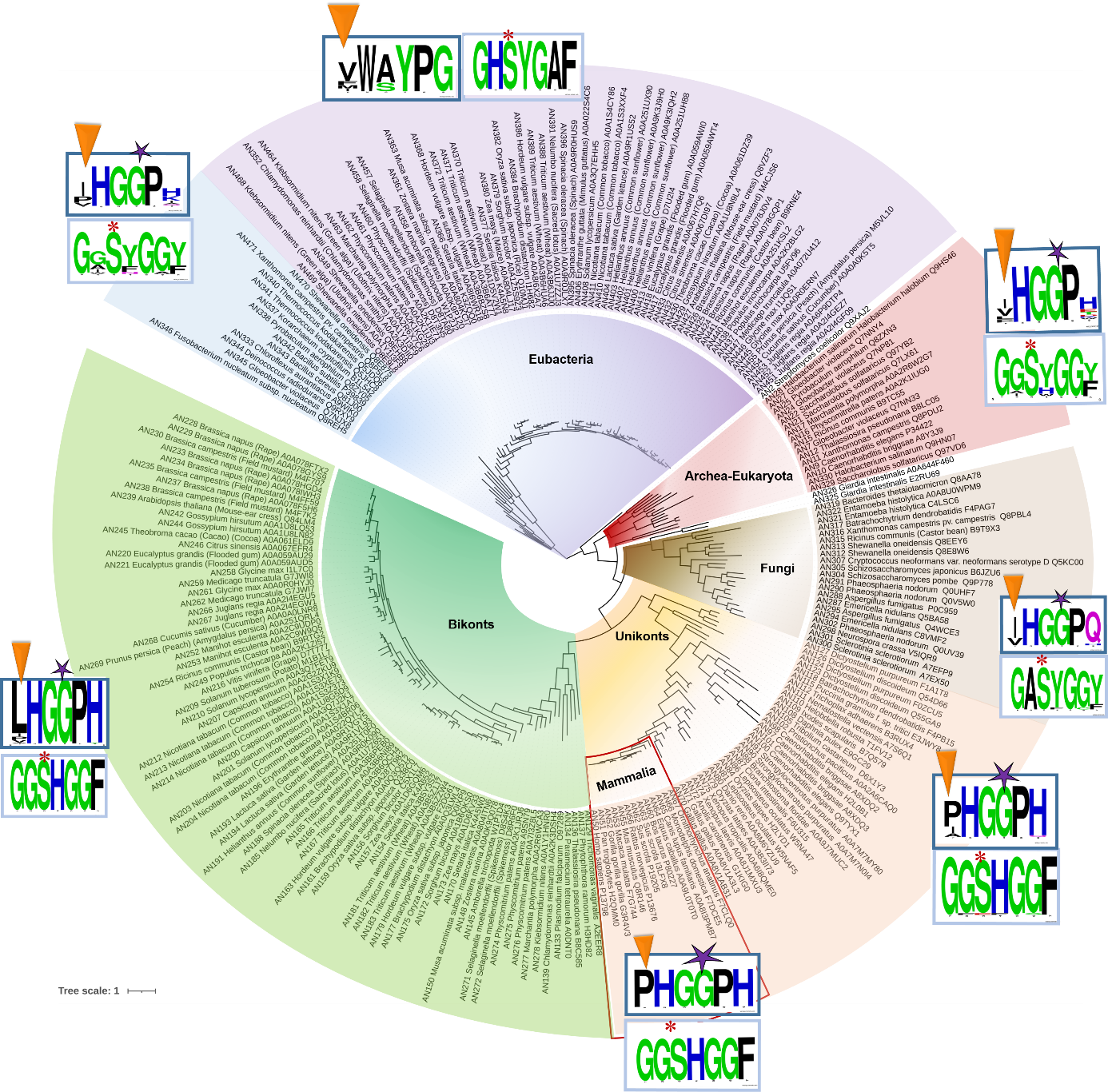
**

Source for the sequences:

<https://pantherdb.org/panther/family.do?clsAccession=PTHR42776:SF4>

<https://pantherdb.org/treeViewer/treeViewer.jsp?book=PTHR42776&sf=SF4&gridMSA=true&msaTab=true>

**Table S3. Characteristic distances of the active site of apo-, OP-, phosphonate- and CMK-derivatized serine proteases of the serine protease clans.^24^**

| **Serine protease clan** | **Protein names**  **(catalytic Ser, basic residue, distal H bond donor of the oxyanion site)** | **PDB ID** | **C_α_-Ser-- C_α_-basic residue** | **C_α_-Ser-- oxyanion-site** | **oxyanion-site-C_α_ -basic residue** | **PDB ID** | | **C_α_-Ser-- C_α_-basic residue** | **C_α_-Ser-- oxyanion-site** | **oxyanion-site**  **-- C_α_-basic residue** |  |
| --- | --- | --- | --- | --- | --- | --- | --- | --- | --- | --- | --- |
|  | | **apo -forms** | | | | | **OP bound forms (in red) and CMK-bound (in blue) forms** | | | | |
| SC | **pAPEH (present study)  (S587, H707, G509)** | **7px8,**  **(chains A-D average)** | **8.5** | **7.2** | **12.6** | **9gne, 9gou (chains A-D averaged)** | | **8.3,**  **9.1** | **6.5,**  **6.7** | **12.9,**  **11.7** |  |
| PA | Chymotrypsin  *Bos taurus*  (S195, H57, G193) | 2gch | 8.3 | 5.4 | 11.4 | 1gcd,  1gmh | | 8.2,  8.3 | 5.4,  5.5 | 11.3,  11.3 |  |
|  | Thrombin  *H.sapiens*  (S195, H57, G193) | 1sg8  (chains A-B average) | 8.4 | 5.5 | 11.6 | 1h8i  (phosphonate-bound) | | 8.3 | 5.5 | 11.2 |  |
|  |  |  |  |  |  | 1ppb  1hai | | 8.3  8.2 | 5.5  5.8 | 11.0  11.1 |  |
|  | Kallikrein related peptidase  *H.sapiens*  (S195, H57, G193) | 5ms3 | 8.6 | 5.4 | 11.6 |  | | | | |  |
| SB | Subtilisin  *Bacillus licheniformis*  (S221, H64, N155) | 3unx | 8.5 | 6.2 | 11.1 | 1bh6 | | 8.2 | 5.8 | 10.5 |  |
| SE | D-alanyl-D-alanine carboxypeptidase  *E.coli*  (S40,K43,T212) | 3it9  (chains A-D average) | 5.5 | 5.7 | 11.0 |  | | | | |  |
| SF | LexA peptidase  *E.coli*  (S119, K156, M118) | 1jhf | 7.3 | 4.3 | 11.3 |  | | | | |  |
| SH | Pseudorabies virus protease pUL26N  *Suid αherpesvirus 1*  (S109,H43,R136) | 4v07  (chains A-B average) | 8.4 | 6.3 | 12.0 | 4v08  (chains A-B average) | | 8.5 | 5.9 | 12.0 |  |
| SJ* | Lon peptidase  *E.coli*  (S679A, K722, P678) | 1rre | 9.9 | 4.3 | 13.0 |  | | | | |  |
| SK | Clp peptidase ab  *E.coli*  (S97, H122, G68) | 1tyf  (chains A-N average) | 5.8 | 6.2 | 11.0 |  | | | | |  |
| SP | Nucleoporin 98 autoproteolytic domain, H. sapiens  (S864, H862, K791*NZ*) | 2q5x | 5.9 | 4.6 | 7.4 |  | | | | |  |
| SS | L,D-Carboxypeptidase  *Pseudomonas aeruginosa*  (S115, H285, G88) | 1zrs  (chains A-B average) | 7.9 | 5.7 | 10.9 |  | | | | |  |
| ST | rhomboid protease GlpG,  *E.coli*  (S201,H254,H150) | 2ic8 | 7.3 | 5.7 | 11.1 | 41hd | | 7.8 | 5.7 | 11.4 |  |

SQ and SR clans is not included due to unsufficient data or different catalytic mechanism.

*catalytic Ser is mutated to Ala therefore the C_α_-Ser*-- C_α_-basic residue distance is lengthened

**Figure S13. Characterization of acetyl-alanyl-chloromethylketone (Ac-Ala-CMK).** RP-HPLC, Zorbax C18 5µM, 150⨯4.6mm, 300 Å pore size. Gradient: 0 min 0% B, 2min. 0% B, 22min. 90% B (eluent A: 0.1% TFA/water, eluent B: 20% 0.1%TFA/water and 80% acetonitrile). Flow 1ml/min. R_t_=11,0min. (ESI-MS Bruker Esquire 3000plus, M_measured_=163 g/mol).


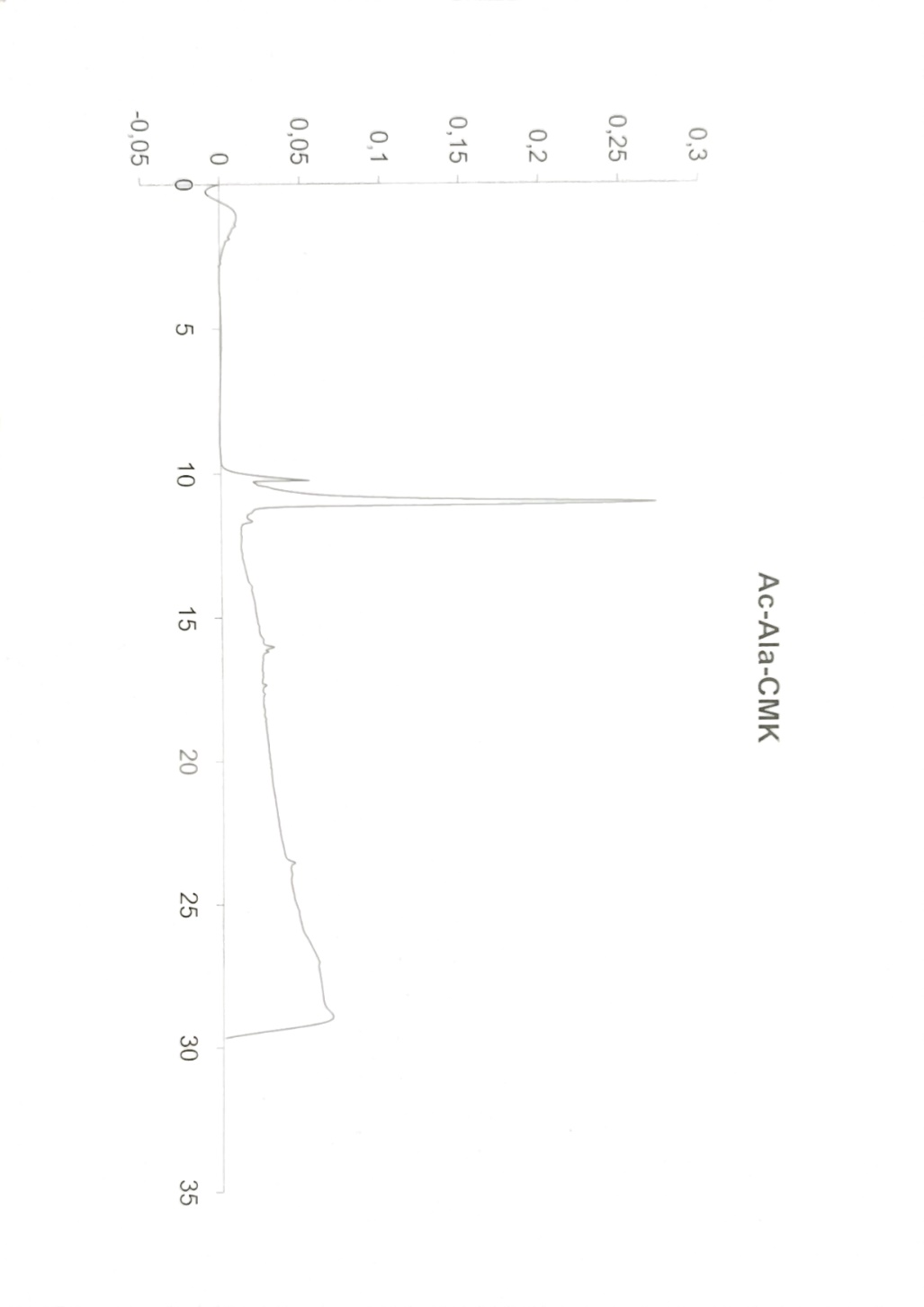


**Figure S14.** **Representative structures of the five clusters (cluster centers) obtained during the short, restrained MD simulation of the pAPEH-AcAMK complex intended to provide starting structures for the subsequent QM/MM calculations.** Dark grey and maroon show the conformer where direct H-bond is formed between the oxyanion loop and the inhibitor (35.1% of the snapshots belong to this cluster) and lighter grey and pink colors indicate those arrangements (4 clusters) where a more relaxed conformation of the oxyanion loop was found. The cryo-EM structure of ligand-free pAPEH (PDB: 7px8) is shown in green.

**
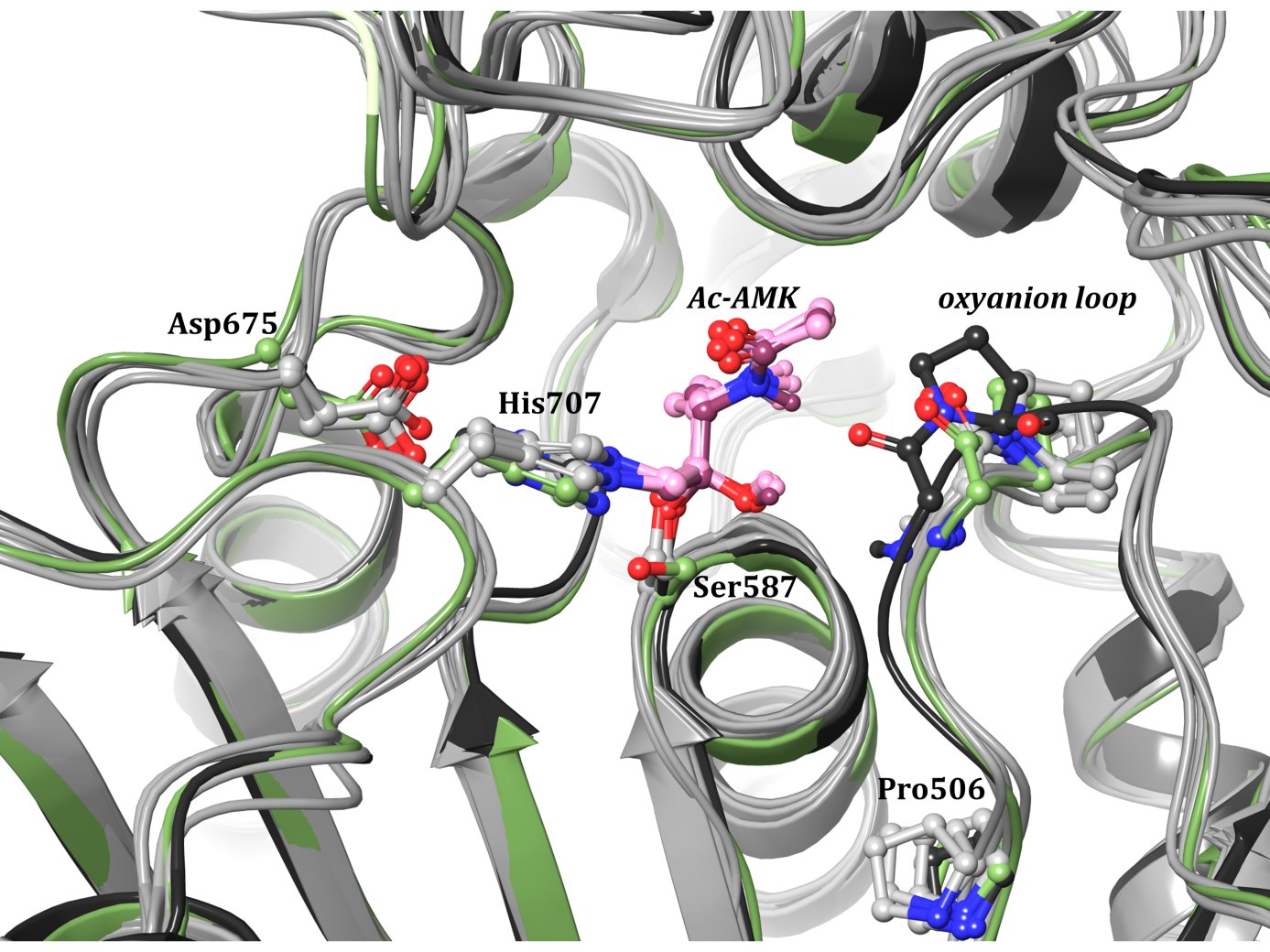
**

References for the Supplementary materials

1. Kim S, Chen J, Cheng T, Gindulyte A, He J, He S, Li Q, Shoemaker BA, Thiessen PA, Yu B, Zaslavsky L, Zhang J, Bolton EE. PubChem 2025 update. *Nucleic Acids Res*. (2025) 53(D1):D1516-D1525. doi: 10.1093/nar/gkae1059. PMID: 39558165.
2. Daina A, Michielin O, Zoete V. SwissTargetPrediction: updated data and new features for efficient prediction of protein targets of small molecules. *Nucleic Acids Res*. (2019) 47(W1):W357-W364. doi: 10.1093/nar/gkz382. PMID: 31106366.
3. Keiser MJ, Roth BL, Armbruster BN, Ernsberger P, Irwin JJ, Shoichet BK. Relating protein pharmacology by ligand chemistry. *Nat Biotechnol*. (2007) 25(2):197-206. doi: 10.1038/nbt1284. PMID: 17287757.
4. Wang X, Shen Y, Wang S, Li S, Zhang W, Liu X, Lai L, Pei J, Li H. PharmMapper 2017 update: a web server for potential drug target identification with a comprehensive target pharmacophore database. *Nucleic Acids Res*. (2017) 45(W1):W356-W360. doi: 10.1093/nar/gkx374. PMID: 28472422.
5. Tsoupras AB, Chini M, Tsogas N, Lioni A, Tsekes G, Demopoulos CA, Lazanas MC. In vitro anti-inflammatory and anti-coagulant effects of antibiotics towards Platelet Activating Factor and thrombin. *J Inflamm (Lond)*. (2011) 8:17. doi: 10.1186/1476-9255-8-17. PMID: 21736752.
6. Kiss AL, Hornung B, Rádi K, Gengeliczki Z, Sztáray B, Juhász T, Szeltner Z, Harmat V, Polgár L. The acylaminoacyl peptidase from Aeropyrum pernix K1 thought to be an exopeptidase displays endopeptidase activity. *J Mol Biol.* (2007) 368(2):509-20. doi: 10.1016/j.jmb.2007.02.025. PMID: 17350041.).
7. Menyhárd DK, Orgován Z, Szeltner Z, Szamosi I, Harmat V. Catalytically distinct states captured in a crystal lattice: the substrate-bound and scavenger states of acylaminoacyl peptidase and their implications for functionality. *Acta Cryst.* (2015). D71, 461-472 doi: 10.1107/S1399004714026819. PMID: 25760596
8. Harmat V, Domokos K, Menyhárd DK, Palló A, Szeltner Z, Szamosi I, Beke-Somfai T, Náray-Szabó G, Polgár L. Structure and catalysis of acylaminoacyl peptidase: closed and open subunits of a dimer oligopeptidase. *J Biol Chem*. (2011) 286(3):1987-98. doi: 10.1074/jbc.M110.169862. PMID: 21084296.
9. Cianci M, Bourenkov G, Pompidor G, Karpics I, Kallio J, Bento I, Roessle M, Cipriani F, Fiedler S, Schneider TR. P13, the EMBL macromolecular crystallography beamline at the low-emittance PETRA III ring for high- and low-energy phasing with variable beam focusing. *J Synch. Rad*. 2017 24:323–332. doi: 10.1107/S1600577516016465. PMID: 28009574
10. Kabsch W. XDS. *Acta Crystallogr. D Biol. Crystallogr*. (2010) 66: 125–132. doi: 10.1107/S0907444909047337
11. Liebschner D, Afonine PV, Baker ML, Bunkóczi G, Chen VB, Croll TI, Hintze B, Hung LW, Jain S, McCoy AJ, Moriarty NW, Oeffner RD, Poon BK, Prisant MG, Read RJ, Richardson JS, Richardson DC, Sammito, MD, Sobolev OV, Stockwell DH, Terwilliger TC, Urzhumtsev AG, Videau LL, Williams CJ, Adams PD. Macromolecular Structure Determination Using X-Rays, Neutrons and Electrons: Recent Developments in Phenix. *Acta Crystallogr. Sect. Struct. Biol.* (2019) 75: 861–877. doi: 10.1107/S2059798319011471.
12. Afonine PV, Grosse-Kunstleve RW, Echols N, Headd JJ,; Moriarty NW,; Mustyakimov M, Terwilliger TC, Urzhumtsev A, Zwart PH, Adams PD. Towards Automated Crystallographic Structure Refinement with Phenix.Refine. *Acta Crystallogr. D Biol. Crystallogr.* (2012) 68: 352–367. doi: 10.1107/S0907444912001308.
13. Williams CJ, Headd JJ, Moriarty NW, Prisant MG, Videau LL, Deis LN, Verma V, Keedy DA, Hintze BJ, Chen VB, Jain S, Lewis SM, Arendall III WB, Snoeyink J, Adams PD, Lovell, SC, Richardson JS, Richardson DC. MolProbity: More and Better Reference Data for Improved All-Atom Structure Validation. *Protein Sci*. (2018) 27: 293–315. doi: 10.1002/pro.3330.
14. Emsley P, Lohkamp B, Scott WG, Cowtan K. Features and Development of Coot. *Acta Crystallogr. D Biol. Crystallogr*. (2010) 66: 486–501. doi: 10.1107/S0907444910007493.
15. Richards P, Johnson M, Ray D, Walker C. Novel protein targets for organophosphorus compounds. *Chem Biol Interact*. (1999) 119-120:503-11. doi: 10.1016/s0009-2797(99)00064-2. PMID: 10421489.
16. Richards PG, Johnson MK, Ray DE. Identification of acylpeptide hydrolase as a sensitive site for reaction with organophosphorus compounds and a potential target for cognitive enhancing drugs. *Mol Pharmacol*. (2000) 58(3):577-83. doi: 10.1124/mol.58.3.577. PMID: 10953051.
17. Hörnberg A, Tunemalm AK, Ekström F. Crystal structures of acetylcholinesterase in complex with organophosphorus compounds suggest that the acyl pocket modulates the aging reaction by precluding the formation of the trigonal bipyramidal transition state. *Biochemistry.* (2007) 46(16):4815-25. doi: 10.1021/bi0621361. PMID: 17402711.
18. <https://www.ebi.ac.uk/merops/cgi-bin/famwrap/famcards/trees/s9_tree.htm>
19. <https://www.ebi.ac.uk/merops/cgi-bin/famwrap/famcards/trees/s9c_tree.htm>
20. Sarah C Dyer, et al. **Ensembl 2025** *Nucleic Acids Res. (*2025) 53(D1):D948–D957, PMID: 39656687.
21. Thomas PD, Ebert D, Muruganujan A, Mushayahama T, Albou LP, Mi H. PANTHER: Making genome-scale phylogenetics accessible to all. *Protein Sci*. (2022) 31(1):8-22. doi: 10.1002/pro.4218. PMID: 34717010.
22. Letunic I, Bork P. Interactive Tree of Life (iTOL) v6: recent updates to the phylogenetic tree display and annotation tool. *Nucleic Acids Res*. (2024) 52(W1):W78-W82. doi: 10.1093/nar/gkae268. PMID: 38613393.
23. Crooks GE, Hon G, Chandonia JM, Brenner SE. WebLogo: a sequence logo generator. *Genome Res*. (2004) 14(6):1188-90. doi: 10.1101/gr.849004. PMID: 15173120.
24. Di Cera E. Serine proteases. *IUBMB Life*. (2009) 61(5):510-5. doi: 10.1002/iub.186. PMID: 19180666.
